# Experimental and theoretical insights in the alkene–arene intramolecular π-stacking interaction

**DOI:** 10.3762/bjoc.12.158

**Published:** 2016-07-28

**Authors:** Valeria Corne, Ariel M Sarotti, Carmen Ramirez de Arellano, Rolando A Spanevello, Alejandra G Suárez

**Affiliations:** 1 Instituto de Química Rosario, Facultad de Ciencias Bioquímicas y Farmacéuticas, Universidad Nacional de Rosario-CONICET. Suipacha 531, RosarioS2002LRK, Argentina; 2 Departamento de Química Orgánica, Universidad de Valencia, Valencia 46100, Spain

**Keywords:** acrylic esters, asymmetric synthesis, biomass, conformational equilibrium, π-stacking interaction

## Abstract

Chiral acrylic esters derived from biomass were developed as models to have a better insight in the aryl–vinyl π-stacking interactions. Quantum chemical calculations, NMR studies and experimental evidences demonstrated the presence of equilibriums of at least four different conformations: π-stacked and face-to-edge, each of them in an *s-cis/s-trans* conformation. The results show that the stabilization produced by the π–π interaction makes the π-stacked conformation predominant in solution and this stabilization is slightly affected by the electron density of the aromatic counterpart.

## Introduction

**Figure 1:**
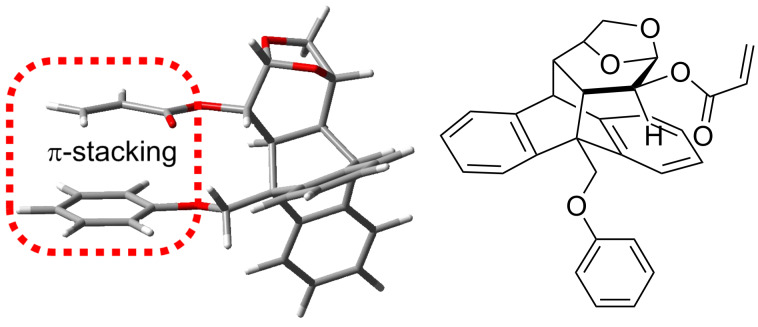
Intramolecular aryl–vinyl π-stacking interaction of a levoglucosenone derivative.

Noncovalent interactions have demonstrated to have relevant importance in chemistry and biology [1–4]. Considering all noncovalent interactions, π-stacking is perhaps the less well understood, although its application in materials sciences, enzyme design and template-directed synthesis have had a dramatic growth, mainly for arene–arene interactions [1–8]. In particular, the use of π–π overlap as an element of stereocontrol in highly selective chemical transformations has been widely explored [9]. In this context, as part of our continuous interest in the development of new tools for asymmetric synthesis using levoglucosenone (a biomass-derived chiral enone) [10–15], we reported the synthesis of a novel chiral auxiliary that demonstrated to be very efficient in an asymmetric Diels–Alder reaction between the acrylic ester derivative and cyclopentadiene [16]. The facial selectivity of this reaction proved to be due to an aryl–vinyl π-stacking intramolecular interaction that fixed the conformation of the dienophile (Figure 1) [16].

Given this precedent, we aimed to gain further insight into this type of interaction, by exploring the effect caused by the introduction of electronically different substituents at the aromatic ring of the chiral auxiliary. For this reason, we planned to synthesize the acrylic esters, structurally related to the previously reported acrylate, with CF_3_ and OMe groups in the *para* position of the phenoxy group as representative electron-withdrawing and releasing groups, respectively. The π–π interaction was studied by combining experimental, spectroscopic and computational evidence of the structurally related chiral acrylates to get new insights into the alkene–arene intramolecular interaction. Besides, it was evaluated the effect of the electron density of the aromatic counterpart and its influence on the asymmetric inductive capacity of the auxiliary.

## Results and Discussion

**Scheme 1:**
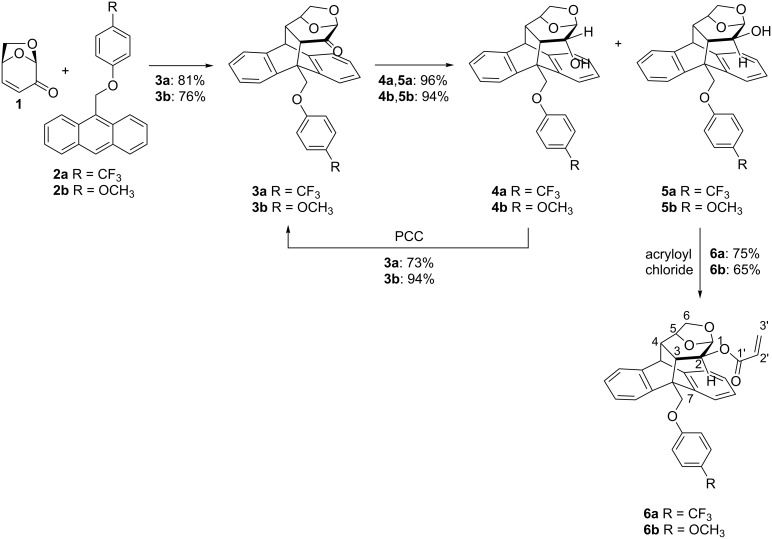
Synthesis of acrylates **6a**,**b**.

The synthesis of acrylate derivatives **6a**,**b** bearing CF_3_ and OMe substituents in the *para* position of the phenoxy group is depicted in Scheme 1. Levoglucosenone (**1**) was used as a dienophile to react with the corresponding 9-substituted anthracenes **2a**,**b**, which are easily prepared from commercially available 9-anthracenyl methanol. When the Diels–Alder reactions were carried out in refluxing toluene, the desired ortho adducts **3a** and **3b** were obtained in good yields of 71% and 86%, respectively. However, the long reaction times (8–9 days) prompted us to evaluate these chemical transformations under microwave irradiation [17], affording the cycloadducts **3a**,**b** in very good yields (76–81%) after irradiating a THF solution of **1** and **2** at 150 °C during 4 hours. The reduction of the ketone group in **3** with NaBH_4_ produced alcohols **4** and **5** in excellent yields and a ratio of about 40:60. The diastereomeric mixture of **4** and **5** was easily separated by flash chromatography. Finally, acrylates **6a**,**b** were prepared by reaction of the corresponding alcohol **5** with acryloyl chloride in the presence of Et_3_N at 0 °C. The corresponding acrylic ester could not be obtained from the epimeric alcohols **4**, probably because of the steric hindrance surrounding the alcohol position. Therefore, alcohols **4** were recycled by oxidation with PCC regenerating **3** in very good yields, and increasing the overall yield of acrylates **6**.

**Figure 2:**
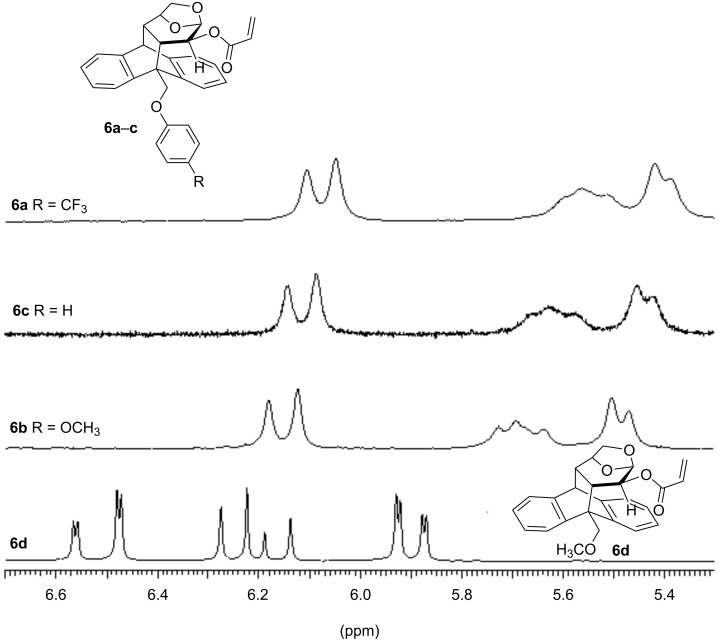
Vinyl region of the ^1^H NMR spectra of **6a**–**d** in CDCl_3_ at 300 K.

The ^1^H NMR spectra of compounds **6a** and **6b** (300 MHz, CDCl_3_) showed an important shielding effect of the vinylic protons at 6.10–5.38 and 6.18–5.47 ppm, respectively (Figure 2), compared to those of analogous acrylates without an aromatic substituent at the benzylic position (6.57–5.87 ppm) [12–13]. The magnitude of this shielding, slightly more pronounced for **6a**, was in the range of a previous reported acrylate with a pendant phenoxy group **6c** (6.14–5.41 ppm) [16]. This effect is interpreted in terms of the anisotropy phenomena caused by the proximity of the aromatic ring to the double bond. Another salient observation is the broadening of the vinylic signals that can be associated to a dynamic process taking place at room temperature.

**Figure 3:**
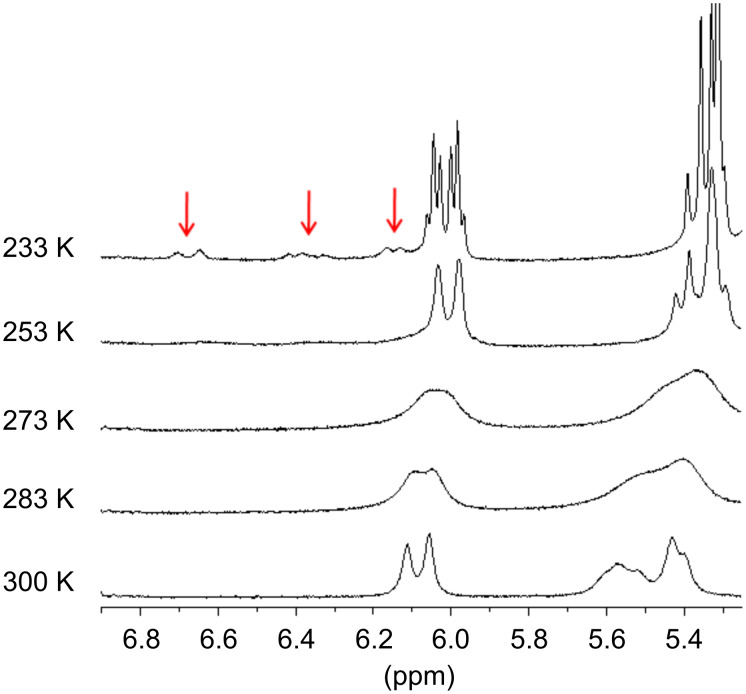
Vinylic region of the low temperatures ^1^H NMR spectra of **6a** in CDCl_3_.

In order to unravel the origin of this phenomenon, we further carried out low temperature NMR studies (Figure 3). When the CDCl_3_ solution of acrylate **6a** was cooled gradually from 300 K the resonances corresponding to the vinylic protons broaden (283 and 273 K) and at 233 K two different shielding zones were detected for these protons at 6.70–6.13 and 6.06–5.30 ppm (Figure 3 and for further details, see Supporting Information File 1). This phenomenon could be interpreted in terms of the existence of 2 species present in solution in approximately a 9:1 ratio, according to the relative area of the signals. Some authors have claimed that this effect could be due to a conformational equilibrium of *s*-*cis* and *s*-*trans* species that is shifted toward the *s-trans* conformer at lower temperatures [18–21].

Similar studies were carried out for acrylates **6b** and **6c** demonstrating analogous behaviors. Although the low temperature NMR studies for acrylate **6c** were already reported, it was not perceived the presence of the second set of vinylic signals between 6.70 and 6.10 ppm at 233 K [16].

**Figure 4:**
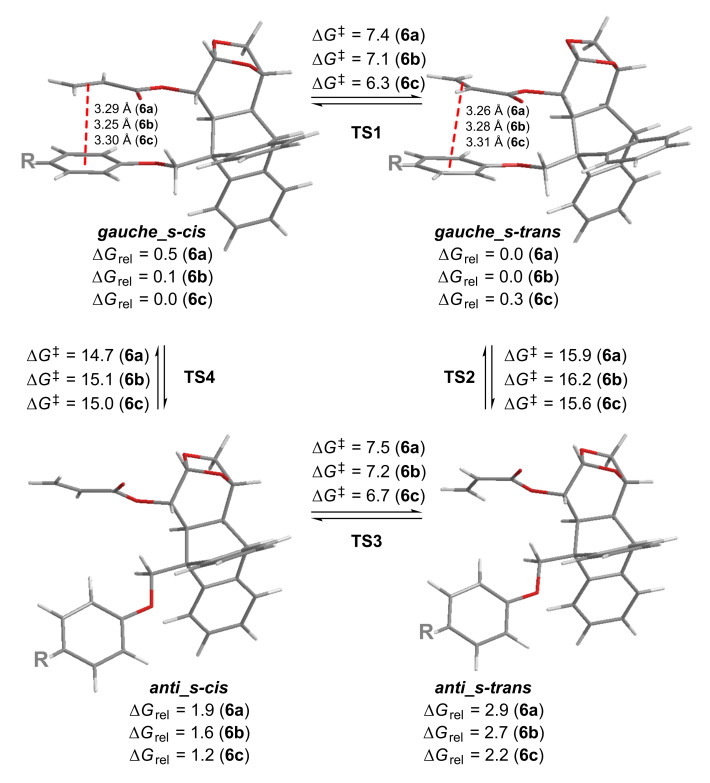
M06-2X/6-31+G(d) Gibbs free energy profiles (in kcal/mol) computed for the conformational equilibriums of **6a**–**c**.

In order to have a better understanding of this conformational dynamics, we further carried out a detailed computational study at the M06-2X level of theory [22] for the three acrylates **6a**–**c**. This meta-GGA functional, developed by Truhlar [23] has been shown to be one of the approaches of choice to model noncovalent interactions [9]. After a complete exploration of the potential energy surface (PES) at the M06-2X/6-31+G(d) level of theory, four local minima were located for each system, namely: *gauche_s-cis*, *gauche_s-trans*, *anti_s-cis* and *anti_s-trans* (Figure 4) [24]. In the *gauche* rotamers (the most stable found in each system), the phenoxy and acrylate moieties lie parallel to each other, and the distances from the centroid of the aromatic ring to the midpoint of the C=C double bond are in the range of 3.25–3.31 Å, consistent with the distances for other π-stacked systems [2]. On the other hand, in the *anti* conformers the remoteness of the phenyl and alkene groups prevents any stabilizing interaction between them. For the gauche conformations, NBO analysis [25–26] (at the M06-2X/6-31+G(d) level) revealed a two-electron stabilizing donation from the π orbital of the phenoxy ring to the π* anti-bonding orbital of the acrylate moiety, supporting the through-space interaction between these fragments. The associated second order perturbation energies (Δ*E*^(2)^) are in the range of −0.90 to −0.73 kcal/mol for *6-gauche_s-cis* and −0.84 to −0.69 kcal/mol for *6-gauche_s-trans*. The energy (ZPE) difference between gauche and *anti* conformations (Δ*E*_ag_* = E*_anti_ − *E*_gauche_) can be seen as a measurement of the stabilization caused by the intramolecular interaction. The Δ*E*_ag_ values computed for all acrylate derivatives **6** show that Δ*E*_ag_ for acrylate **6a** is higher than that for **6b** and **6c** (3.91 kcal/mol vs 3.49 kcal/mol vs 3.27 kcal/mol, respectively), suggesting a slightly stronger interaction for **6a** (see Supporting Information File 1 for further details).

**Scheme 2:**
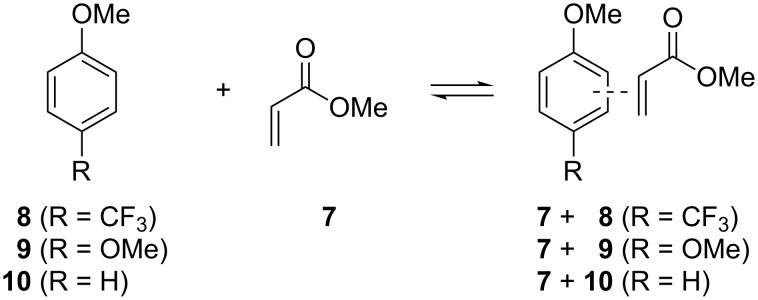
Complexes between methyl acrylate (**7**) and representative anisole derivatives.

To shed light on these issues, we next computed the interaction energies for the complexes of methyl acrylate (**7**) and 4-trifluoromethylanisole (**8**), 4-methoxyanisole (**9**) or anisole (**10**), (Scheme 2).

For the complexes in *s-cis* conformation, the π–π interaction energy computed at the M06-2X/6-31+G(d) level is −7.2 kcal/mol for **7** + **8**, −6.9 kcal/mol for **7** + **9** and −6.5 kcal/mol for **7** + **10** (similar values were computed for **7** in its *s-trans* conformation, see Supporting Information File 1). Similar trends were found at the MP2/6-31+G(d) level of theory. NBO calculations indicated that in these π-stacked complexes, the charge transfer (CT) takes place from the aromatic compound to the acrylate. Despite the amount of CT is higher for the more electron-rich ring **9** (0.013e vs 0.011e (**8**) vs 0.008e (**10**)), according to the results presented herein, the extra stabilization of the CF_3_ substituted systems cannot be due to polar or charge-transfer effects but rather from dispersion interactions [27]. In an effort to prove this hypothesis, we performed an energy decomposition analysis (EDA) at the B3LYP-D3/TZ2P level using ADF. In this approach, the interaction energy (Δ*E*_i_) is decomposed as the sum of four terms [28]: Δ*E*_i_ = Δ*V*_elstat_ + Δ*E*_Pauli_ + Δ*E*_oi_ + Δ*E*_disp_, where Δ*V*_elstat_ accounts for the usually attractive classical electrostatic interaction between the deformed fragments, Δ*E*_Pauli_, the Pauli repulsion term, represents the destabilizing interactions between the occupied orbitals (steric repulsion), Δ*E*_oi_ shows the interaction between orbitals (including all electron-pair bondings, HOMO–LUMO charge-transfer processes, polarization interactions, etc.), and Δ*E*_disp_ refers to the effect exerted by dispersion forces. As shown in Table 1 a slightly higher interaction energy was found for **7** + **8** (−7.7 kcal/mol). It is important to point out the great significance of the dispersion forces in these π-stacked complexes, representing ≈60% of the total attractive forces.

**Table 1:** Energy decomposition analysis (EDA) of the three complexes shown in Scheme 2.

	**7** + **8**	**7** + **9**	**7** + **10**

Δ*E*_i_	−7.7	−7.2	−6.8
Δ*E*_Pauli_	17.9	18.2	17.7
Δ*V*_elstat_	−9.8 (38%)	−9.5 (37%)	−9.3 (36%)
Δ*E*_oi_	−3.8 (16%)	−4.1 (17%)	−3.9 (17%)
Δ*E*_disp_	−12.0 (61%)	−11.8 (60%)	−11.3 (57%)

Noteworthy, the distances from the centroid of the aromatic ring to the midpoint of the double bond, calculated at both levels of theory for the complexes of **7** and anisole derivatives **8**–**10** are between 3.23–3.29 Å which is in excellent agreement with the distances calculated for the π-stacked conformations of **6a**–**c** shown in Figure 4.

**Figure 5:**
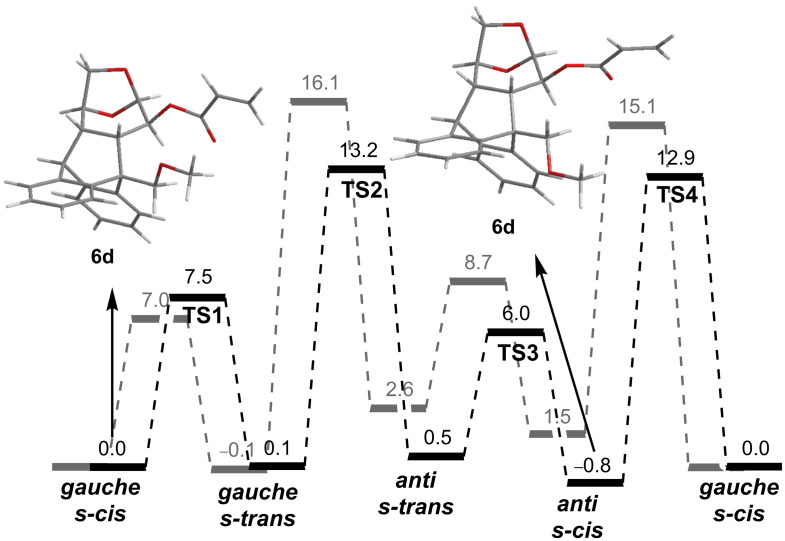
Comparison of the M06-2X/6-31+G(d) energy profiles (in kcal/mol) computed for **6d** and **6b** (in grey).

As depicted in Figure 4, the energy barriers computed for the *s-cis/s-trans* conversion of **6a**–**c** are low (6.3–7.5 kcal/mol), suggesting a fast equilibration even at low temperatures. On the other hand, the barriers calculated for the *anti/gauche* interconversion are considerably higher (14.7–16.2 kcal/mol). This slow conformational change can be responsible not only for the broadening of the NMR signals of some nuclei at room temperature, but also for the NMR splitting observed upon cooling. In the *gauche* conformers a significant shielding of the vinyl protons is expected, while in the *anti* rotamers those nuclei should not be affected by the ring current exerted by the aromatic substituent. In addition, the relative intensity of these two sets of NMR signals should be related to the Boltzmann distribution of both types of conformations. Accordingly, the *gauche/anti* ratios computed from the Gibbs free energies are 97:3 (**6a**), 95:5 (**6b**) and 91:9 (**6c**) in very good agreement with the average 9:1 value experimentally obtained from the low temperature NMR spectra. Moreover, the higher population of the π-stacked conformers found for **6a** accounts for the magnitude of the shielding of the acrylate protons (Figure 2). In an additional effort to understand the extent to which the π–π interactions affect the conformational equilibriums of a system, we next explored the M06-2X/6-31+G(d) PES of acrylate **6d** bearing a methoxymethyl group at the benzylic position of the molecule (Figure 5). With the lack of stabilizing π-stacking, both *gauche* and *anti* conformations are of similar energy. In fact, the *anti_s-cis* rotamer is the global minima. Moreover, while the *s-cis/s-trans* conversion is still predicted to be very fast, the barriers computed for the *gauche/anti* equilibration are lower (≈13 kcal/mol), reinforcing the fact that the π-stacking interaction also affects the equilibrium kinetics.

**Figure 6:**
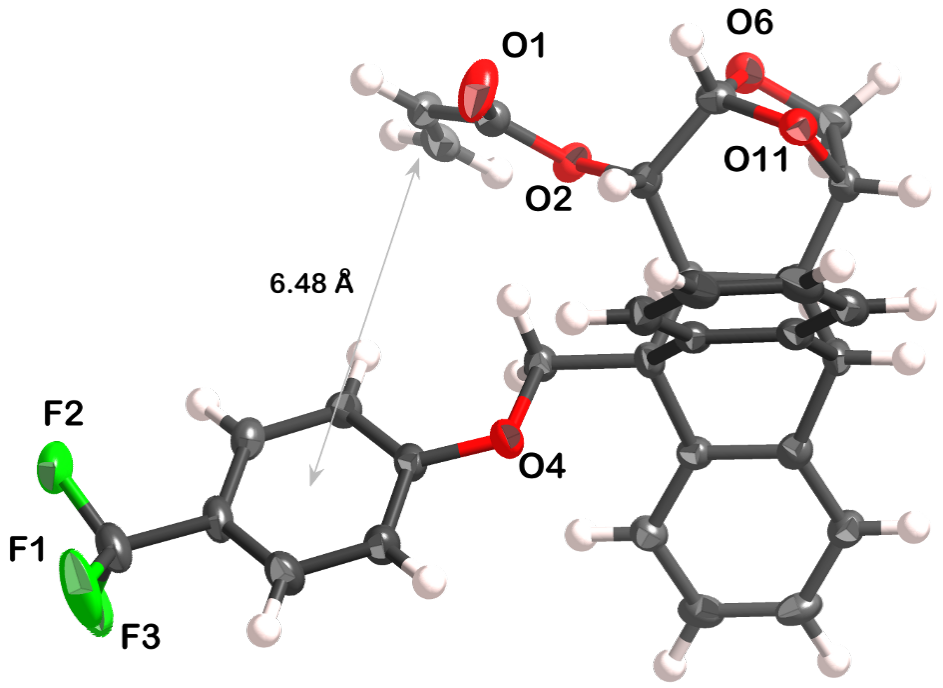
X-ray thermal ellipsoid plot of **6a** (50% probability level) showing the labeling scheme (hydrogen and carbon labels have been omitted for clarity).

In order to better understand the conformational studies, a crystal of acrylate **6a** suitable for X-ray diffraction analysis was obtained (for further information, see Supporting Information File 1). The crystal structure shows the vinyl and aromatic groups in a *face-to-edge* arrangement, with a dihedral angle of 73.8(2)° between the groups’ mean planes (Figure 6 and Supporting Information File 1). The distance between the centroid of the arene and the middle point of the C=C double bond is 6.48 Å. The conformation found in the solid state is in full agreement with the calculated structure 6a*-anti_s-trans* (Figure 4). This *face-to-edge* conformation accounts for the resonance signals of the vinylic hydrogens of the minor species detected at 233 K in the ^1^H NMR spectrum. It is assumed that the minor species was the one found in the crystal structure due to crystal lattice stabilization.

These experimental and theoretical results led us to postulate that acrylates **6a**–**c** are present in a conformational equilibrium between at least 4 different species: π-*stacked* and *face-to-edge*, and each of them in an *s-cis/s-trans* conformation. This equilibrium is affected by the strength of the π–π interaction, which is slightly higher for the electron-withdrawing substituted phenoxy ring. The lower amount of the minor *face-to-edge* species detected in the low temperature ^1^H NMR spectrum of **6a**, along with the more shielded protons of the vinyl group at room temperature, account for this explanation.

With the aim to determine if the strength of the intramolecular π-*stacking* interaction can have any influence in the inductive capacity, we studied the Diels–Alder reaction of acrylates **6a**,**b** with cyclopentadiene (Table 2).

**Table 2:** Thermal and Lewis acid promoted Diels–Alder reactions of **6a**,**b** and cyclopentadiene.

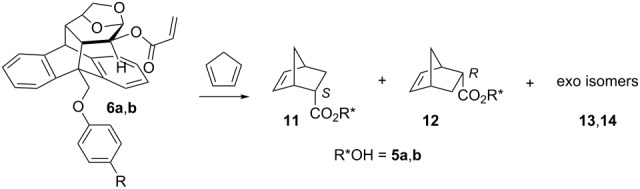							

Entry	Acrylate	Lewis acid	*T* (°C)	*t* (h)	Yield (%)^a^	*endo*/*exo*^b^	*endo R*/*S*^b^

1	**6a**	–	25	144	92	80:20	12:88
2	**6a**	Et_2_AlCl	0	1	75	92:8	92:8
3	**6b**	–	25	96	92	78:22	18:82
4	**6b**	Et_2_AlCl	0	1	80	92:8	94:6

^a^Yield corresponds to isolated products. ^b^Determined by HPLC.

All cycloadditions were *endo* diastereoselective as predicted by the Alder´s rule. The reaction between **6a** or **6b** and cyclopentadiene carried out at room temperature showed a high stereoselectivity (Table 2, entries 1 and 3, respectively). These results are similar to the ones previously reported for **6c** (*endo R*/*S* 13:87) [16]. Noticeably, the *endo S*/*R* ratios are considerable high in the absence of Lewis acids. The high inductive capacity is interpreted in terms of the fact that the conformation of the dienophile is fixed by a π-stacking interaction. The reactions promoted by Et_2_AlCl produced the reversed *endo S/R* diastereoselectivity with a concomitant increase in the *endo*/*exo* ratio, as found for other related systems [11,13,16]. These results were interpreted in terms of the formation of an aluminum-chelated species with the acryloyl oxygen and the oxygen of the 1,6-anhydro bridge, according to the NMR studies (see Supporting Information File 1). Saponification of adducts **11** and **12** provided the free 5-norbornenecarboxylic acid and the chiral auxiliaries **5a** and **5b** in excellent yields, which can be reused.

## Conclusion

We have reported the synthesis of chiral acrylates derived from renewable feedstock as models to study arene–alkene π-stacking interactions. To the best of our knowledge this is the first report that provides experimental and computational evidences to understand the effect of such intramolecular interactions in the conformational dynamics of the system. This equilibrium, that involves a fast *s-cis*/*s-trans* exchange and a slow π-stacked/face-to-edge conversion, depends on the strength of the π–π interaction, which is slightly higher when the arene moiety is substituted with an electron-withdrawing group. The results presented herein will allow for better understanding the intramolecular interactions between alkenes and aromatic rings. This can be useful in several fields, such as supramolecular chemistry, biology and material science and, in particular, in the area of asymmetric synthesis for the rational design of new elements of stereocontrol.

## Supporting Information

File 1 Experimental procedures, characterization and spectral data for synthesized compounds and X-ray data for compound **6a**.
