# Supplementary material for: Experimental and theoretical insights in the alkene–arene intramolecular π-stacking interaction
Source: Beilstein J Org Chem. 2016 Jul 28;12:1616–23. doi: 10.3762/bjoc.12.158 (PMC4979656; doi:10.3762/bjoc.12.158)
Supplement: File 1 — Experimental procedures, characterization and spectral data for synthesized compounds and X-ray data for compound 6a. [file Beilstein_J_Org_Chem-12-1616-s001.pdf]

Supporting Information

for

Experimental and theoretical insights in the alkene–arene intramolecular π-stacking interaction

Valeria Corne[1], Ariel M. Sarotti[1], Carmen Ramirez de Arellano[2], Rolando A. Spanevello[1], and Alejandra G. Suárez[*1]

Address: [1]Instituto de Química Rosario, Facultad de Ciencias Bioquímicas y Farmacéuticas, Universidad Nacional de Rosario-CONICET. Suipacha 531, RosarioS2002LRK, Argentina and [2]Departamento de Química Orgánica, Universidad de Valencia, Valencia 46100, Spain

Email: Alejandra G. Suárez - asuarez@fbioyf.unr.edu.ar

*Corresponding author

Experimental procedures, characterization and spectral data for the synthesized compounds and X-ray data for compound **6a**

General Information:

The melting points were taken on a Leitz Wetzlar Microscope Heating Stage Model 350 apparatus and are uncorrected. Optical rotations were recorded with a Jasco DIP 1000 digital polarimeter. Infrared spectra were obtained on an IRPrestige-21 Fourier Transform Infrared Spectrophotometer from Shimadzu. High resolution mass spectra (HRMS) were obtained on a Bruker micrOTOF-Q II LC–MS spectrometer. HPLC analyses were performed with a Varian ProStar chromatograph equipped with UV–vis detector from ProStar 320 and monitored at 270 nm. HPLC was performed on a Beckman Ultrasphere C-18 column (250 x 4.6 mm). Acetonitrile and water HPLC grade were used as eluents in a mixture of 80:20 or 70:30, respectively and at a flow rate of 1 mL/min or 1.5 mL/min. Nuclear magnetic resonance spectra were recorded on a Bruker Avance-300 DPX spectrometer with tetramethylsilane as internal standard and deuterochloroform as the solvent. The NMR assignments were corroborated by NOE measurements, H,H- and H,C-correlations. Variable-temperature ^{1}H NMR spectra were recorded on a Bruker Avance-300 DPX spectrometer with tetramethylsilane as internal standard and deuterochloroform as solvent. Chiral GC–MS analyses were performed on a Perkin Elmer AutoSystem XL gas chromatograph coupled to a TurboMass spectrometer using a capillary column (Astec CHIRALDEX™ G-TA, 30 m, 0.25 mm i.d., 0.12 µm film thickness).

The reactions were monitored by thin layer chromatography using precoated silica gel plates from Merck (0.25 mm, 60F254) that were developed using UV light and anisaldehyde/sulfuric acid/acetic acid with subsequent heating. Flash column chromatography was performed using Merck silica gel 60H, with gradient elution using mixtures of hexanes and increasing amounts of ethyl acetate or dichloromethane. All reactions were carried out under argon atmosphere with dry, freshly distilled solvents under anhydrous conditions unless otherwise noted. Yields refer to chromatographically and spectroscopically (^{1}H NMR) homogeneous materials, unless otherwise stated.

Levoglucosenone (1)

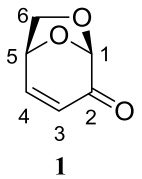

1

Levoglucosenone was synthesized according to the procedure described in literature [1].

1: Yellow oil; $[\alpha]_D^{25}$ = -562,3 (*c* 1,04, $CHCl_3$); IR (film) 3631, 2966, 2899, 1712 (C=O), 1693, 1379, 1107, 972, 891, 854, 831 cm^{-1}; ^{1}H NMR (300 MHz, $CDCl_3$) δ 7.29 (dd, $J_{3\text{-}4}$= 10.0 Hz, $J_{4\text{-}5}$= 4.7 Hz, 1 H, H-4), 6.12 (dd, $J_{3\text{-}4}$= 10.0 Hz, $J_{1\text{-}3}$=1.6 Hz, 1 H, H-3), 5.36 (d, $J_{1\text{-}3}$=1.6 Hz, 1 H, H-1), 5.02 (dd, $J_{4\text{-}5}$= $J_{5\text{-}6exo}$= 4.7 Hz, 1 H, H-5), 3.91 (dd, J_{gem}= 6.8 Hz, $J_{5\text{-}6exo}$= 4.7 Hz, 1 H, H-6*exo*), 3.78

[1] a) Sarotti, A. M; Spanevello, R. A.; Suárez, A. G. *Green Chemistry,* **2007**, *9*, 1137-1140. b) Witczak , Z. J. (Ed.), *Levoglucosenone and Levoglucosans: Chemistry and Applications*, ATL Press, Mount Prospect, **1994**. Chapter 2. (C. Morin)

(d, J_{gem}= 6.8 Hz, 1 H, H-6*endo*); ^{13}C NMR (75.5 MHz, $CDCl_3$) δ 188.7 (C, C-2), 147.9 (CH, C-4), 126.7 (CH, C-3), 101.5 (CH, C-1), 71.6 (CH, C-5), 66.4 (CH_2, C-6).

9-[(4-(Trifluoromethyl)phenoxy)methyl]anthracene (2a)

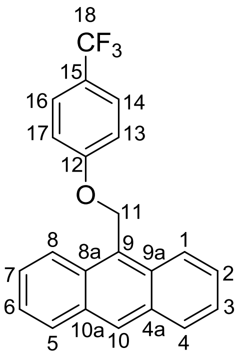

2a

Analogous as described in [2], anthracen-9-ylmethanol (97%, 1560 mg, 7.25 mmol) was dissolved in dry CH_2Cl_2 (3.3 mL) and dry benzene (9.7 mL) under argon. $SOCl_2$ was added (0.7 mL, 9.60 mmol) and the solution was stirred at reflux overnight under argon. The reaction mixture was poured into ice–water (25 mL). The organic phase was washed with water (3 × 15 mL), dried (Na_2SO_4) and concentrated. The residual solid was dissolved in dry acetone (35 mL), 4-trifluoromethylphenol (97%, 1800 mg, 10.77 mmol) and anhydrous K_2CO_3 99% (1530 mg, 10.98 mmol) were added and the solution was stirred at reflux for 22 h under argon. The solvent was evaporated under reduced pressure and water (100 mL) and CH_2Cl_2 (100 mL) were added. The aqueous phase was extracted with CH_2Cl_2 (3 × 50 mL). The organic phase was washed with saturated Na_2CO_3 (1 × 50 mL), brine (1 × 50 mL), dried (Na_2SO_4) and concentrated. The residual solid was purified by flash chromatography to afford **2a** (1837 mg, 5.21 mmol, 72%).

2a: Colorless crystalline solid; mp 177-178 ºC (Hexane-AcOEt); IR (KBr) 3054, 1586, 1326, 1246, 1112, 839 cm^{-1}; ^{1}H NMR (300 MHz, $CDCl_3$) δ 8.50 (s, 1 H, H-10), 8.19 (d, $J_{1\text{-}2}$= $J_{7\text{-}8}$= 8.4 Hz, 2 H, H-1 and H-8), 8.01 (dd, $J_{3\text{-}4}$= $J_{5\text{-}6}$= 7.8 Hz, $J_{2\text{-}4}$= $J_{5\text{-}7}$= 1.5 Hz, 2 H, H-4 and H-5), 7.61-7.59 (m, 2 H, H-14 and H-16), 7.54-7.44 (m, 4 H, H-2 , H-3, H-6 and H-7), 7.16-7.13 (m, 2 H, H-13 and H-17), 5.90 (s, 2 H, H-11); ^{13}C NMR (75.5 MHz, $CDCl_3$) δ 161.5 (C, C-12) , 131.3 (C, 2 C, C-4a and C-10a), 130.8 (C, 2 C, C-8a and C-9a), 129.2 (CH, C-10), 129.1 (CH, C-4, C-5), 126.9 (CH, $J_{C\text{-}F}$ = 3.6, 2 C, C-14 and C-16), 126.6 (CH, 2 C, C-2 and C-7)*, 125.8 (C, C-9), 125.0 (CH, 2 C, C-3 and C-6)*, 124.4 (C, $J_{C\text{-}F}$ = 271.1, C-18), 123.5 (CH, C-1, C-8), 123.2 (C, $J_{C\text{-}F}$ = 32.7 , C-15), 114.6 (CH, 2 C, C-13 and C-17), 62.8 (CH_2, C-11); ^{19}F NMR (282.4 MHz, $CDCl_3$) δ -61.5; HRMS calc. for $C_{22}H_{15}F_3ONa$ $[M+Na]^+$ 375.0967. Found 375.0954.

---

[2] Sarotti, A. M.; Fernández, I.; Spanevello, R. A.; Sierra, M. Á.; Suárez, A. G. *Org. Lett.* **2008,** *10*, 3389–3392.

Ketone 3a

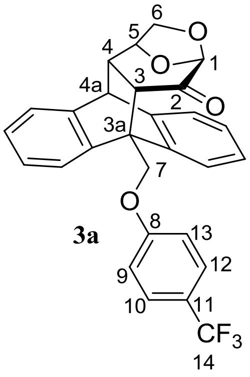

3a

Method 1: Thermal conditions

In a similar manner as described in [2], levoglucosenone (281 mg, 2.23 mmol) and **2a** (1336 mg, 3.79 mmol) were dissolved in toluene (11 mL) at rt and the obtained solution was heated under reflux for 9 d. The solvent was evaparated under vacuum and the solid residue was purified by flash chromatography to give **3a** (757 mg, 1.58 mmol, 71%).

Method 2: Microwave conditions

Levoglucosenone (65 mg, 0.52 mmol) and **2a** (365 mg, 1.04 mmol) were placed in a 10 mL vial and dissolved in THF (0.22 mL). The obtained solution was heated at 150 °C for 4 h. The solvent was evaporated under reduced pressure and the residue was purified by flash chromatography to give **3a** (203 mg, 0.42 mmol, 81%).

3a: Colorless crystalline solid; mp 217-219 ºC (Hexane-AcOEt); $[\alpha]^{28}_{D}$ -86.9 (*c* 0.97, $CHCl_3$); IR (KBr) 3021, 2953, 1720 (C=O), 1327, 1263, 1113, 1107, 836 cm^{-1} ; ^1H NMR (300 MHz, $CDCl_3$) δ 7.66-7.63 (m, 2 H, H-10 and H-12), 7.45-7.10 (m, 10 H, 8 H aromatics, H-9 and H-13), 5.60 (d, J_{gem}= 9.0 Hz, 1 H, H-7), 5.28 (d, J_{gem}= 9.0 Hz, 1 H, H-7), 4.83 (d, $J_{5\text{-}6exo}$= 5.0 Hz, 1 H, H-5), 4.57 (s, 1 H, H-1), 4.37 (d, $J_{4\text{-}4a}$= 1.23 Hz, 1 H, H-4a), 3.77 (dd, J_{gem}=7.2 Hz, $J_{5\text{-}6exo}$= 5.0 Hz, 1 H, H-6_{exo}), 3.70 (d, J_{gem}= 7.2 Hz, 1 H, H-6_{endo}), 3.24 (d, $J_{4\text{-}3}$= 9.3 Hz, 1 H, H-3), 2.37 (d, $J_{4\text{-}3}$= 9.3 Hz, 1 H, H-4); ^{13}C NMR (75.5 MHz, $CDCl_3$) δ 198.9 (C, C-2), 161.0 (C, C-8), 145.0 (C, aromatic), 140.6 (C, aromatic), 140.5 (C, aromatic), 139.7 (C, aromatic), 126.9 (CH, $J_{C\text{-}F}$= 3.5 Hz, 2 C, C-10 and C-12), 126.6 (CH, aromatic), 126.3 (CH, aromatic), 126.1 (CH, aromatic), 126.0 (CH, aromatic), 125.2 (CH, aromatic), 124.4 (C, $J_{C\text{-}F}$= 271.1 Hz, C-14), 123.8 (CH, aromatic), 123.3 (C, $J_{C\text{-}F}$= 32.8 Hz, C-11), 122.4 (CH, aromatic), 121.4 (CH, aromatic), 114.8 (CH, 2 C, C-9 and C-13), 99.7 (CH, C-1), 77.1 (CH, C-5), 68.9 (CH_2, C-6), 66.5 (CH_2, C-7), 50.2 (CH, C-4a), 49.4 (C, C-3a), 44.7 (CH, C-4), 44.2 (CH, C-3); ^{19}F NMR (282.4 MHz, $CDCl_3$) δ -61.4; HRMS calc. for $C_{28}H_{22}F_3O_4$ $[M+H]^+$ 479.1465. Found 479.1447.

Reduction of ketone 3a: alcohols 4a and 5a

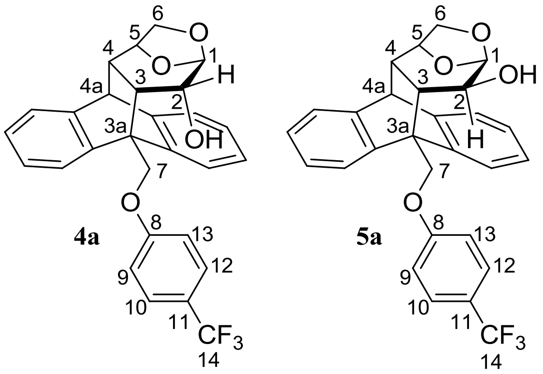

Analogous as described in [2], ketone **3a** (736 mg, 1.54 mmol) was dissolved in a CH_2Cl_2/MeOH 97:3 mixture (35 mL) at rt and $NaBH_4$ (60 mg, 1.59 mmol) was added. The mixture was stirred for 23 h and then acetone (7 mL) was added and the solution was filtered through a short pad of celite. The residue was purified by flash chromatography to afford **4a** (315 mg, 0.66 mmol, 43%) and **5a** (393 mg, 0.82 mmol, 53%).

4a: colorless crystalline solid; mp 233-234 ºC (Hexane-CH_2Cl_2); $[\alpha]_D^{28}$ = +58.6 (*c* 1.02, $CHCl_3$); IR (KBr) 3554 (OH), 3074, 2946, 1590, 1331, 1257, 1110, 836, 747 cm^{-1}; ^1H NMR (300 MHz, $CDCl_3$) δ 7.65-7.62 (m, 2 H, H-10 and H-12), 7.47-7.42 (m, 1 H, aromatic), 7.32-7.05 (m, 9 H, 7 H aromatics, H-9 and H-13), 5.55 (d, J_{gem}=9.1 Hz, 1 H, H-7), 5.34 (d, J_{gem}=9.1 Hz, 1 H, H-7), 4.98 (s, 1 H, H-1), 4.67 (d, $J_{5\text{-}6exo}$= 3.8 Hz, 1 H, H-5), 4.19 (s, 1 H, H-4a), 3.86 (dd, $J_{2\text{-}OH}$=13.4 Hz, $J_{2\text{-}3}$=8.1 Hz, 1 H, H-2), 3.74-3.66 (m, 2 H, H-6), 2.91 (dd, $J_{4\text{-}3}$=10.8 Hz, $J_{2\text{-}3}$=8.1 Hz, 1 H, H-3), 2.06 (d, $J_{4\text{-}3}$=10.8 Hz, 1 H, H-4), 0.71 (d, $J_{2\text{-}OH}$=13.4 Hz, 1 H, OH); ^{13}C NMR (75.5 MHz, $CDCl_3$) δ 161.2 (C, C-8), 146.9 (C, aromatic), 143.1 (C, aromatic), 141.7 (C, aromatic), 141.0 (C, aromatic), 127.0 (CH, $J_{C\text{-}F}$= 3.5 Hz, 2 C, C-10 and C-12), 125.9 (CH, aromatic), 125.7 (CH, 3 C, aromatics), 125.2 (CH, aromatic), 124.3 (C, $J_{C\text{-}F}$= 271.1 Hz, C-14), 123.2 (C, $J_{C\text{-}F}$= 32.7 Hz, C-11), 123.1 (CH, aromatic), 122.7 (CH, aromatic), 122.0 (CH, aromatic), 114.6 (CH, 2 C, C-9 and C-13), 102.0 (CH, C-1), 76.2 (CH, C-5), 72.0 (CH_2, C-6), 68.1 (CH, C-2), 66.7 (CH_2, C-7), 50.7 (CH, C-4a), 49.4 (C, C-3a), 44.9 (CH, C-4), 34.4 (CH, C-3); ^{19}F NMR (282.4 MHz, $CDCl_3$) δ -61.4; HRMS calc. for $C_{28}H_{23}F_3O_4Na$ $[M+Na]^+$ 503.1441. Found 503.1441.

5a: Colorless crystalline solid; mp 244-245 ºC (Benzene); $[\alpha]_D^{26}$ = -12.2 (*c* 0.98, $CHCl_3$); IR (KBr) 3447 (OH), 3071, 2949, 1589, 1329, 1256, 1111, 839 cm^{-1}; ^1H NMR (300 MHz, $CDCl_3$) δ 7.65-7.62 (m, 2 H, H-10 and H-12), 7.42-7.35 (m, 2 H, aromatics), 7.27-7.11 (m, 8 H, 6 H aromatics, H-9 and H-13), 5.19 (d, J_{gem}=9.9 Hz, 1 H, H-7), 5.06 (d, J_{gem}=9.9 Hz, 1 H, H-7), 4.96 (d, $J_{1\text{-}2}$=3.3 Hz, 1 H, H-1), 4.71 (d, $J_{5\text{-}6exo}$= 4.9 Hz, 1 H, H-5), 4.23 (s, 1 H, H-4a), 3.76 (dd, J_{gem}=7.2 Hz, $J_{5\text{-}6}$=4.9 Hz, 1 H, H-6*exo*), 3.66 (d, J_{gem}=7.2 Hz, H-6*endo*), 2.95 (ddd, $J_{2\text{-}OH}$=11.3 Hz, $J_{2\text{-}3}$=5.9 Hz, $J_{1\text{-}2}$=3.2 Hz, 1 H, H-2), 2.30-2.25 (m, 1 H, H-3), 2.13 (d, $J_{4\text{-}3}$=10.4 Hz, 1 H, H-4), 1.87 (d, $J_{2\text{-}OH}$=11.3g Hz, 1 H, OH); ^{13}C NMR (75.5 MHz, $CDCl_3$) δ 161.0 (C, C-8), 146.2 (C, arom), 140.9 (C, arom), 140.9 (C, arom), 140.0 (C, aromatic), 127.0 (CH, $J_{C\text{-}F}$= 3.6 Hz, 2 C, C-10 and C-12,), 126.4 (CH, aromatic), 125.9 (CH, aromatic), 125.8 (CH, aromatic), 125.7 (CH, aromatic), 125.2 (CH, aromatic), 124.3 (C, $J_{C\text{-}F}$= 271.2 Hz, C-14), 123.7 (CH, aromatic), 123.3 (C, $J_{C\text{-}F}$= 32.8 Hz, C-11), 123.0 (CH, aromatic), 122.1 (CH, aromatic), 114.7 (CH, 2 C, C-9 and C-13), 99.9 (CH, C-1), 77.1 (CH, C-5), 70.1 (CH_2, C-6), 68.7 (CH, C-2), 66.9 (CH_2, C-7), 50.2 (CH, C-4a), 50.2 (C, C-3a), 47.6

(CH, C-4), 42.0 (CH, C-3); ^{19}F NMR (282.4 MHz, $CDCl_3$) δ -61.5; HRMS calc. for $C_{28}H_{23}F_3O_4Na$ $[M+Na]^+$ 503.1441. Found 503.1430.

Oxidation of alcohol 4a

In a similar manner as described in [2], alcohol **4a** (403 mg, 0.84 mmol) was dissolved in dry CH_2Cl_2 (20 mL) and PCC (548 mg, 2.54 mmol) was added in one portion and the mixture was stirred for 24 h under an argon atmosphere. Afterwards, the reaction mixture was diluted with CH_2Cl_2 and filtered through a sintered glass funnel containing Florisil®. The filtrate was concentrated to give pure ketone **3a** (376 mg, 0.79 mmol, 94%).

Preparation of acrylate 6a

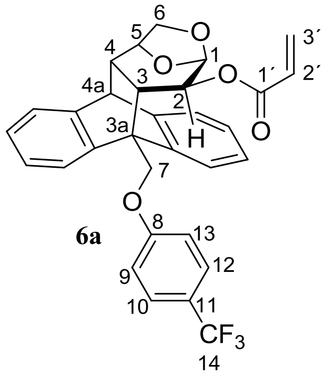

6a

Analogous as described in [2], alcohol **5a** (393 mg, 0.82 mmol) was dissolved in dry CH_2Cl_2 (19 mL) and cooled to 0 °C. Dry triethylamine (500 µL, 3.59 mmol) and acryloyl chloride (161 µL, 1.98 mmol) were added. The mixture was stirred for 1 h under an argon atmosphere and then a few drops of water were added. The solution was dried (Na_2SO_4), concentrated and the residue was purified by flash chromatography to give **6a** (330 mg, 0.62 mmol, 75%).

6a: Colorless crystalline solid; mp 64-65 ºC (Benzene); $[\alpha]_D^{31}$ = -24.2 (*c* 1.02, $CHCl_3$); IR (KBr) 3073, 2956, 1718 (C=O), 1591, 1331, 1256, 1111, 834, 749 cm^{-1}; ^{1}H NMR (300 MHz, $CDCl_3$) δ 7.59-7.56 (m, 2 H, H-10 and H-12), 7.45-7.35 (m, 2 H, aromatics), 7.30-7.20 (m, 4 H, aromatics), 7.16-7.07 (m, 4 H, 2 H aromatics, H-9 and H-13), 6.08 (d, J_{vec}= 16.1 Hz, 1 H, H-3′*cis*), 5,55 (dd, J_{vec}= 16.1 Hz, J_{vec}= 9.3 Hz, 1 H, H-2′), 5,40 (d, J_{vec}= 9.3 Hz, 1 H, H-3′*trans*), 5.08-5.04 (m, 2 H, H-1 and H-7), 4.73-4.69 (m, 2 H, H-5 and H-7), 4.38 (dd, $J_{2\text{-}3}$=6.3 Hz, $J_{1\text{-}2}$=3.3 Hz, 1 H, H-2), 4.25 (s, 1 H, H-4a), 3.76-3.75 (m, 2 H, H-6), 2.90 (m, 1 H, H-3), 2.28 (d, $J_{3\text{-}4}$=106 Hz, 1 H, H-4); ^{13}C NMR (75.5 MHz, $CDCl_3$) δ 165.2 (C, C-1′), 160.8 (C, C-8), 145.9 (C, aromatic), 141.0 (C, aromatic), 140.3 (C, aromatic), 140,0 (C, aromatic), 130.9 (CH_2, C-3′), 126.9 (CH, $J_{C\text{-}F}$= 3.6 Hz, 2 C, C-10 and C-12), 126.6 (CH, 2 C, CH aromatic and C-2′), 126.1 (CH, 2 C, aromatic), 125.9 (CH, aromatic), 125.2 (CH, aromatic), 124.3 (C, $J_{C\text{-}F}$= 271.2 Hz, C-14), 123.7 (CH, aromatic), 123.3 (C, $J_{C\text{-}F}$= 32.7 Hz, C-11), 122.3 (CH, aromatic), 122.2 (CH, aromatic), 114.4 (CH, 2 C, C-9 and C-13), 97.0 (CH, C-1), 76.6 (CH, C-5), 70.4 (2C, CH_2, C-6 and CH, C-2), 66.0 (CH_2, C-7), 50.4 (C, C-4a), 49.5 (CH, C-3a), 47.3 (CH, C-4), 36.4 (CH, C-3); ^{19}F NMR (282.4 MHz, $CDCl_3$) δ -61.5; HRMS calc. for $C_{31}H_{25}F_3O_5Na$ $[M+Na]^+$ 557.1546. Found 557.1540.

X-ray data for compound **6a**: colourless prism, 0.15 x 0.10 x 0.08 mm size, C31H25F3O5, *M* = 534.51, Orthorhombic, $P2_12_12_1$, *a* = 9.3677(5), *b* = 14.3667(8), *c* = 18.4604(13) Å, V = 2484.5(3)

Å^3, Z = 4, ρ_{calcd} = 1.429 gcm^{-3}, θmax = 30.79, Mo Kα, λ = 0.71073 Å, ϕ/ω-scan, Oxford Diffraction SuperNova diffractometer, T = 120(2) K, 49454 reflections collected of which 7278 were independent (R_{int} = 0.0764), direct primary solution and refinement on F^2 (SHELXS-97 and SHELXL-2014, G.M. Sheldrick, University of Göttingen, 2014), 380 refined parameters, CF_3 gorup disordered over two positions and refined with both similarity and U value component restrains, the absolute structure was not determined, $R_1[I > 2\sigma(I)]$ = 0.0522, wR_2(all data) = 0.1203, $\Delta\rho_{max}$ = 0.263 eÅ^{-3}. CCDC 1006013 contains the supplementary crystallographic data for this paper. These data can be obtained free of charge from The Cambridge Crystallographic Data Centre via www.ccdc.cam.ac.uk/data_request/cif.

Crystal packing for **6a**

In the crystal of compound **6a**, stronger interactions are of the type C-H···F, involving the CF_3 group, and C-H···O, involving the vinyl moiety (see table and figure). These intermolecular interactions could contribute to the preferred anti_s-trans conformation in the crystal.

Specified hydrogen bonds (with esds except riding H)

D-H	H...A	D...A	<(DHA)	H-BOND
0.95	2.38	3.241(4)	150.7	C3-H3B...O1_$1 ($1: x-1/2, -y+3/2, -z+2)
1.00	2.63	3.597(12)	163.4	C10-H10...F1_a$2 ($2: -x+1, y+1/2, -z+3/2)

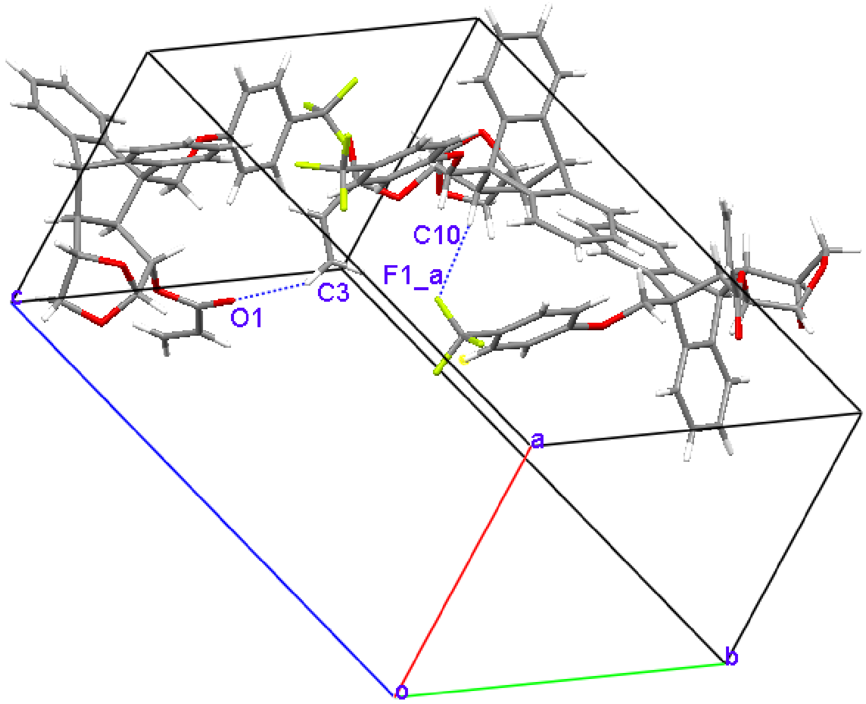

General procedure for the cycloaddition reaction of 6a and cyclopentadiene

Similar as described in [2], acrylic ester **6a** (26.7 mg, 0.05 mmol) was dissolved in the corresponding solvent (2.5 mL). When the reaction was promoted by a Lewis acid, 2 equiv of Et_2AlCl were added under nitrogen and the mixture was stirred at the corresponding temperature. Freshly distilled cyclopentadiene (41 μL, 0.5 mmol) was added dropwise and the mixture was stirred at the temperature and time indicated in Table S1. The cycloaddition reactions carried out without Lewis acid were concentrated after completion to afford a solid residue. The reactions promoted by Lewis acids were quenched by the addition of water (10 mL) and HCl (0.1 N, 10 mL), then extracted with CH_2Cl_2 (4 × 20 mL). The combined organic extracts were dried (Na_2SO_4) and concentrated. The solid residue was purified by flash chromatography to separate the excess of cyclopentadiene and the mixture of adducts **11a–14a** were collected together to obtain an accurate yield. *Endo*/*exo* and *endo R*/*S* ratios were determined by HPLC as shown in Figure S1.

Table S1: Diels–Alder reactions between acrylate **6a** and cyclopentadiene.

Entry	Lewis Acid	Solv.	T (ºC)	t (h)	Yield (%)	*endo*/*exo*	*endo R*/*S*	d.e. (*endo*)
1	-	PhMe	110	1.5	100	72 : 28	17 : 83	66%
2	-	PhMe	25	96	100	75 : 25	12 : 88	76%
3	-	CH_2Cl_2	25	144	85	80 : 20	12 : 88	76%
4	Et_2AlCl	CH_2Cl_2	0	0.5	75	92 : 8	92 : 8	84%
5	Et_2AlCl	CH_2Cl_2	- 40	1	63	94 : 6	94 : 6	88%
6	Et_2AlCl	CH_2Cl_2	-80	2	56	95 : 5	95 : 5	90%

Figure S1: HPLC Chromatogram of the mixtures of adducts **11a–14a**.

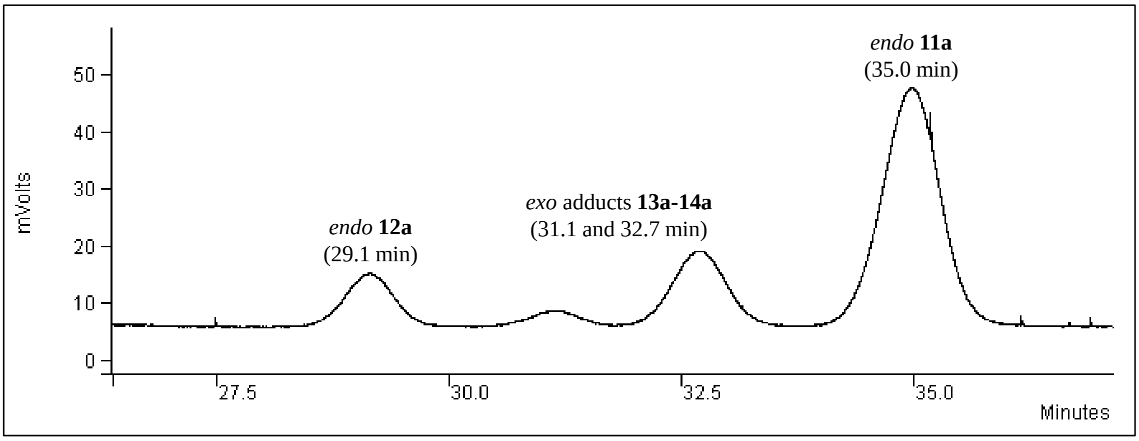

Conditions: MeCN/H_2O 70:30, 1.5 mL/min.

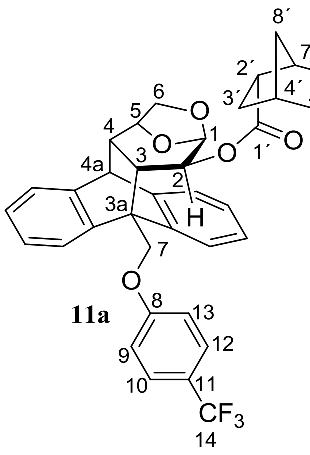

11a

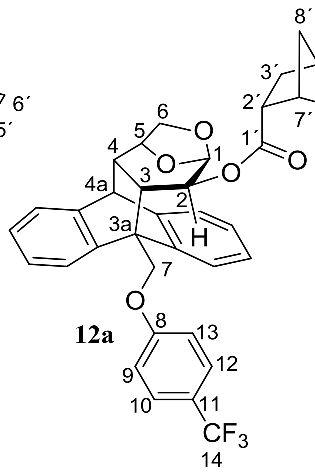

12a

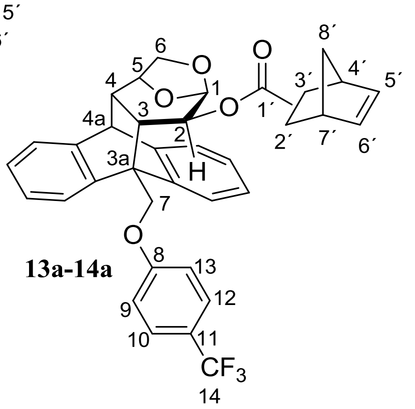

13a-14a

A second purification by flash chromatography can provide a mixture of *endo* **11a** and *exo* **13a** in the less polar fraction and a mixture of adducts *endo* **12a** and *exo* **14a** in the more polar fraction.

Less polar fraction, 11a and 13a: Colorless oil; IR (film) 3065, 2966, 1727 (C=O), 1590, 1332, 1256, 1161, 1111, 837, 747 cm^{-1}; ^{1}H NMR (300 MHz, $CDCl_3$) δ 7.65-7.62 (m, 2 H, H-10 and H-12 of **11a**), 7.61-7.58 (m, 2 H, H-10 and H-12 of **13a**), 7.43-7.32 (m, 4 H, aromatics of **1** y **2**), 7.28-7.11 (m, 16 H, 12 H aromatics of **11a** and **13a**, H-9 of **11a** and **13a**, H-13 of **11a** and **13a**), 6.03-5.97 (m, 2 H, H-5′ of **11a** and **13a**), 5.88 (dd, $J_{5'-6'}$= 5.4 Hz, $J_{6'-7'}$=2.8 Hz, 1 H, H-6′of **13a**), 5.83 (dd, $J_{5'-6'}$= 5.3 Hz, $J_{6'-7'}$=2.5 Hz, 1 H, H-6′of **11a**), 5.10-5.01 (m, 3H, H-7 of **11a** and **13a**, H-1 of **13a**), 4.93 (d, J_{1-2}= 3.2 Hz, 1H, H-1 of **11a**), 4.77-4.65 (m, 4 H, H-7 of **11a** and **13a**, H-5 of **11a** and **13a**), 4.35 (dd, J_{2-3}= 6.2 Hz, J_{1-2}= 3.3 Hz, 1 H, H-2 of **13a**), 4.25-4.22 (m, 3 H, H-2 of **11a**, H-4a of **11a** and **13a**), 3.75-3.73 (m, 4H, H-6 of **11a** and **13a**), 2.93-2.82 (m, 4 H, H-7′of **11a** and **13a**, H-3 of **11a** and **13a**), 2.72 (bs, 2 H, H-4′of **11a** and **13a**), 2.28-2.22 (m, 2 H, H-4 of **11a** and **13a**), 1.85 (bs, 1 H, H-2′of **11a**), 1.64-1.10 (m, 7 H, H-2′of **13a**, H-3′of **11a** and **13a**, H-3′of **11a**, H-8′of **11a** and **13a**, H-8′of **13a**), 0.92 (d, J_{gem}=7.9 Hz, 1 H, H-8′of **11a**), 0.70 (bs, 1 H, H-3′of **13a**); ^{13}C NMR (75.5 MHz, $CDCl_3$) δ 175.4 (C, C-1′of **13a**), 173.9 (C, C-1′of **11a**), 161.0 (C, C-8 of **11a**), 160.8 (C, C-8 of **13a**), 145.9 (C, aromatic of **11a**), 145.8 (C, aromatic of **13a**), 141.0 (C, 2 C, aromatic of **11a** and **13a**), 140.3 (C, 2 C, aromatic of **11a** and **13a**), 140.1 (C, 2 C, aromatic of **11a** and **13a**), 137.8 (CH, C-5′of **13a**), 137.5 (CH, C-5′of **11a**), 135.2 (CH, C-6′of **13a**), 131.8 (CH, C-6′of **11a**), 126.9 (CH, J_{C-F}= 3.2 Hz, 4 C, C-10 and C-12 of **11a** and **13a**), 126.5 (CH, 2 C, aromatic of **11a** and **13a**), 126.0 (CH, 4 C, aromatic of **11a** and **13a**), 125.8 (CH, 2 C, aromatic of **11a** and **13a**), 125.2 (CH, 2 C, aromatic of **11a** and **13a**), 124.2 (C, J_{C-F} =271,2 Hz, 2 C, C-14 of **11a** and **13a**), 123.7 (CH, 2 C, aromatic of **11a** and **13a**), 123.3 (C, J_{C-F} = 32,8 Hz, 2 C, C-11 of **11a** and **13a**), 122.1 (CH, 4 C, aromatic of **11a** and **13a**), 114.5 (CH, 2 C, C-9 and C-13 of **11a**), 114.3 (CH, 2 C, C-9 and C-13 of **13a**), 96.9 (CH, 2 C, C-1 of **11a** and **13a**), 76.5 (CH, 2 C, C-5 of **11a** and **13a**), 70.3 (CH_2, 2 C, C-6 of **11a** and **13a**), 70.0 (CH, C-2 of **13a**), 69.9 (CH, C-2 of **11a**), 66.1 (CH_2, 2 C, C-7 of **11a** and **13a**), 50.5 (CH, 2 C, C-4a of **11a** and **13a**), 49.5 (C, 2 C, C-3a of **11a** and **13a**), 49.1 (CH_2, C-8′of **11a**), 47.3 (CH, 2 C, C-4 of **11a** and **13a**), 46.9 (CH, C-7′of **13a**), 45.6 (CH, C-7′of **11a** and CH_2, C-8′of **13a**), 42.8 (CH, C-2′of **13a**), 42.5 (CH, C-2′of **11a**), 42.3 (CH, C-4′ of **11a**), 41.4 (CH, C-4′of **13a**), 36.2 (CH, 2 C, C-3 of **11a** and **13a**), 29.4 (CH_2, C-3′of **13a**), 28.5 (CH_2, C-3′of **11a**); ^{19}F NMR (282.4 MHz, $CDCl_3$) δ - 61.4 and -61.4.

More polar fraction: 12a and 14a: Colorless oil; IR (film) 3066, 2929, 1728 (C=O), 1590, 1331, 1256, 1112, 838, 748 cm^{-1}; ^{1}H NMR (300 MHz, $CDCl_3$) δ 7.67-7.63 (m, 4 H, H-10 and H-12 of **12a** and **14a**), 7.44-7.11 (m, 20 H, 16 H aromatics of **12a** and **14a,** H-9 of **12a** and **14a**, H-13 of **12a** and **14a**), 6.06 (m, 1 H, H-5′ of **12a**), 5.99 (dd, $J_{5'-6'}$=5.3 Hz, $J_{5'-4'}$=2.8 Hz, 1 H, H-5′of **14a**),5.91-5.89 (m, 1 H, H-6′of **12a**), 5.70 (bs, 1 H, H-6′of **14a**), 5.08-5.01 (m, 3 H, H-7 of **12a** and **14a**, H-1 of **14a**), 4.93 (d, J_{1-2}= 3.2 Hz, 1 H, H-1 of **12a**), 4.83-4.67 (m, 4 H, H-7 of **12a** and **14a**, H-5 of **12a** and **14a**), 4.38 (dd, J_{2-3}= 6.2 Hz, J_{1-2}= 3.4 Hz, 1 H, H-2 of **14a**), 4.30-4.24 (m, 3 H, H-2 of **12a**, H-4a of **12a** and **14a**), 3.77-3.71 (m, 4H, H-6 of **12a** and **14a**), 2.76 (bs, 5 H, H-7′of **12a**, H-3 of **12a** and **14a,** H-4′of **12a** and **14a**), 2.53 (bs, H-7′of **14a**), 2.31-2.24 (m, 3H, H-4 of **12a** and **14a**, H-2′of **12a**), 1.73-1.66 (m, 3 H, H-2′of **14a**, H-3′ of **12a** and **14a**), 1.37-1.13 (m, 5 H, H-8′of **14a**, H-8′ of **12a** and **14a**, H-3′ of **12a** and **14a**), 0.96 (d, J_{gem}= 7.7 Hz, 1 H, H-8′de **12a**).; ^{13}C NMR (75.5 MHz, $CDCl_3$) δ 175.9 (C, C-1′of **14a**), 174.2 (C, C-1′of **12a**), 160.9 (C, C-8 of **12a**), 160.8 (C, C-8 of **14a**), 145.8 (C, 2 C, aromatic of **12a** and **14a**), 141.0 (C, 2 C, aromatic of **12a** and **14a**), 140.2 (C, 2 C, aromatic of **12a** and **14a**), 140.1 (C, 2 C, aromatic of **12a** and **14a**), 137.7 (CH, C-5′of **14a**), 137.3 (CH, C-5′of **12a**), 135.2 (CH, C-6′of **14a**), 132.8 (CH, C-6′of **12a**), 127.0 (CH, J_{C-F}= 3.2 Hz, 4 C, C-10 and C-12 of **12a** and **14a**), 126.6 (CH, 2 C, aromatic of **12a** and **14a**), 126.1 (CH, 4 C, aromatic of **12a** and **14a**), 125.9 (CH, 2 C, aromatic of **12a** and **14a**), 125.2 (CH, 2C, aromatic of **12a** and **14a**), 124.2 (C, J_{C-F} =271,5 Hz, 2 C, C-14 of **12a** and **14a**), 123.7 (CH, 2 C, aromatic of **12a** and **14a**), 123.5 (C, J_{C-F} = 32.9 Hz, 2 C, C-11 of **12a** and **14a**), 122.5 (CH, 2 C, aromatic of **12a** and **14a**), 122.2 (CH, 2 C, aromatic of **12a** and **14a**), 114.6 (CH, 2 C, C-9 and C-13 of **12a**), 114.4 (CH, 2 C, C-9 and C-13 of **14a**), 97.0 (CH, 2 C, C-1 of **12a** and **14a**), 77.1 (CH, 2 C, C-5 of **12a** and **14a**), 70.4 (CH_2, C-6 of **14a**), 70.3 (CH_2, C-6 of **12a**), 70.0 (CH, 2 C, C-2 of **12a** and **14a**), 66.2 (CH_2, C-7 of **12a**), 66.0 (CH_2, C-7 of **14a**), 50.6 (CH, 2 C, C-4a of **12a** and **14a**), 49.7 (C, 2 C, C-3a of **12a** and **14a**), 49.1 (CH_2, C-8′of **12a**), 47.4 (CH, 2 C, C-4 of **12a** and **14a**), 46.4 (CH_2, C-8′of **14a**), 45.6 (CH, C-7′of **14a**), 44.8 (CH, C-7′of **12a**), 43.4 (CH, C-2′of **12a**), 42.4 (CH, C-2′of **14a**), 42.2 (CH, C-4′ of **12a**), 41.3 (CH, C-4′of **14a**), 36.6 (CH, 2C, C-3 of **12a** and **14a**), 31.0 (CH_2, C-3′of **14a**), 30.2 (CH_2, C-3′of **12a**); ^{19}F NMR (282.4 MHz, $CDCl_3$) δ - 61.5 and -61.4.

9-[(4-Methoxyphenoxy)methyl]anthracene (2b)

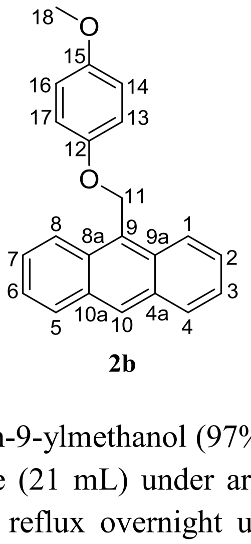

2b

Analogous as described in [2], anthracen-9-ylmethanol (97%, 3360 mg, 15.65 mmol) was dissolved in dry CH_2Cl_2 (7 mL) and dry benzene (21 mL) under argon. $SOCl_2$ was added (1.5 mL, 20.56 mmol) and the solution was stirred at reflux overnight under argon. The reaction mixture was poured into ice–water (50 mL). The organic phase was washed with water (3 × 35 mL), dried

(Na_2SO_4) and concentrated. The residual solid was dissolved in dry acetone (80 mL). 4-Methoxyphenol (99%, 2999 mg, 23.92 mmol) and anhydrous K_2CO_3 (99%, 3300 mg, 23.64 mmol) were incorporated and the solution was stirred at reflux for 22 h under argon. The solvent was evaporated under reduced pressure and water (150 mL) and CH_2Cl_2 (150 mL) were added to the residue. The aqueous phase was extracted with CH_2Cl_2 (3 × 100 mL). The organic phase was washed with saturated Na_2CO_3 (2 × 150 mL), brine (2 × 150 mL), dried (Na_2SO_4) and concentrated. The residual solid was purified by flash chromatography to afford **2b** (3976 mg, 12.65 mmol, 81%).

2b: Colorless crystalline solid; mp 123-124 ºC (Hexane-CH_2Cl_2); IR (KBr) 3062, 2998, 1590, 1507, 1222, 1009, 829 cm^{-1}; ^{1}H NMR (300 MHz, $CDCl_3$) δ 8.52 (s, 1 H, H-10), 8.31 (d, $J_{1\text{-}2}$= $J_{7\text{-}8}$= 8.6 Hz, 2 H, H-1 and H-8), 8.05 (dd, $J_{3\text{-}4}$= $J_{5\text{-}6}$= 7.9 Hz, $J_{2\text{-}4}$= $J_{5\text{-}7}$= 1.4 Hz, 2 H, H-4 and H-5), 7.57-7.46 (m, 4 H, H-2 , H-3, H-6 and H-7), 7.13-7.07 (m, 2 H, H-13 and H-17), 6.96-6.90 (m, 2 H, H-14 and H-16), 5.91 (s, 2 H, H-11), 3.82 (s, 3 H , H-18); ^{13}C NMR (75.5 MHz, $CDCl_3$) δ 154.0 (C, C-15), 153.3 (C, C-12), 131.3 (C, 2 C, C-4a and C-10a), 130.8 (C, 2 C, C-8a and C-9a), 128.9 (CH, 2 C, C-4 and C-5), 128.7 (CH, C-10), 127.0 (C, C-9), 126.3 (CH, 2 C, C-2 and C-7)*, 124.8 (CH, 2 C, C-3 and C-6)*, 123.9 (CH, 2 C, C-1 and C-8), 115.7 (CH, 2 C, C-13 and C-17), 114.6 (CH, 2 C, C-14 and C-16) , 63.1 (CH_2, C-11), 55.5 (CH_3, C-18); HRMS calc. for $C_{22}H_{18}O_2Na$ $[M+Na]^+$ 337.1199. Found 337.1191.

Ketone 3b

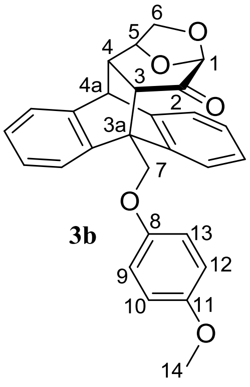

3b

Method 1: Thermal conditions

In a similar manner as described in [2], levoglucosenone (262 mg, 2.08 mmol) and **2b** (917 mg, 2.92 mmol) were dissolved in toluene (8.5 mL) at rt and the obtained solution was heated under reflux for 8 d. The solvent was evaporated under vacuum and the solid residue was purified by flash chromatography to give **3b** (782 mg, 1.78 mmol, 86%).

Method 2: Microwave conditions

Levoglucosenone (82 mg, 0.65 mmol) and **2b** (410 mg, 1.30 mmol) were placed in a 10 mL vial and dissolved in THF (0.28 mL) and the solution was heated at 150 °C for 4 h. The solvent was evaporated under reduced pressure and the residue was purified by flash chromatography to give **3b** (217 mg, 0.49 mmol, 76%).

3b: Yellow oil ; $[\alpha]^{26}_{D}$ -46.1 (*c* 1.10, $CHCl_3$); IR (film) 2957, 2899, 1719 (C=O), 1508, 1230, 731 cm^{-1} ; ^{1}H NMR (300 MHz, $CDCl_3$) δ 7.49-7.41 (m, 2 H, aromatics), 7.30-7.10 (m, 8 H, 6 H aromatics, H-9 and H-13), 6.96-6.90 (m, 2 H, H-10 and H-12), 5.50 (d, J_{gem}= 9.1 Hz, 1 H, H-7), 5.16 (d, J_{gem}= 9.1 Hz, 1 H, H-7), 4.82 (d, $J_{5\text{-}6exo}$= 4.5 Hz, 1 H, H-5), 4.57 (s, 1 H, H-1), 4.34 (s, 1 H, H-4a), 3.81 (s, 1 H, H-14), 3.79-3.74 (m, 1 H, H-6*exo*), 3.70-3.68 (m, 1 H, H-6*endo*), 3.24 (d, $J_{3\text{-}4}$= 9.7 Hz, 1 H, H-3), 2.35 (d, $J_{3\text{-}4}$= 9.7 Hz, 1 H, H-4); ^{13}C NMR (75.5 MHz, $CDCl_3$) δ 198.8 (C, C-2), 154.1 (C, C-11), 152.8 (C, C-8), 145.1 (C, aromatic), 140.9 (C, aromatic), 140.7 (C, aromatic), 140.1 (C, aromatic), 126.3 (CH, aromatic), 126.1 (CH, aromatic), 126.0 (CH, aromatic), 125.9 (CH, aromatic), 125.1 (CH, aromatic), 124.2 (CH, aromatic), 122.2 (CH, aromatic), 121.5 (CH, aromatic), 115.8 (CH, 2 C, C-9 and C-13), 114.6 (CH, 2 C, C-10 and C-12), 99.7 (CH, C-1), 77.0 (CH, C-5), 68.8 (CH_2, C-6), 66.9 (CH_2, C-7), 55.6 (CH_3, C-14), 50.2 (CH, C-4a), 49.6 (C, C-3a), 44.6 (CH, C-4), 44.2 (CH, C-3); HRMS calc. for $C_{28}H_{25}O_5$ $[M+H]^+$ 441.1697. Found 441.1686.

Reduction of ketone 3b: alcohols 4b and 5b

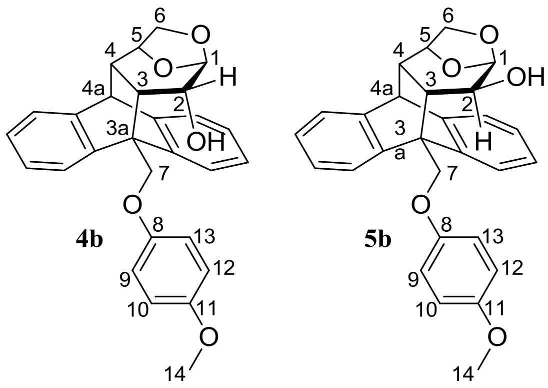

Analogous as described in [2], ketone **3b** (1098 mg, 2.49 mmol) was dissolved in a CH_2Cl_2/MeOH 97:3 mixture (50 mL) at room temperature and $NaBH_4$ (97 mg, 2.56 mmol) added. The mixture was stirred for 24 h and then acetone (10 mL) was added. The solution was filtered through a short pad of Celite and the residue was purified by flash chromatography to afford **4b** (395 mg, 0.89 mmol, 36%) and **5b** (639 mg, 1.44 mmol, 58%).

4b: Colorless cristalline solid; mp 199-200 ºC (hexane/AcOEt); $[\alpha]_{D}^{27}$ = +65.3 (*c* 0.97, $CHCl_3$); IR (KBr) 3567 (OH), 3020, 2950, 1511, 1231, 825, 760 cm^{-1}; ^{1}H NMR (300 MHz, $CDCl_3$) δ 7.46-7.37 (m, 2 H, aromatics), 7.33-7.28 (m, 1 H, aromatic), 7.23-7.04 (m, 7 H, 5 H aromatics, H-9 and H-13), 6.96-6.90 (m, 2 H, H-10 and H-12), 5.43 (d, J_{gem}=9.1 Hz, 1 H, H-7), 5.24 (d, J_{gem}=9.1 Hz, 1 H, H-7), 4.98 (d, $J_{1\text{-}2}$=1.2 Hz, 1 H, H-1), 4.67 (d, $J_{5\text{-}6}$= 3.8 Hz, 1 H, H-5), 4.17 (d, $J_{4\text{-}4a}$=1.1 Hz, 1 H, H-4a), 3.89 (ddd, $J_{2\text{-}OH}$=13.4 Hz, $J_{2\text{-}3}$=7.9 Hz, $J_{1\text{-}2}$=1.2 Hz, 1 H, H-2), 3.81 (s, 3 H, H-14), 3.75-3.67 (m, 2 H, H-6), 2.92 (dd, $J_{3\text{-}4}$=10.9 Hz, $J_{2\text{-}3}$=7.9 Hz, 1 H, H-3), 2.06 (d, $J_{3\text{-}4}$=10.9 Hz, 1 H, H-4), 0.69 (d, $J_{2\text{-}OH}$=13.4 Hz, 1 H, OH); ^{13}C NMR (75.5 MHz, $CDCl_3$) δ 154.0 (C, C-11), 153.0 (C, C-8), 147.1 (C, aromatic), 143.6 (C, aromatic), 142.0 (C, aromatic), 141.0 (C, aromatic), 125.8 (CH, aromatic), 125.7 (CH, aromatic), 125.7 (CH, aromatic), 125.6 (CH, aromatic), 125.1 (CH, aromatic), 123.5 (CH, aromatic), 122.9 (CH, aromatic), 121.8 (CH, aromatic), 115.5 (CH, 2 C, C-9 and C-13), 114.7 (CH, 2 C, C-10 and C-12), 102.2 (CH, C-1), 76.2 (CH, C-5), 72.1 (CH_2, C-6),

68.1 (CH, C-2), 66.9 (CH_2, C-7), 55.7 (CH_3, C-14), 50.7 (CH, C-4a), 49.6 (C, C-3a), 45.1 (CH, C-4), 34.3 (CH, C-3); HRMS calc. for $C_{28}H_{30}O_5N$ $[M+NH_4]^+$ 460.2119. Found 460.2106.

5b: yellow oil; $[\alpha]_D^{26}$ = -2.6 (*c* 1.02, $CHCl_3$); IR (film) 3460 (OH), 3019, 2952, 1506, 1233, 824, 751 cm^{-1}; ^1H NMR (300 MHz, $CDCl_3$) δ 7.48-7.36 (m, 2 H, aromatics), 7.30-7.07 (m, 8 H, 6 H aromatics, H-9 and H-13), 6.95-6.89 (m, 2 H, H-10 and H-12), 5.09 (d, J_{gem}=9.8 Hz, 1 H, H-7), 4.99-4.96 (m, 2 H, H-1, H-7), 4.68 (d, $J_{5\text{-}6exo}$= 4.9 Hz, 1 H, H-5), 4.19 (s, 1 H, H-4a), 3.80 (s, 1 H, H-14), 3.74 (dd, J_{gem}=7.1 Hz, $J_{5\text{-}6exo}$=4.9 Hz, 1 H, H-6*exo*), 3.64 (d, J_{gem}=7.1 Hz, H-6*endo*), 2.97 (ddd, $J_{2\text{-}OH}$=10.2 Hz, $J_{2\text{-}3}$=5.9 Hz, $J_{1\text{-}2}$=3.6 Hz, 1 H, H-2), 2.28 (dd, $J_{3\text{-}4}$=10.2 Hz, $J_{2\text{-}3}$=5.9 Hz, 1 H, H-3), 2.11 (d, $J_{3\text{-}4}$=10.2 Hz, 1 H, H-4), 2.02 (d, $J_{2\text{-}OH}$=10.2 Hz, 1 H, OH); ^{13}C NMR (75.5 MHz, $CDCl_3$) δ 153.9 (C, C-11), 152.5 (C, C-8), 146.0 (C, aromatic), 141.1 (C, aromatic), 140.9 (C, aromatic), 140.3 (C, aromatic), 126.0 (CH, aromatic), 125.6 (CH, 2 C, aromatics), 125.4 (CH, aromatic), 124.8 (CH, aromatic), 123.8 (CH, aromatic), 123.2 (CH, aromatic), 121.7 (CH, aromatic), 115.4 (CH, 2 C, C-9 and C-13), 114.6 (CH, 2 C, C-10 and C-12), 99.7 (CH, C-1), 76.2 (CH, C-5), 69.9 (CH_2, C-6), 68.5 (CH, C-2), 67.2 (CH_2, C-7), 55.4 (CH_3, C-14), 50.1 (C, C-3a), 50.0 (CH, C-4a), 47.3 (CH, C-4), 41.6 (CH, C-3); HRMS calc. for $C_{28}H_{26}O_5Na$ $[M+Na]^+$ 465.1673. Found 465.1658.

Oxidation of alcohol 4b

Similar as described in [2], alcohol **4b** (243 mg, 0.55 mmol) was dissolved in dry CH_2Cl_2 (12 mL) and PCC (372 mg, 1.73 mmol) was added in one portion and the reaction mixture was stirred overnight under argon atmosphere. Afterwards the mixture was diluted with CH_2Cl_2 and filtered through a sintered glass funnel containing Florisil®. The filtrate was concentrated to give pure ketone **3b** (176 mg, 0.40 mmol, 73%).

Preparation of acrylate 6b

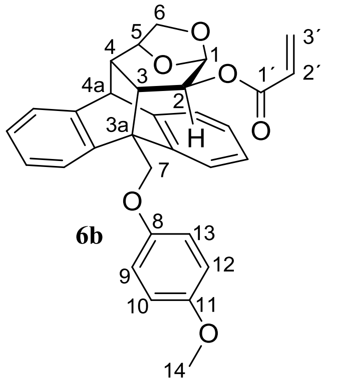

6b

Analogous as described in [2], alcohol **5b** (540mg, 1.22 mmol) was dissolved in dry CH_2Cl_2 (28 mL) and cooled to 0 °C. Dry triethylamine (700 µL, 5.03 mmol) and acryloyl chloride (231 µL, 2.84 mmol) were added and the mixture was stirred for 1 h under argon atmosphere. Then a few drops of water were added, the solution dried (Na_2SO_4) and concentrated. The residue was purified by flash chromatography to give **6b** (394 mg, 0.79 mmol, 65%).

6b: Colorless crystalline solid; mp 237-238 ºC (Hexane-CH_2Cl_2); $[\alpha]_D^{25}$ = -11.7 (*c* 1.02, $CHCl_3$); IR (KBr) 3059, 2940, 1711 (C=O), 1513, 1053, 831, 746 cm^{-1}; ^1H NMR (300 MHz, $CDCl_3$) δ 7.44-

7.39 (m, 2 H, aromatics), 7.28-7.18 (m, 4 H, aromatics), 7.14-7.08 (m, 2 H, aromatics), 6.97-6.92 (m, 2 H, H-9 and H-13), 6.88-6.83 (m, 2 H, H-10 and H-12), 6.15 (d, J_{vec}= 16.7 Hz, 1 H, H-3′*cis*), 5.68 (dd, J_{vec}= 16.7 Hz, J_{vec}= 10.3 Hz, 1 H, H-2′), 5.49 (d, J_{vec}= 10.3 Hz, 1 H, H-3′*trans*), 5.11 (d, $J_{1\text{-}2}$= 3.3 Hz, 1 H, H-1), 4.93 (d, J_{gem}= 9.7 Hz, 1 H, H-7), 4.69 (m, 1 H, H-5), 4.61 (d, J_{gem}= 9.7 Hz, 1 H, H-7), 4.36 (dd, $J_{2\text{-}3}$= 6.4 Hz, $J_{1\text{-}2}$= 3.3 Hz, 1 H, H-2), 4.23 (d, $J_{4\text{-}4a}$= 0.8 Hz, 1 H, H-4a), 3.78 (s, 3 H, H-14), 3.75-3.74 (m, 2 H, H-6), 2.88 (m,1 H, H-3), 2.26 (d, $J_{3\text{-}4}$= 10.6 Hz, 1 H, H-4); ^{13}C NMR (75.5 MHz, $CDCl_3$) δ 165.3 (C, C-1′), 153.9 (C, C-11), 152.5 (C, C-8), 145.9 (C, aromatic), 141.0 (C, aromatic), 140.6 (C, aromatic), 140.4 (C, aromatic), 130.7 (CH_2, C-3′), 127.0 (CH, C-2′), 126.4 (CH, aromatic), 126.0 (CH, aromatic), 125.8 (CH, aromatic), 125.8 (CH, aromatic), 125.0 (CH, aromatic), 124.0 (CH, aromatic), 122.5 (CH, aromatic), 121.9 (CH, aromatic), 114.8 (CH, 2 C, C-9 and C-13), 114.5 (CH, 2 C, C-10 and C-12), 97.0 (CH, C-1), 76.5 (CH, C-5), 70.5 (CH, C-2), 70.3 (CH_2, C-6), 65.7 (CH_2, C-7), 55.6 (CH_3, C-14), 50.4 (CH, C-4a), 49.6 (C, C-3a), 47.4 (CH, C-4), 36.3 (CH, C-3); HRMS calc. for $C_{31}H_{28}O_6Na$ $[M+Na]^+$ 519.1778. Found 519.1768.

General procedure for the cycloaddition reaction of 6b and cyclopentadiene

Similar as described in [2], acrylic ester ester **6b** (24.8 mg, 0.05 mmol) was dissolved in the corresponding solvent (2.5 mL). When the reaction was promoted by a Lewis acid, 2 equiv of Et_2AlCl were added under nitrogen and the mixture was stirred at the corresponding temperature. Freshly distilled cyclopentadiene (41 μL, 0.5 mmol) was added dropwise and the mixture was stirred at the temperature and time indicated in Table S2. The cycloaddition reactions carried out without Lewis acid were concentrated after completion to afford a solid residue. The reactions promoted by Lewis acids were quenched by the addition of water (10 mL) and HCl (0.1 N, 10 mL), then extracted with CH_2Cl_2 (4 × 20 mL). The combined organic extracts were dried (Na_2SO_4) and concentrated. The solid residue was purified by flash chromatography to separate the excess of cyclopentadiene and the mixture of adducts **11b–14b** were collected together to obtain an accurate yield. *Endo/exo* and *endo R/S* ratios were determined by HPLC as shown in Figure S2.

Table S2: Diels-Alder reactions between acrylate **6b** and cyclopentadiene

Entry	Lewis Acid	Solv.	T (ºC)	t (h)	Yield (%)	*endo/exo*	*endo R/S*	d.e. (*endo*)
1	-	PhMe	110	1.5	93	69 : 31	21 : 79	58%
2	-	PhMe	25	72	93	73 : 27	16 : 84	68%
3	-	CH_2Cl_2	25	96	92	78 : 22	18 : 82	64%
4	Et_2AlCl	CH_2Cl_2	0	0.5	80	92 : 8	94 : 6	88%
5	Et_2AlCl	CH_2Cl_2	- 40	1	87	93 : 7	95 : 5	90%

Figure S2: HPLC Chromatogram of the mixtures of adducts **11b-14b**

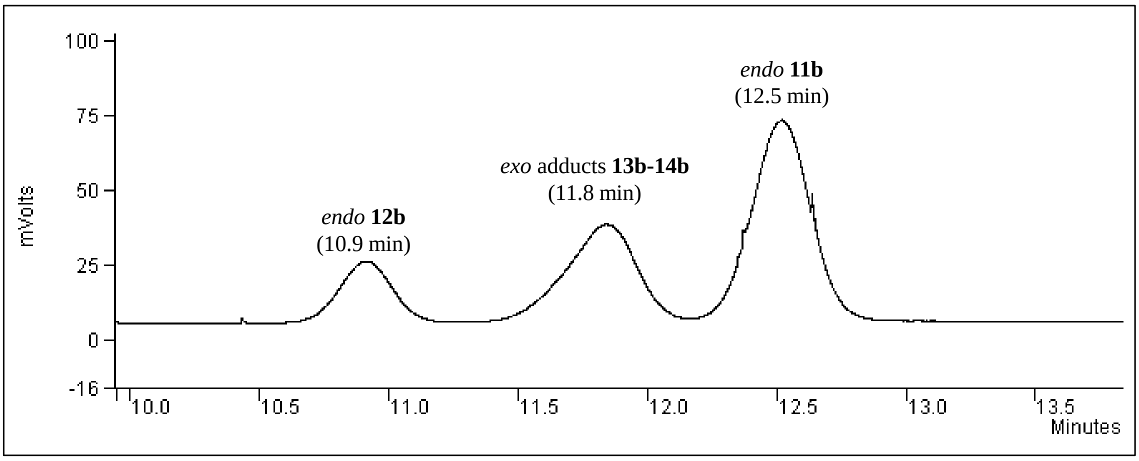

Conditions: MeCN-H_2O 80:20, 1 mL/min.

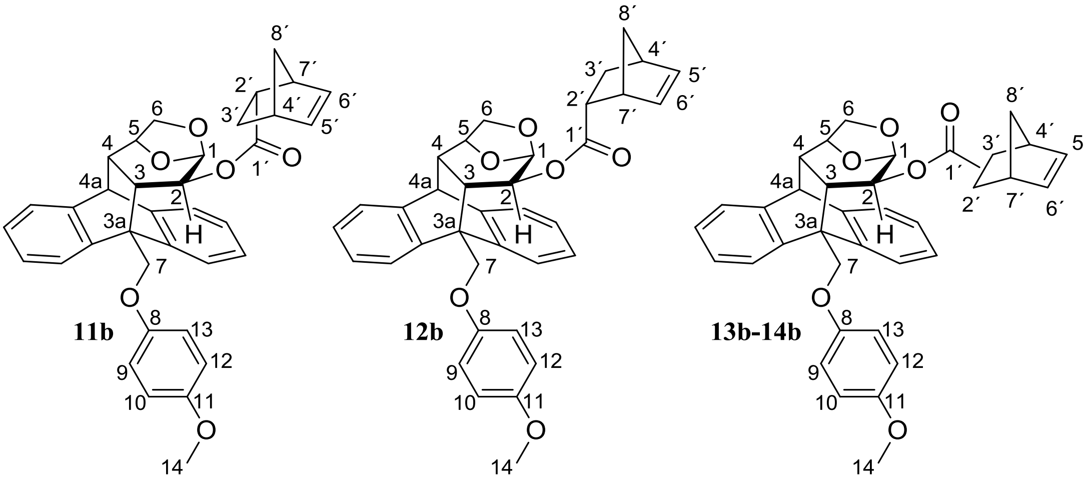

A second purification by flash chromatography allows the isolation of a mixture of adducts *endo* **11b** and *exo* **13b** in the less polar fraction, a mixture of adducts *endo* **12b** and *exo* **14b** in the fraction of intermediate polarity and pure *endo* **12b** in the more polar fraction.

Less polar fraction. 11b and 13b: White solid; IR (KBr) 3065, 2953, 1726 (C=O), 1508, 1236, 826, 748 cm^{-1}; ^{1}H NMR (300 MHz, $CDCl_3$) δ 7.46-7.35 (m, 4 H, aromatics of **11b** and **13b**), 7.28-7.08 (m, 12 H, aromatics of **11b** and **13b**), 7.04-6.86 (m, 8 H, H-9 of **11b** and **13b**, H-10 of **11b** and **13b**, H-12 of **11b** and **13b**, H-13 of **11b** and **13b**), 6.04-5.99 (m, 2 H, H-5′ of **11b** and **13b**), 5.94 (dd, $J_{5'\text{-}6'}$= 5.4 Hz, $J_{6'\text{-}7'}$=3.0 Hz, 1 H, H-6′of **13b**), 5.85 (dd, $J_{5'\text{-}6'}$= 5.6 Hz, $J_{6'\text{-}7'}$=2.7 Hz, 1 H, H-6′of **11b**), 5.03 (d, $J_{1\text{-}2}$= 3.3 Hz, 1H, H-1 of **13b**), 4.99-4.92 (m, 3 H, H-7 of **11b** and **13b**, H-1 of **11b**), 4.70-4.64 (m, 3 H, H-7 of **13b**, H-5 of **11b** and **13b**), 4.58 (d, J_{gem}= 9.7 Hz, 1 H, H-7 of **11b**), 4.35 (dd, $J_{2\text{-}3}$= 6.2 Hz, $J_{1\text{-}2}$= 3.3 Hz, 1 H, H-2 of **13b**), 4.24-4.20 (m, 3 H, H-2 of **11b**, H-4a of **11b** and **13b**), 3.80 (s, 3 H, H-14 of **11b**), 3.78 (s, 3 H, H-14 of **13b**), 3.77-3.72 (m, 4 H, H-6 of **11b** and **13b**), 2.99 (bs, 1 H, H-7′of **11b**), 2.87 (m, 3 H, H-3 of **11b** and **13b**, H-7′of **13b**), 2.74 (bs, 2 H, H-4′of **11b** and **13b**), 2.27-2.23 (m, 2 H, H-4 of **11b** and **13b**), 2.06 (bs, 1 H, H-2′of **11b**), 1.73-1.66 (m, 2 H, H-2′of **13b,** H-3′of **13b**), 1.46-1.15 (m, 5 H, H-3′of **11b**, H-8′of **11b** and **13b**, H-8′of **13b**, H-3′of **11b**), 0.97 (d, J_{gem}=8.0 Hz, 1 H, H-8′of **11b**), 0.85 (bs, 1 H, H-3′of **13b**); ^{13}C NMR (75.5 MHz, $CDCl_3$) δ 175.0 (C, C-1′of **13b**), 174.1 (C, C-1′of **11b**), 154.0 (C, 2 C, C-11 of **11b** and **13b**),

152.8 (C, C-8 of **11b**), 152.6 (C, C-8 of **13b**), 146.0 (C, aromatic of **11b**), 145.9 (C, aromatic of **13b**), 141.1 (C, 2 C, aromatic of **11b** and **13b**), 140.7 (C, aromatic of **11b**), 140.7 (C, aromatic of **13b**), 140.5 (C, 2 C, aromatic of **11b** and **13b**), 137.7 (CH, C-5′of **13b**), 137.4 (CH, C-5′of **11b**), 135.4 (CH, C-6′of **13b**), 132.0 (CH, C-6′of **11b**), 126.4 (CH, 2 C, aromatic of **11b** and **13b**), 126.0 (CH, aromatic of **13b**), 125.9 (CH, aromatic of **11b**), 125.8 (CH, 4 C, aromatic of **11b** and **13b**), 125.0 (CH, 2 C, aromatic of **11b** and **13b**), 124.1 (CH, 2 C, aromatic of **11b** and **13b**), 122.6 (CH, aromatic of **13b**), 122.5 (CH, aromatic of **11b**), 122.0 (CH, 2 C, aromatic of **11b** and **13b**), 115.0 (CH, 2C, C-9 and C-13 of **11b**), 114.9 (CH, 2 C, C-9 and C-13 of **13b**), 114.7 (CH, 2 C, C-10 and C-12 of **13b**), 114.7 (CH, 2 C, C-10 and C-12 of **11b**), 97.0 (CH, 2 C, C-1 of **11b** and **13b**), 76.6 (CH, 2 C, C-5 of **11b** and **13b**), 70.3 (CH_2, 2 C, C-6 of **11b** and **13b**), 70.0 (CH, C-2 of **13b**), 70.0 (CH, C-2 of **11b**), 65.9 (CH_2, 2 C, C-7 of **11b** and **13b**), 55.7 (CH_3, 2 C, C-14 of **11b** and **13b**), 50.5 (CH, 2 C, C-4a of **11b** and **13b**), 49.7 (C, 2 C, C-3a of **11b** and **13b**), 49.2 (CH_2, C-8′of **11b**), 47.4 (CH, 2 C, C-4 of **11b** and **13b**), 46.8 (CH, C-7′of **13b**), 45.8 (CH_2, C-8′of **13b**), 45.6 (CH, C-7′of **11b**), 42.8 (CH, C-2′of **13b**), 42.5 (CH, C-2′of **11b**), 42.3 (CH, C-4′ of **11b**), 41.4 (CH, C-4′of **13b**), 36.4 (CH, C-3 of **13b**), 36.1 (CH, C-3 of **1**), 29.7 (CH_2, C-3′of **13b**), 28.6 (CH_2, C-3′of **11b**).

Fraction of intermediate polarity. 12b and 14b: Colorless oil; IR (film) 3065, 2953, 1724 (C=O), 1506, 1234, 824, 747 cm^{-1}; ^{1}H NMR (300 MHz, $CDCl_3$) δ 7.45-7.37 (m, 4 H, aromatics of **12b** and **14b**), 7.28-7.16 (m, 8 H, aromatics of **12b** and **14b**), 7.14-7.08 (m, 4 H, aromatics of **12b** and **14b**), 7.07-6.99 (m, 4 H, H-9 of **12b** and **14b**, H-13 of **12b** and **14b**), 6.95-6.89 (m, 4 H, H-10 of **12b** and **14b**, H-12 de of **12b** and **14b**), 6.07 (dd, $J_{5'-6'}$=5.3 Hz, $J_{5'-4'}$=3.0 Hz, 1 H, H-5′of **12b**), 6.00 (dd, $J_{5'-6'}$=5.5 Hz, $J_{5'-4'}$=2.8 Hz, 1 H, H-5′of **14b**), 5.91 (dd, $J_{5'-6'}$= 5.3 Hz, $J_{6'-7'}$=2.7 Hz, 1 H, H-6′of **12b**), 5.77 (bs, 1 H, H-6′of **14b**), 5.02 (d, J_{1-2}=3.4 Hz, 1 H, H-1 of **14b**), 4.95-4.93 (m, 3 H, H-7 of **12b** and **14b**, H-1 of **12b**), 4.70-4.64 (m, 4 H, H-7 of **12b** and **14b**, H-5 of **12b** and **14b**), 4.37 (dd, J_{2-3}= 6.2 Hz, J_{1-2}= 3.4 Hz, 1 H, H-2 of **14b**), 4.26 (dd, J_{2-3}= 6.2 Hz, J_{1-2}= 3.4 Hz, 1 H, H-2 of **12b**), 4.21 (s, 2 H, H-4a of **12b** and **14b**), 3.80 (s, 3 H, H-14 of **14b**), 3.80 (s, 3 H, H-14 of **12b**), 3.75-3.67 (m, 4 H, H-6 of **12b** and **14b**), 2.81-2.76 (m, 5 H, H-7′of **12b**, H-3 of **12b** and **14b,** H-4′of **12b** and **14b**), 2.63 (bs, 1 H, H-7′of **14b**), 2.34 (bs, 1 H, H-2′of **12b**), 2.28-2.21 (m, 2 H, H-4 of **12b** and **14b**), 1.79-1.70 (m, 3 H, H-2′of **14b**, H-3′of **12b** and **14b**), 1.40 (d, J_{gem}=7.5 Hz, 1 H, H-8′of **14b**), 1.29-1.15 (m, 4 H, H-8′of **12b** and **14b**, H-3′of **12b** and **14b**), 1.00 (d, J_{gem}= 8.2 Hz, 1 H, H-8′of **12b**); ^{13}C NMR (75.5 MHz, $CDCl_3$) δ 175.9 (C, C-1′of **14b**), 174.3 (C, C-1′of **12b**), 154.1 (C, C-11 of **12b**), 154.0 (C, C-11 of **14b**), 152.8 (C, C-8 of **12b**), 152.6 (C, C-8 of **14b**), 145.9 (C, 2 C, aromatic of **12b** and **14b**), 141.0 (C, 2 C, aromatic of **12b** and **14b**), 140.6 (C, 2 C, aromatic of **12b** and **14b**), 140.5 (C, 2 C, aromatic of **12b** and **14b**), 137.6 (CH, C-5′of **14b**), 137.1 (CH, C-5′of **12b**), 135.6 (CH, C-6′of **14b**), 132.9 (CH, C-6′of **12b**), 126.4 (CH, 2 C, aromatic of **12b** and **14b**), 126.0 (CH, 2 C, aromatic of **12b** and **14b**), 125.9 (CH, 2 C, aromatic of **12b** and **14b**), 125.8 (CH, 2 C, aromatic of **12b** and **14b**), 125.0 (CH, 2 C, aromatic of **12b** and **14b**), 124.1 (CH, 2 C, aromatic of **12b** and **14b**), 122.8 (CH, 2 C, aromatic of **12b** and **14b**), 122.0 (CH, 2 C, aromatic of **12b** and **14b**), 115.1 (CH, 2 C, C-9 and C-13 of **12b**), 114.9 (CH, 2 C, C-9 and C-13 of **14b**), 114.7 (CH, 4 C, C-10 and C-12 of **12b** and **14b**), 97.0 (CH, 2 C, C-1 of **12b** and **14b**), 76.6 (CH, 2 C, C-5 of **12b** and **14b**), 70.3 (CH_2, 4 C, C-6 of **12b** and **14b**), 70.0 (CH, 2 C, C-2 of **12b** and **14b**), 66.1 (CH_2, C-7 of **12b**), 65.9 (CH_2, C-7 of **14b**), 55.8 (CH_3, C-14 of **12b**), 55.7 (CH_3, C-14 of **2**), 50.6 (CH, 2 C, C-4a of **12b** and **14b**), 49.9 (C, C-3a of **12b**), 49.8 (C, C-3a of **14b**), 49.2 (CH_2, C-8′of **12b**), 47.5 (CH, 2 C, C-4 of **12b** and **14b**), 46.4 (CH_2, C-8′of **14b**), 45.7 (CH, C-7′of **14b**), 44.9 (CH, C-7′of

12b), 43.3 (CH, C-2′of **12b**), 42.4 (CH, C-2′of **14b**), 42.2 (CH, C-4′ of **12b**), 41.3 (CH, C-4′of **14b**), 36.6 (CH, 2 C, C-3 of **12b** and **14b**), 30.9 (CH_2, C-3′of **14b**), 30.2 (CH_2, C-3′of **12b**).

More polar fraction. 12b: Colorless oil; $[\alpha]_D^{32}$ = +16.0 (*c* 1.12, $CHCl_3$); IR (film): 3067, 2953, 1724 (C=O), 1508, 1234, 826, 748 cm^{-1}; ^1H NMR (300 MHz, $CDCl_3$) δ 7.45-7.37 (m, 2 H, aromatics), 7.28-7.16 (m, 4 H, aromatics), 7.14-7.08 (m, 2 H, aromatics), 7.07-7.01 (m, 2 H, H-9 and H-13), 6.95-6.89 (m, 2 H, H-10 and H-12), 6.09 (dd, $J_{5'-6'}$= 5.4 Hz, $J_{5'-4'}$=2.8 Hz, 1 H, H-5′), 5.91 (dd, $J_{5'-6'}$= 5.4 Hz, $J_{6'-7'}$=2.7 Hz, 1 H, H-6′), 4.95-4.93 (m, 2 H, H-7 and H-1), 4.67-4.64 (m, 2 H, H-7 and H-5), 4.26 (dd, J_{2-3}= 6.2 Hz, J_{1-2}= 3.4 Hz, 1 H, H-2), 4.21 (s, 1 H, H-4a), 3.79 (s, 3 H, H-8), 3.74-3.69 (m, 2 H, H-6), 2.81-2.76 (m, 3 H, H-3, H-4′ and H-7′), 2.33 (bs, 1 H, H-2′), 2.23 (d, J_{3-4}= 10.6 Hz, 1 H, H-4), 1.78-1.70 (m, 1 H, H-3′), 1.29-1.27 (m, 1 H, H-8′), 1.17 (d, J_{gem}= 11.3 Hz, 1 H, H-3′), 1.00 (d, J_{gem}=7.9 Hz, 1 H, H-8′); ^{13}C NMR (75.5 MHz, $CDCl_3$) δ 174.5 (C, C-1′), 154.3 (C, C-11), 152.9 (C, C-8), 146.0 (C, aromatic), 141.2 (C, aromatic), 140.7 (C, aromatic), 140.7 (C, aromatic), 137.3 (CH, C-5′), 133.1 (CH, C-6′), 126.6 (CH, aromatic), 126.1 (CH, aromatic), 126.0 (CH, aromatic), 126.0 (CH, aromatic), 125.2 (CH, aromatic), 124.2 (CH, aromatic), 122.9 (CH, aromatic), 122.1 (CH, aromatic), 115.3 (CH, 2 C, C-9 and C-13), 114.9 (CH, 2 C, C-10 and C-12), 97.1 (CH, C-1), 76.7 (CH, C-5), 70.4 (CH_2, C-6), 70.2 (CH, C-2), 66.3 (CH_2, C-7), 55.9 (CH_3, C-14), 50.8 (CH, C-4a), 50.0 (C, C-3a), 49.3 (CH_2, C-8′), 47.6 (CH, C-4), 45.0 (CH, C-7′), 43.4 (CH, C-2′), 42.4 (CH, C-4′), 36.8 (CH, C-3), 30.4 (CH, C-3′); HRMS calc. for $C_{36}H_{34}O_6Na$ $[M+Na]^+$ 585.2248. Found 585.2232.

Hydrolysis and esterification of adducts 11a–14a and 11b–14b.

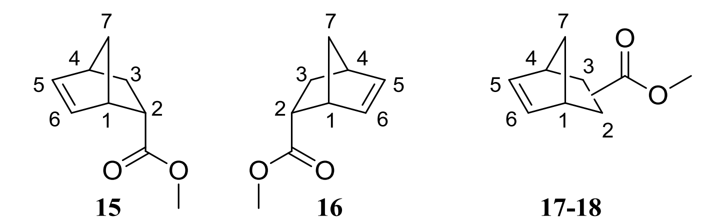

15 **16** **17-18**

A mixture of adducts of known composition (0.06 mmol) was dissolved in THF/H_2O 2:1 (5.6 mL) and LiOH·H_2O (27.6 mg, 0.66 mmol) was added. The reaction was stirred at room temperature for 20 d and then a NaOH solution (1 M, 6 mL) was added. The aqueous phase was extracted with Et_2O (3 × 12 mL). The combined organic extracts were dried (Na_2SO_4) and the solvent was evaporated to recover the chiral auxiliary quantitatively. The aqueous phase was neutralized with 1 N HCl to reach pH 4 and extracted with ether (5 × 12 mL). The combined organic extracts were dried (Na_2SO_4) and the solvent was evaporated to obtain a mixture of isomers of 5-norbornene-2-carboxylic acid (8 mg, 0.06 mmol, 100%). The residue was dissolved in Et_2O, cooled to 0 °C and then an excess of an ethereal solution of diazomethane was added. After stirring the mixture for 15 min a few drops of glacial acetic acid were added to destroy any excess of diazomethane. The solution was dried (Na_2SO_4) and the solvent was evaporated to obtain a mixture of isomers of methyl 5-norbornene-2-carboxylate **15–18**. This procedure was performed for adducts **11a–14a** and

11b–14b, and for adducts derived from analogous quiral auxiliaries that were used as standars of know configuration [3].

The analysis of these samples by GC–MS allowed us to determine the absolute configuration at C-2 in the adducts **11a–14a** and **11b–14b** by comparison of the retention times of isomers **15–18** obtained by hydrolysis and esterification of different adducts. Retention times were 6.17 and 6.30 min for **17**,**18**, 7.07 min for **16** and 7.20 min for **15** (Figure S3).

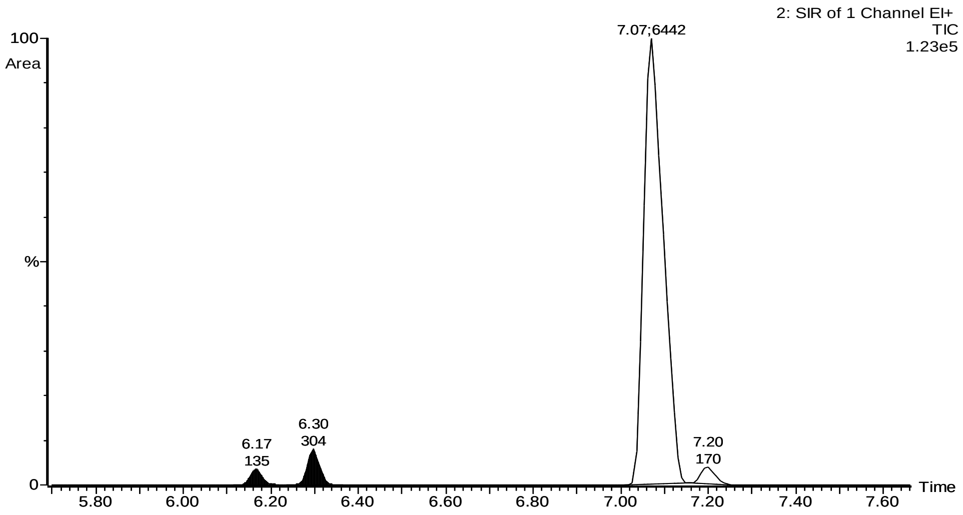

Figure S3

NMR spectra of acrylates with Et_2AlCl

Complexation of the acrylates with Lewis acids: for the ^{1}H NMR experiments the corresponding acrylic ester (0.02 mmol) was dissolved in dry $CDCl_3$ (0.3 mL) in an NMR tube under nitrogen and Et_2AlCl (22 μL, 1.8 M in toluene) was added to the solution. The acrylates showed to be stable during the time required for the acquisition of the spectra.

[3] a) Sarotti, A. M.; Spanevello, R. A.; Duhayon, C.; Tuchagues, J. P.; Suárez A. G. *Tetrahedron* **2007**, *63*, 241-251. b) Sarotti, A. M.; Spanevello, R. A.; Suárez, A. G.*Org. Lett.* **2006**, *8*, 1487-1490.

^1H NMR (300 MHz, $CDCl_3$) Spectra of compound **1**

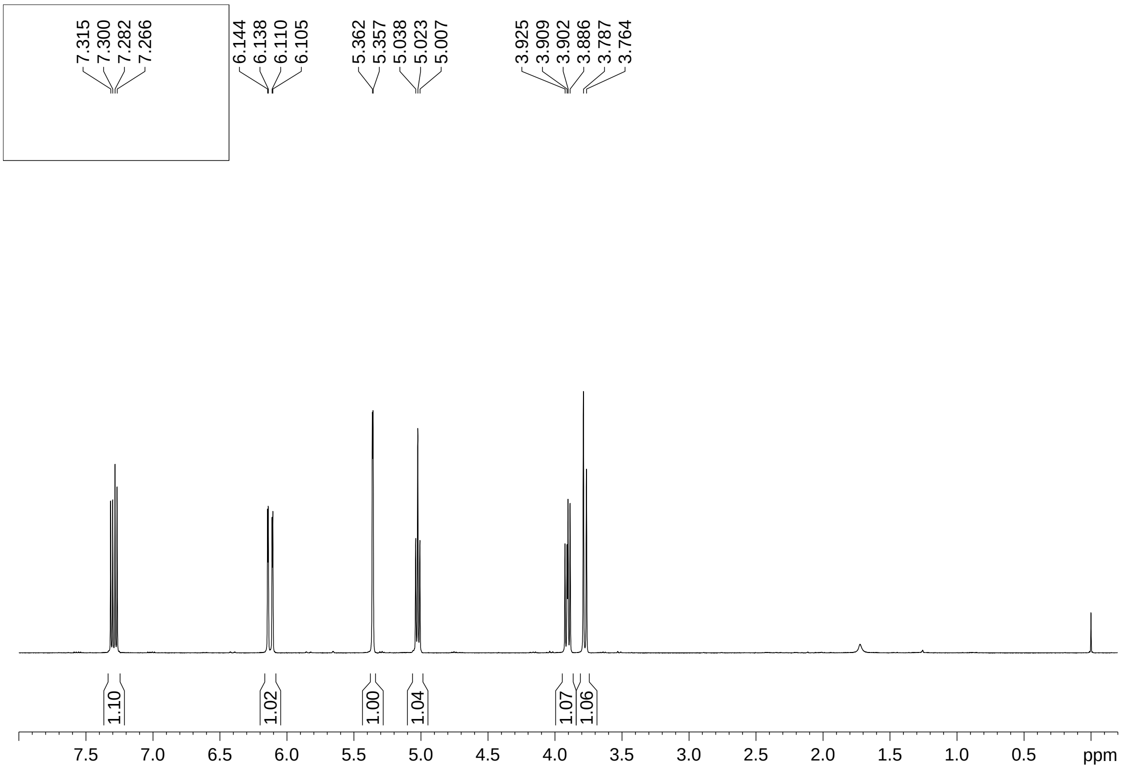

^{13}C NMR (75.5 MHz, $CDCl_3$) Spectra of compound **1**

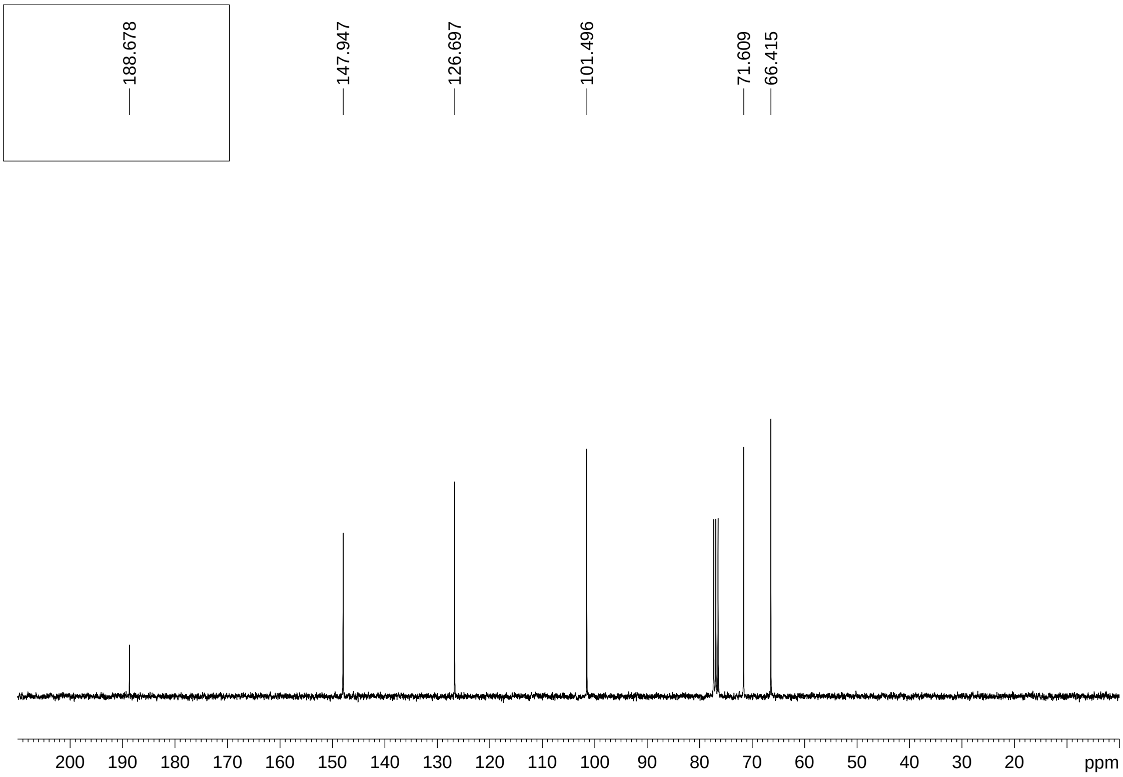

^{1}H NMR (300 MHz, $CDCl_3$) Spectra of compound **2a**

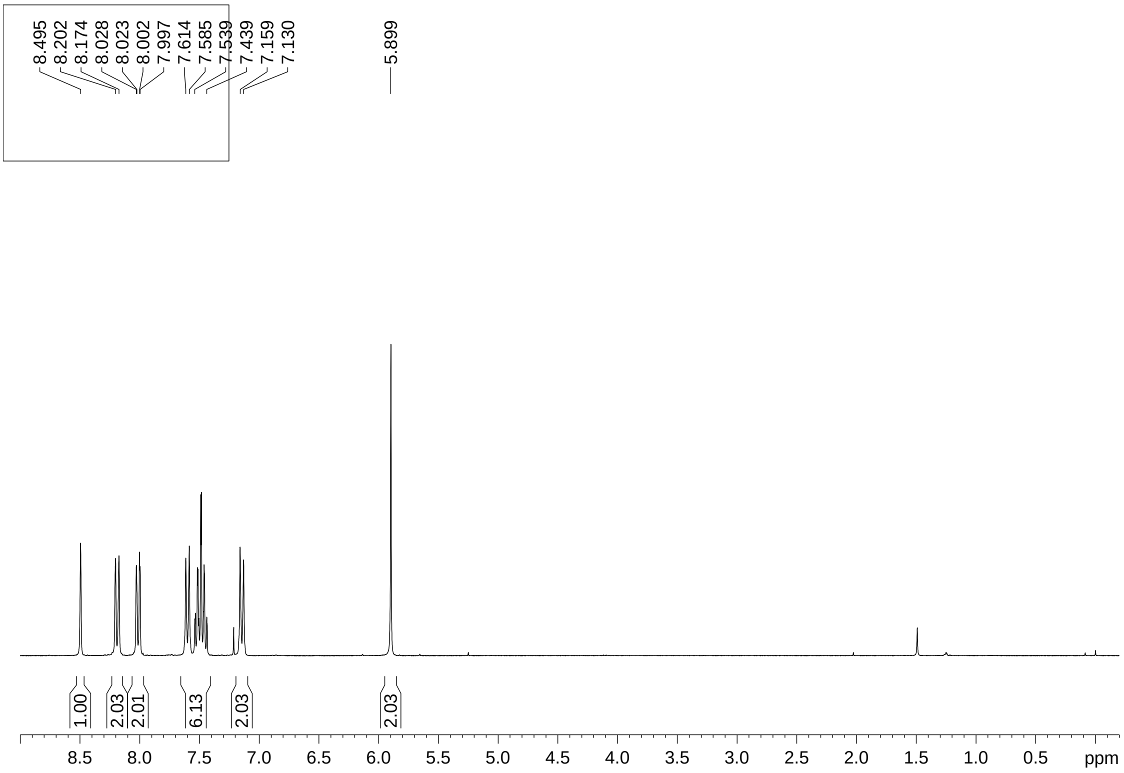

^{13}C NMR (75,5 MHz, $CDCl_3$) Spectra of compound **2a**

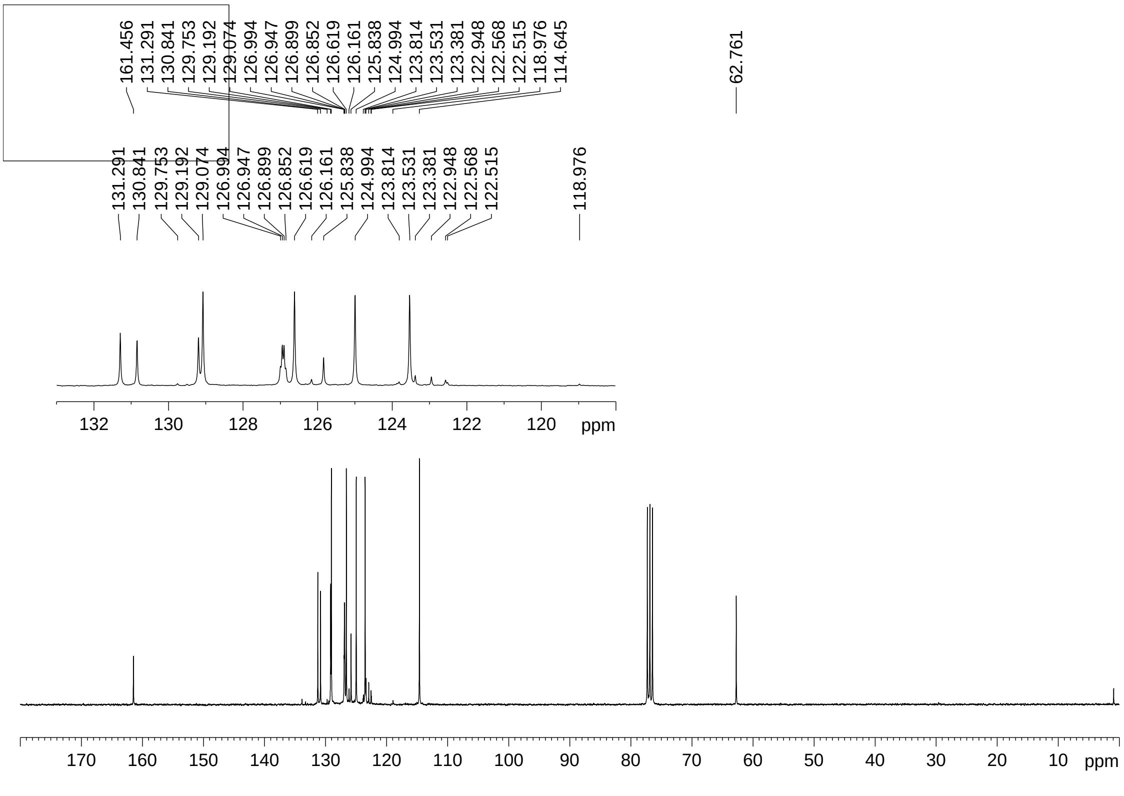

^{1}H NMR (300 MHz, $CDCl_3$) Spectra of compound **3a**

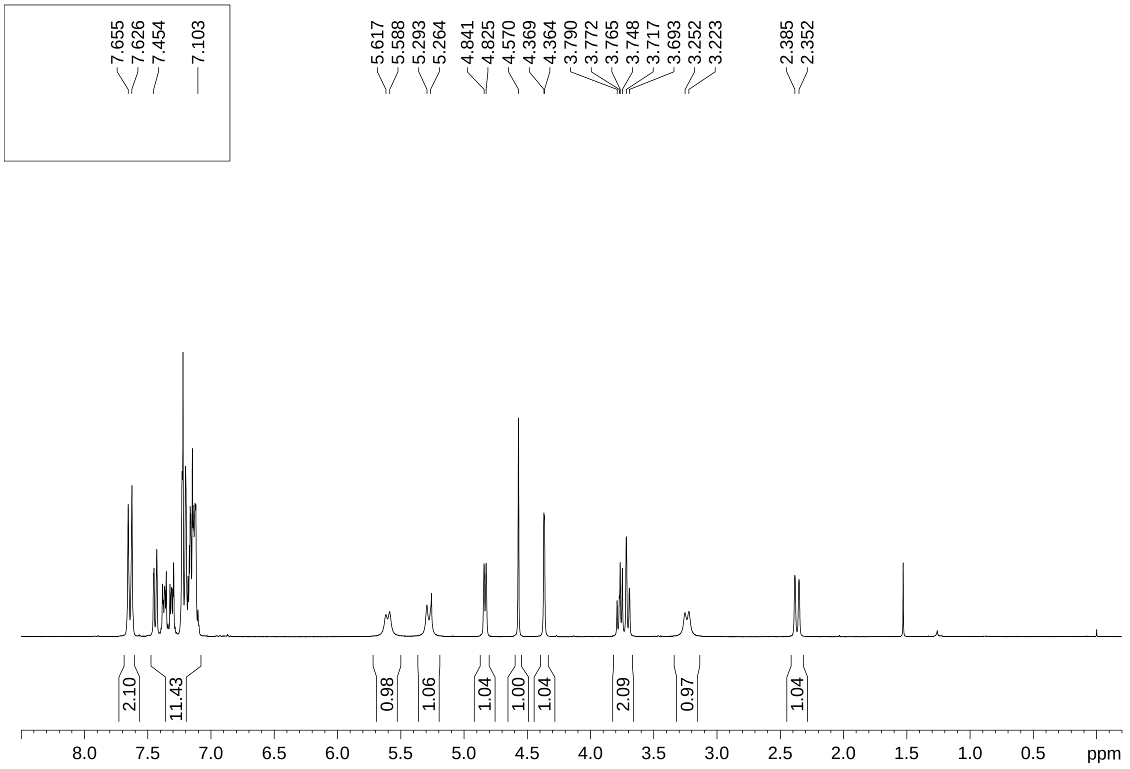

^{13}C NMR (75,5 MHz, $CDCl_3$) Spectra of compound **3a**

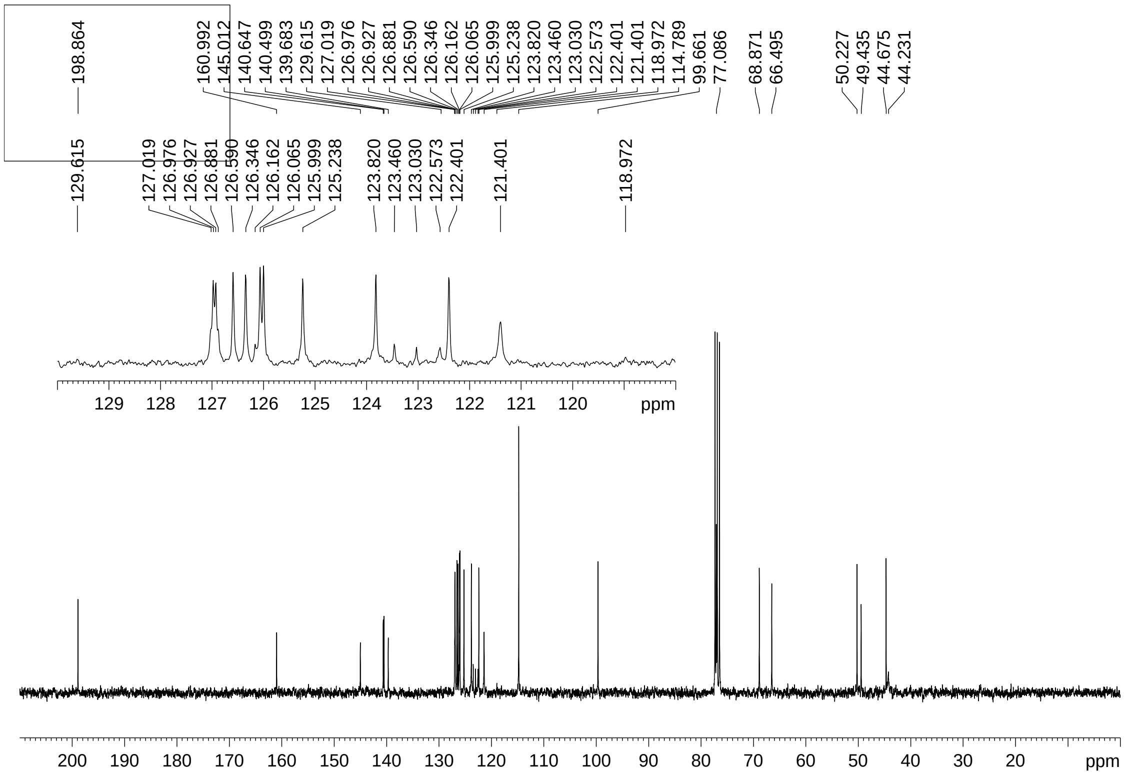

^{1}H NMR (300 MHz, $CDCl_3$) Spectra of compound **4a**

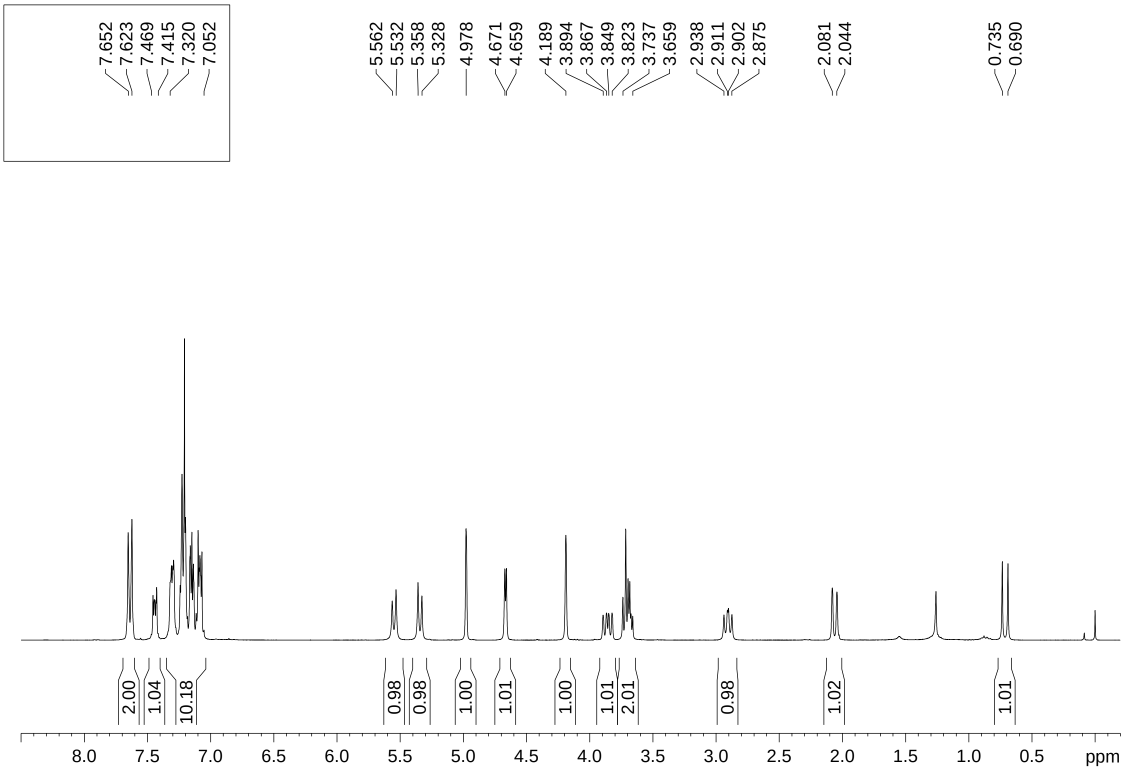

^{13}C NMR (75,5 MHz, $CDCl_3$) Spectra of compound **4a**

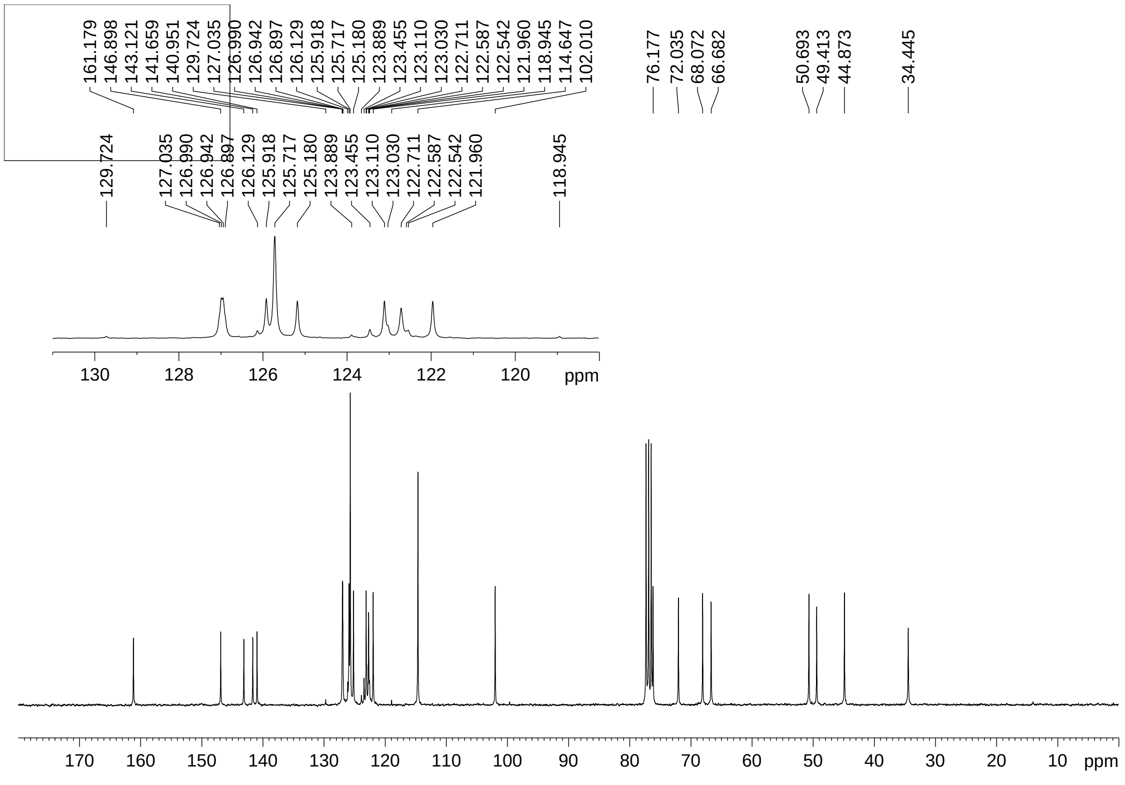

^{1}H NMR (300 MHz, $CDCl_3$) Spectra of compound **5a**

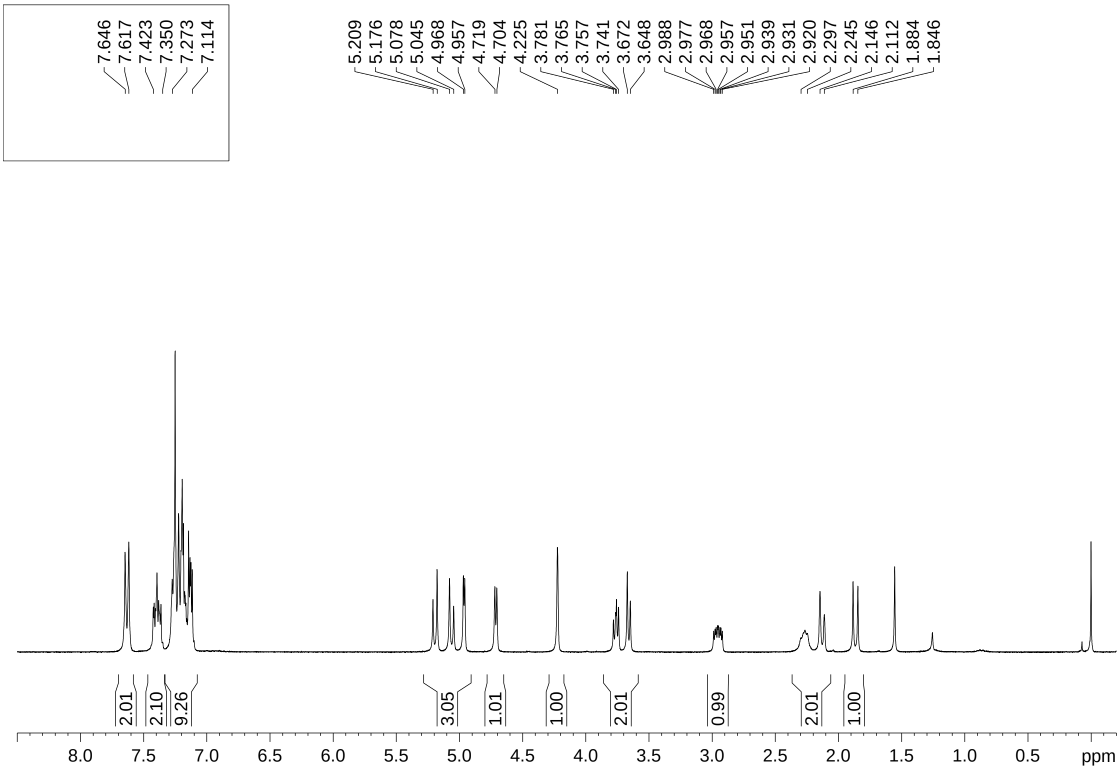

^{13}C NMR (75,5 MHz, $CDCl_3$) Spectra of compound **5a**

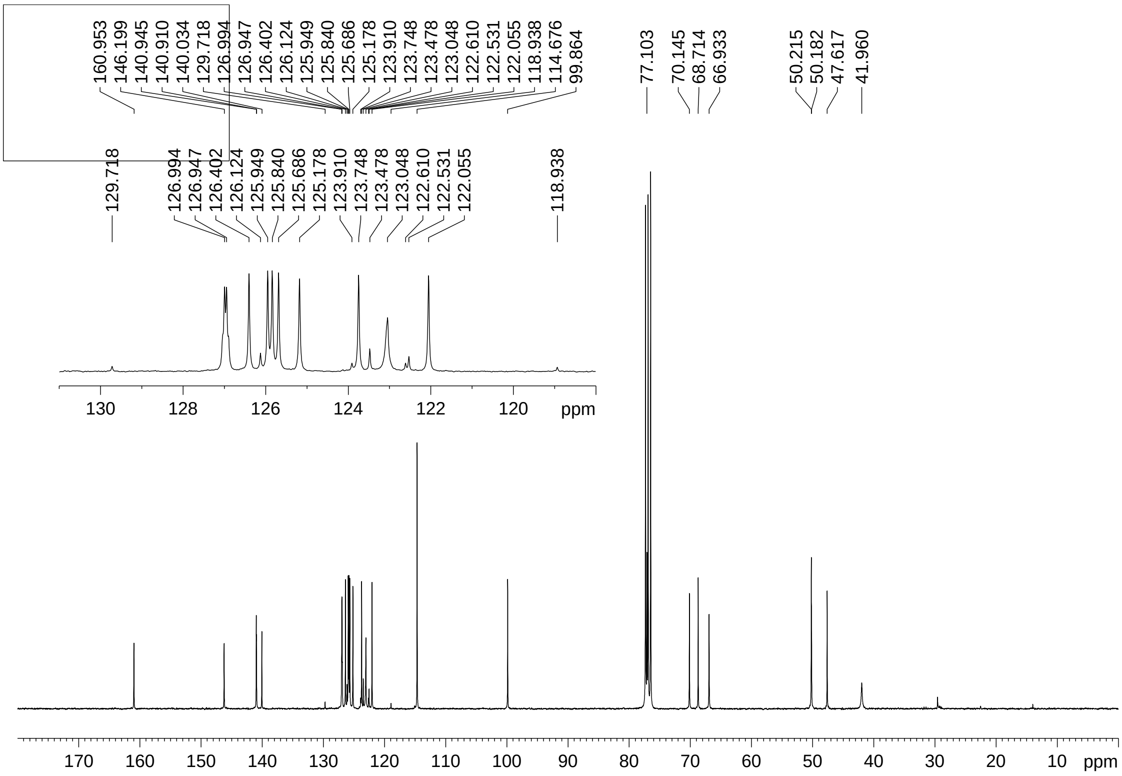

^{1}H NMR (300 MHz, $CDCl_3$) Spectra of compound **6a**

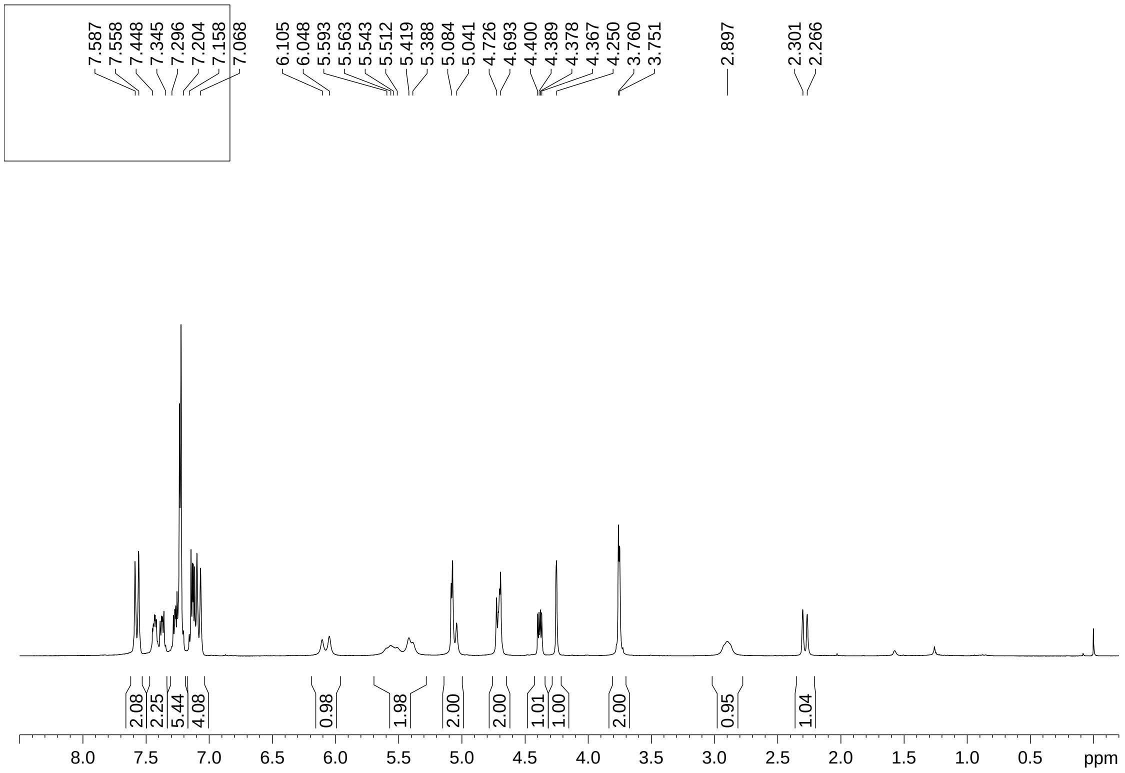

^{13}C NMR (75,5 MHz, $CDCl_3$) Spectra of compound **6a**

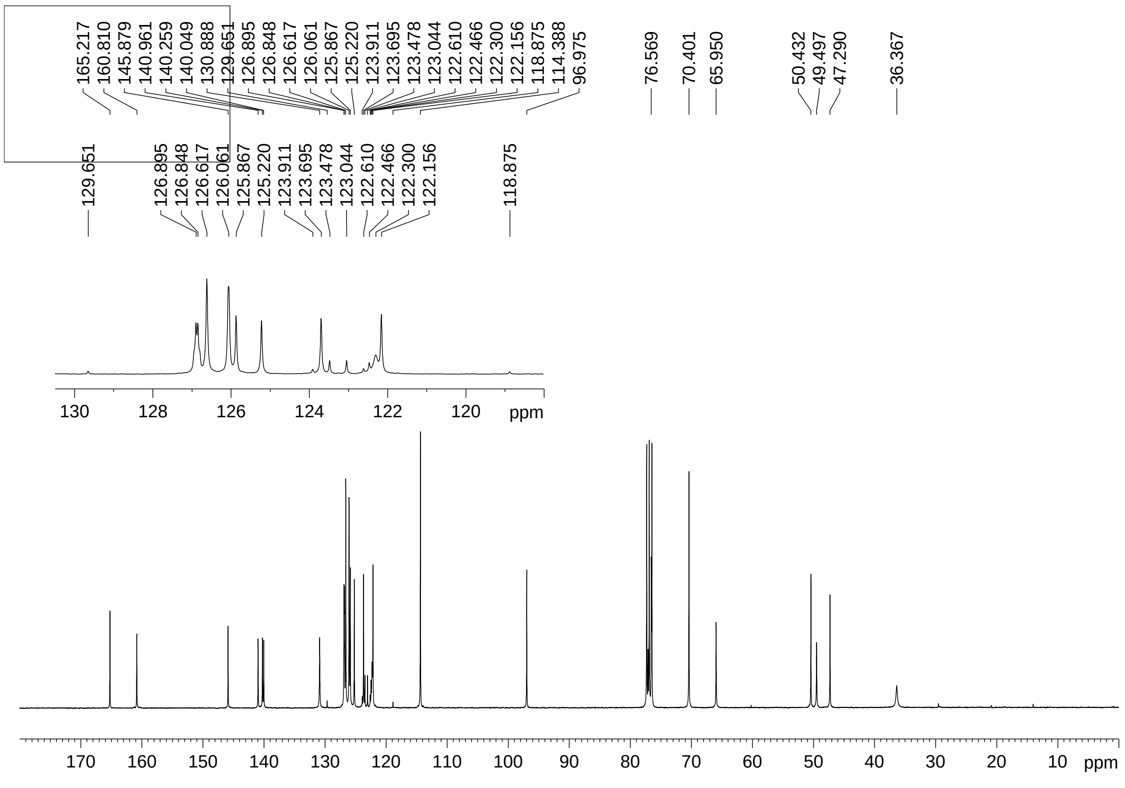

^{1}H NMR (300 MHz, $CDCl_3$) Spectra of compound **11a** and **13a**

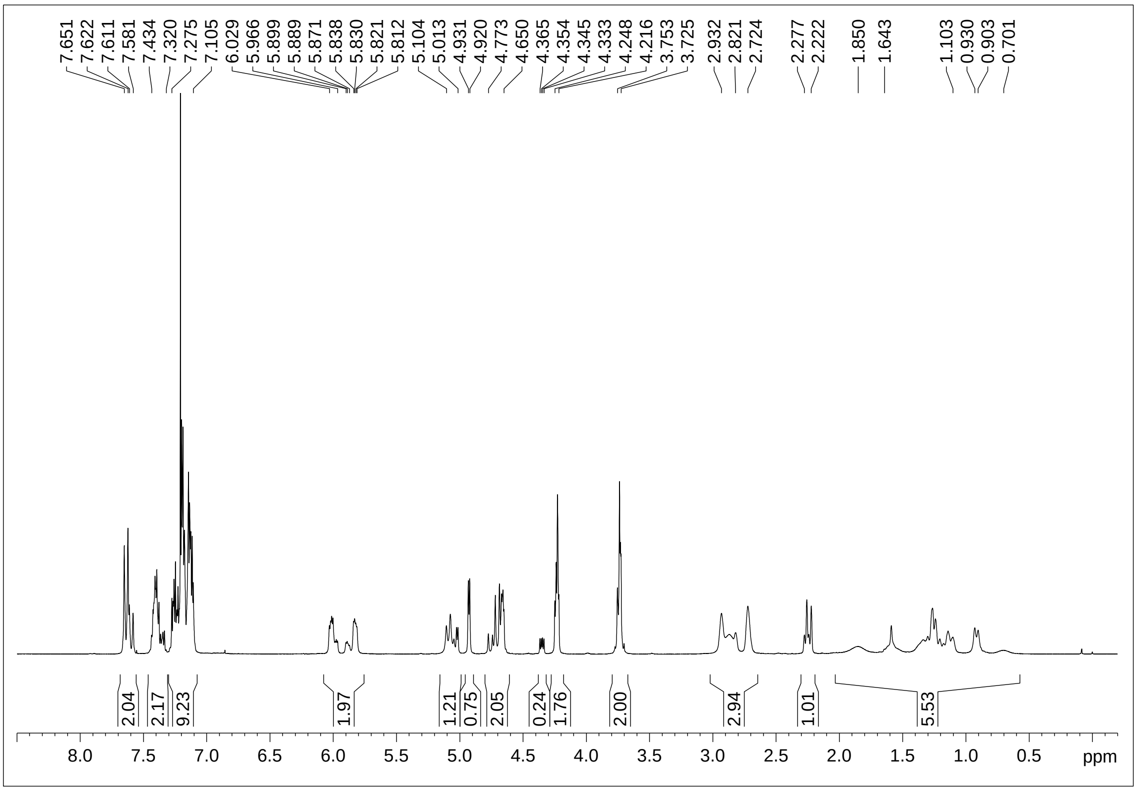

^{13}C NMR (75.5 MHz, $CDCl_3$) Spectra of compound **11a** and **13a**

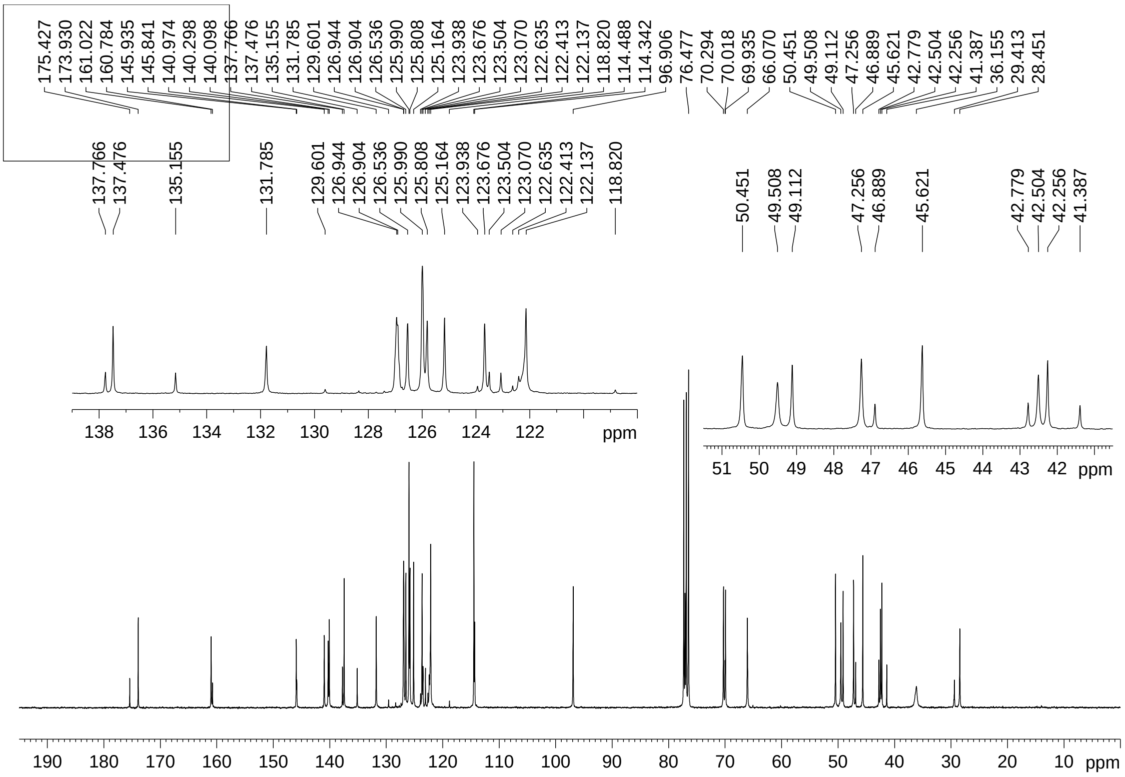

^{1}H NMR (300 MHz, $CDCl_3$) Spectra of compound **12a** and **14a**

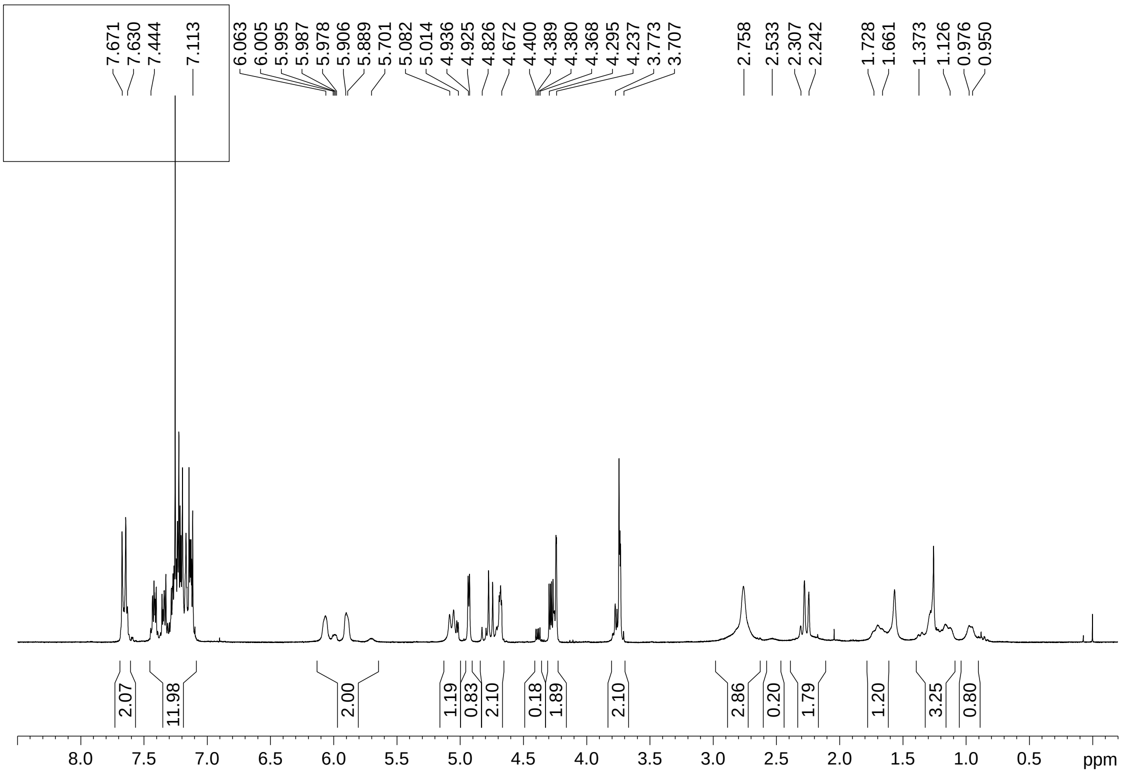

^{13}C NMR (75,5 MHz, $CDCl_3$) Spectra of compound **12a** and **14a**

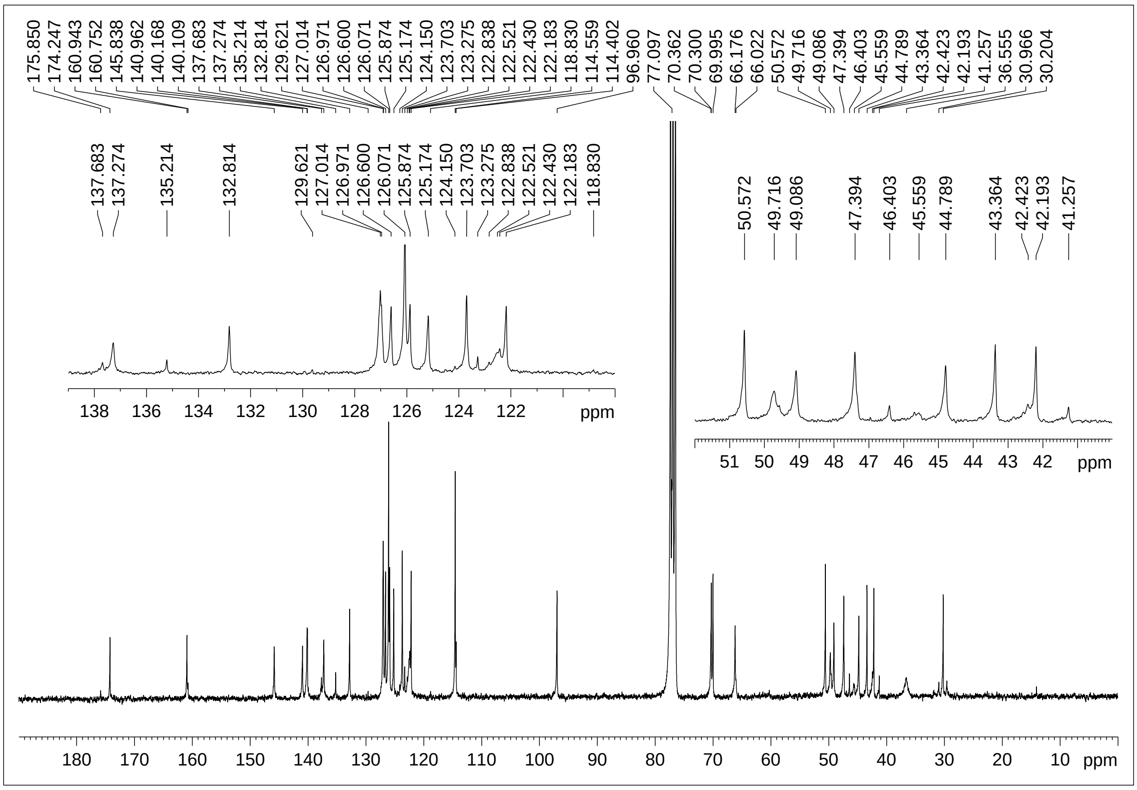

^{1}H NMR (300 MHz, $CDCl_3$) Spectra of compound **2b**

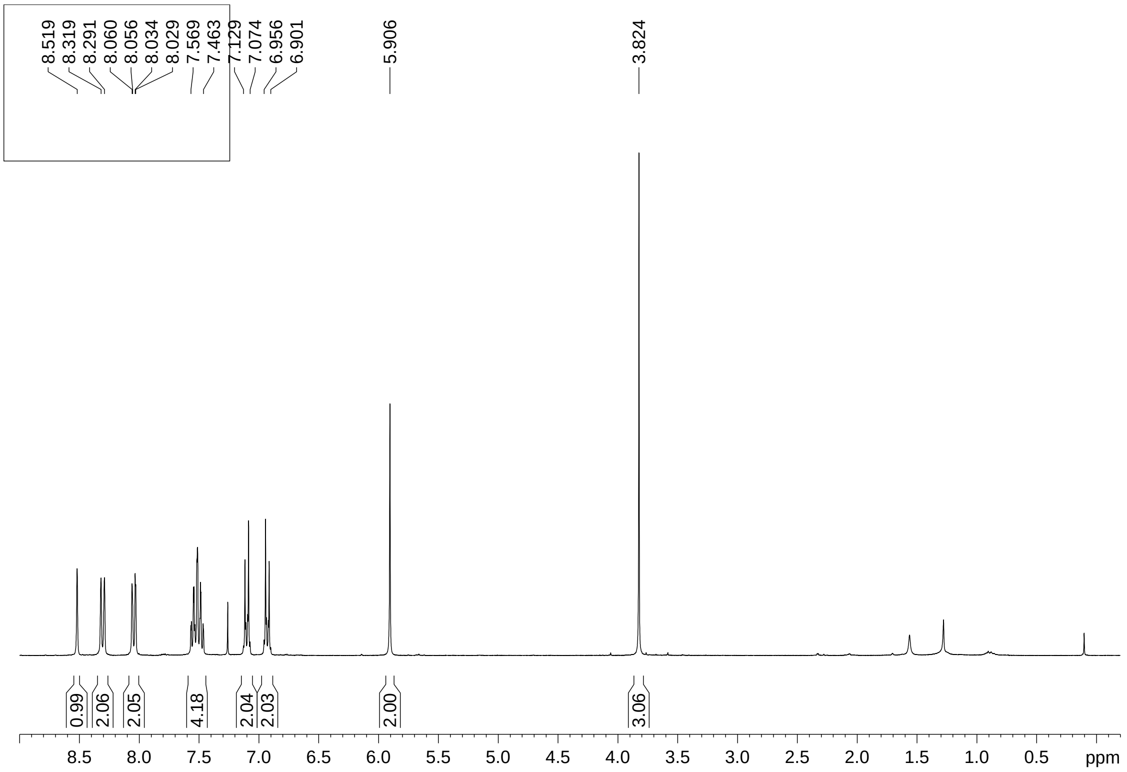

^{13}C NMR (75,5 MHz, $CDCl_3$) Spectra of compound **2b**

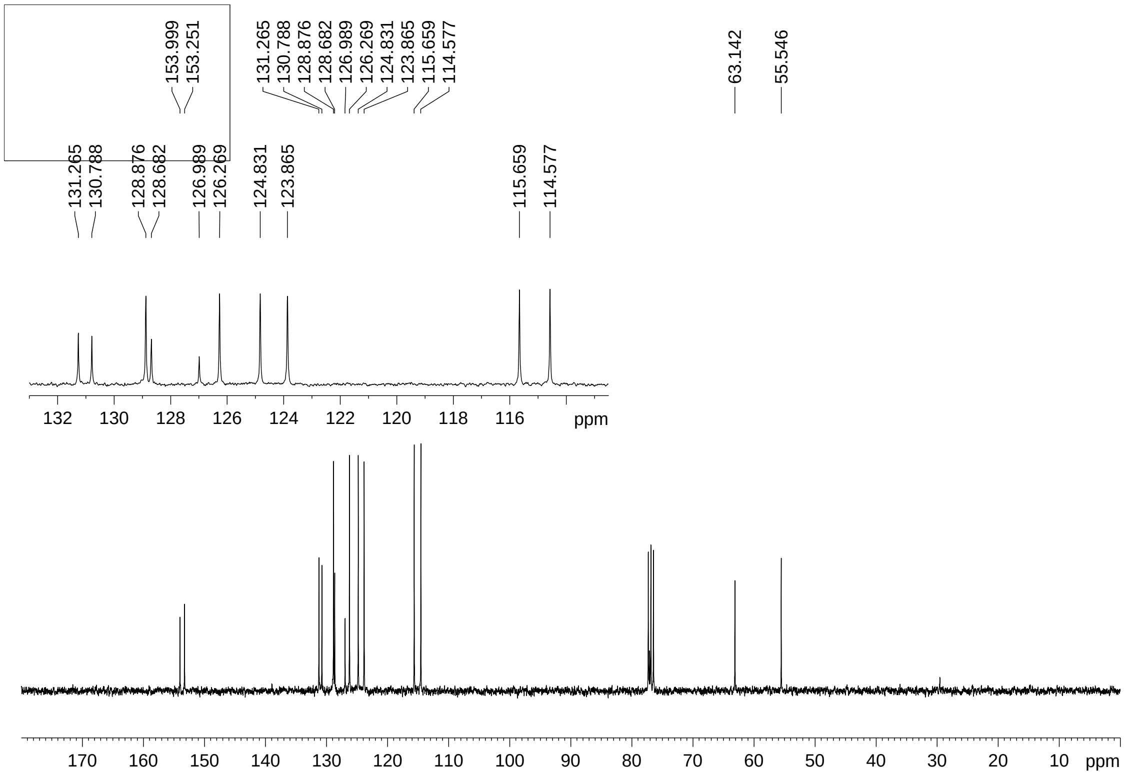

^{1}H NMR (300Hz, $CDCl_3$) Spectra of compound **3b**

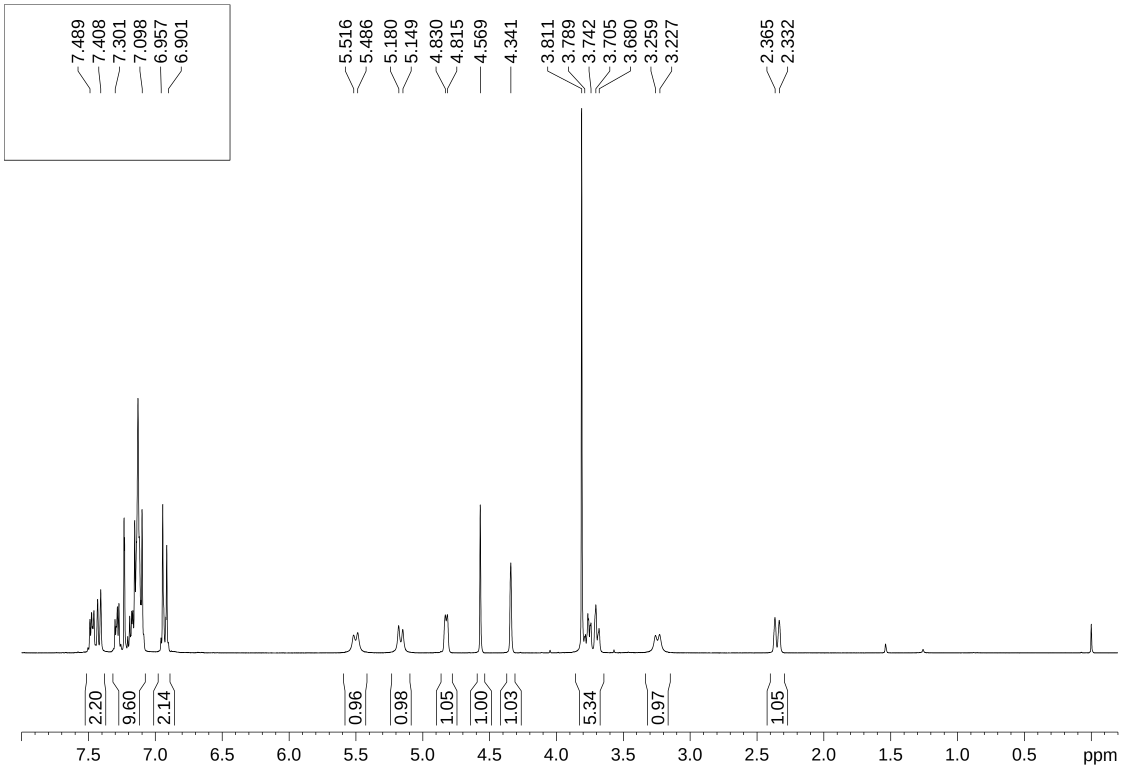

^{13}C NMR (75.5 MHz, $CDCl_3$) Spectra of compound **3b**

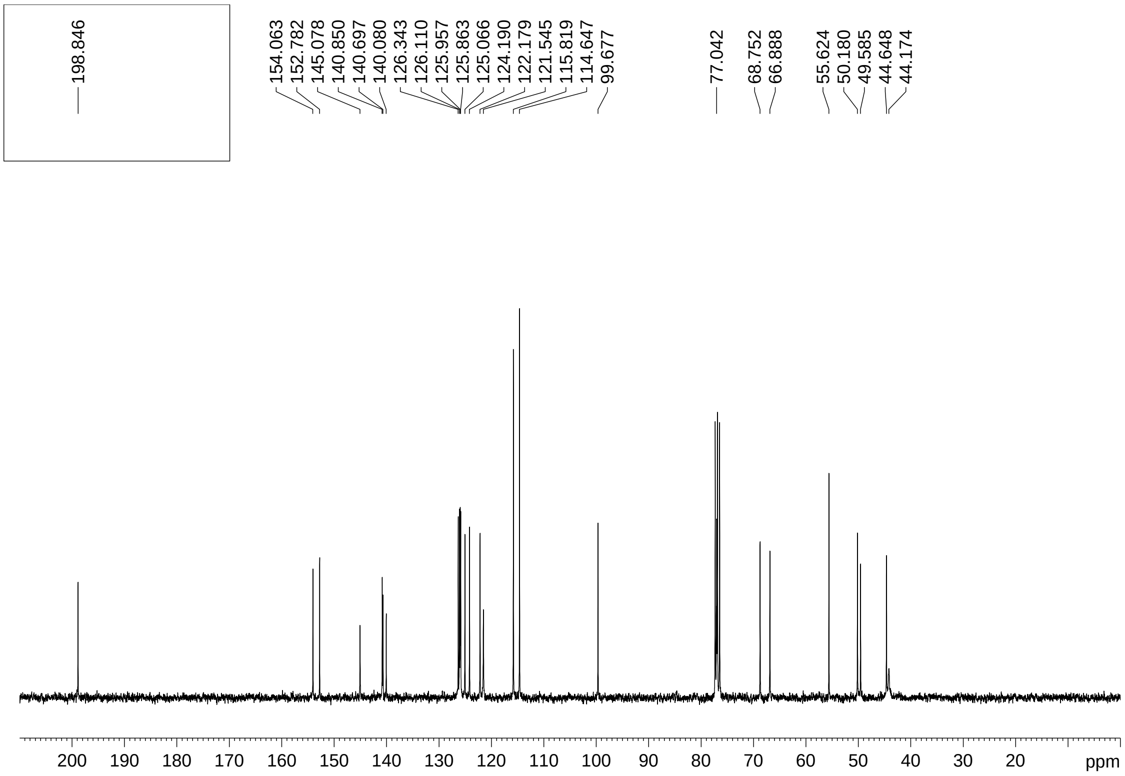

^{1}H NMR (300 MHz, $CDCl_3$) Spectra of compound **4b**

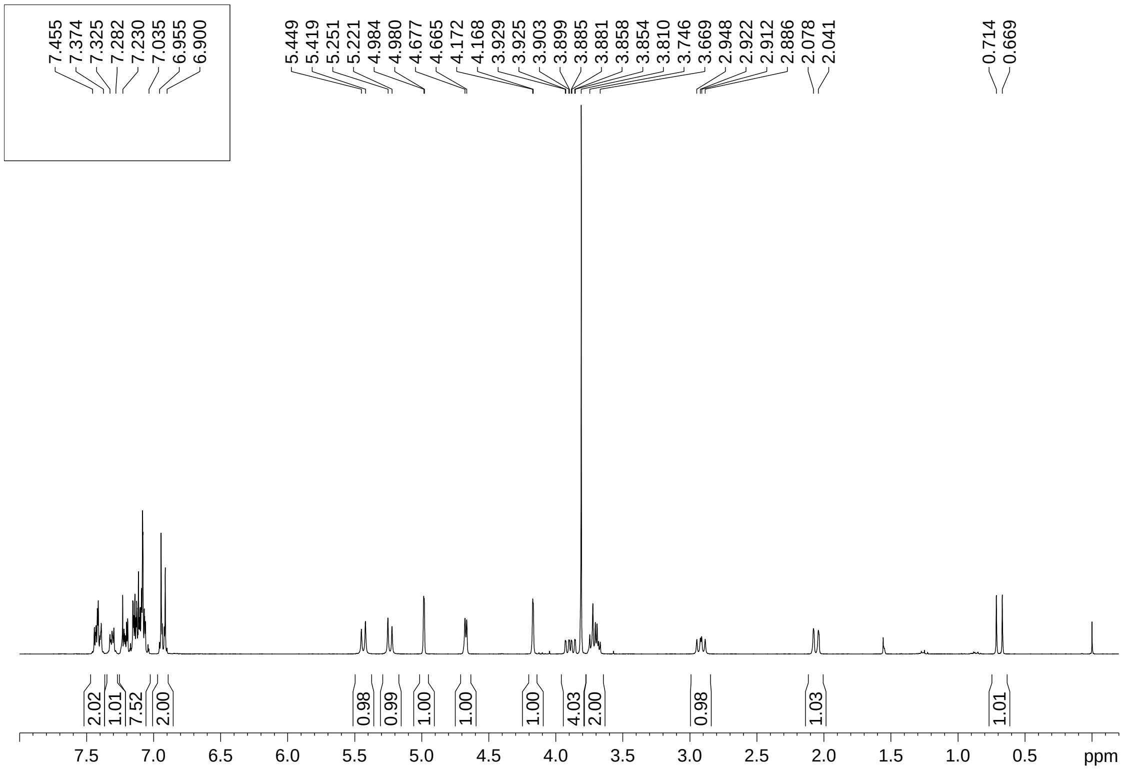

^{13}C NMR (75.5 MHz, $CDCl_3$) Spectra of compound **4b**

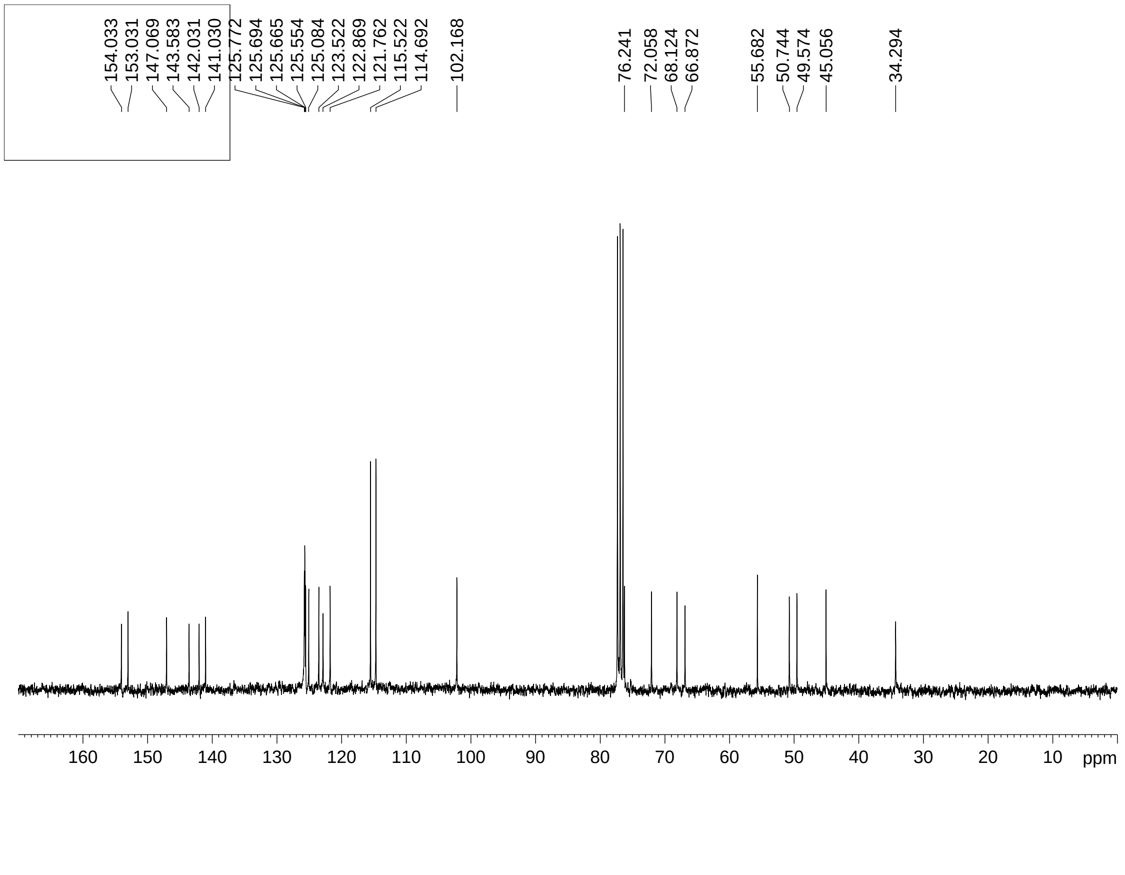

^{1}H NMR (300MHz, $CDCl_3$) Spectra of compound **5b**

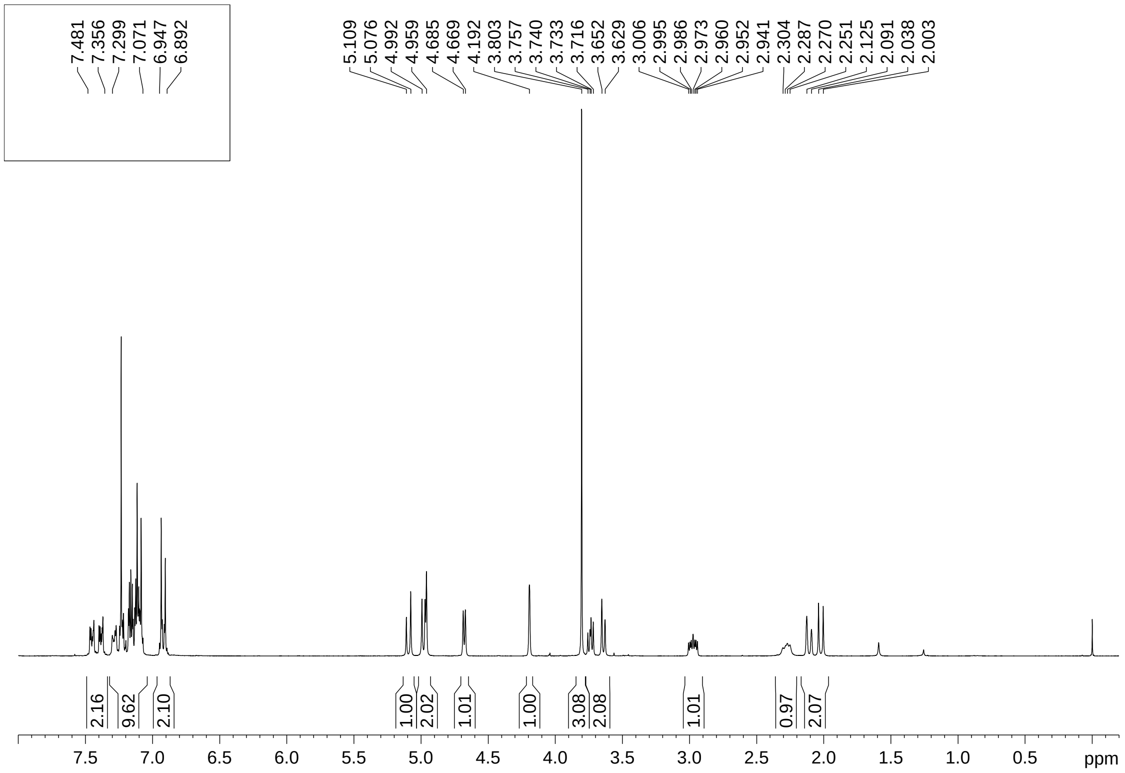

^{13}C NMR (75.5 MHz, $CDCl_3$) Spectra of compound **5b**

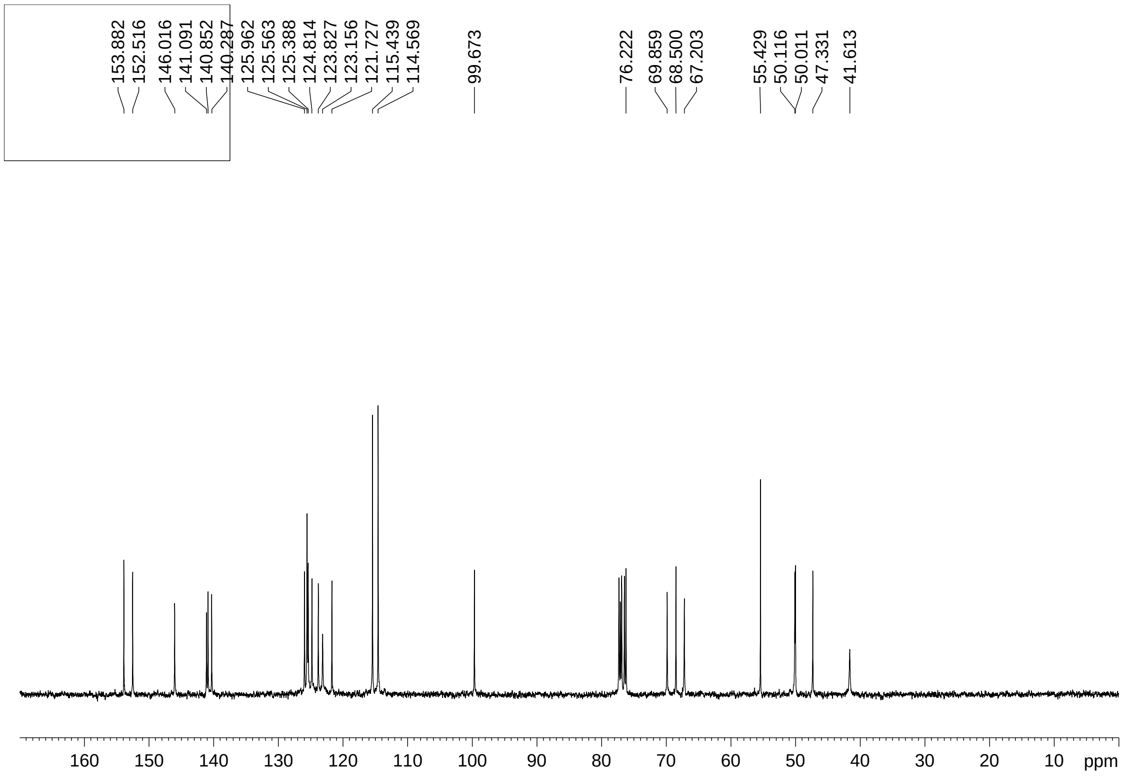

^{1}H NMR (300 MHz, $CDCl_3$) Spectra of compound **6b**

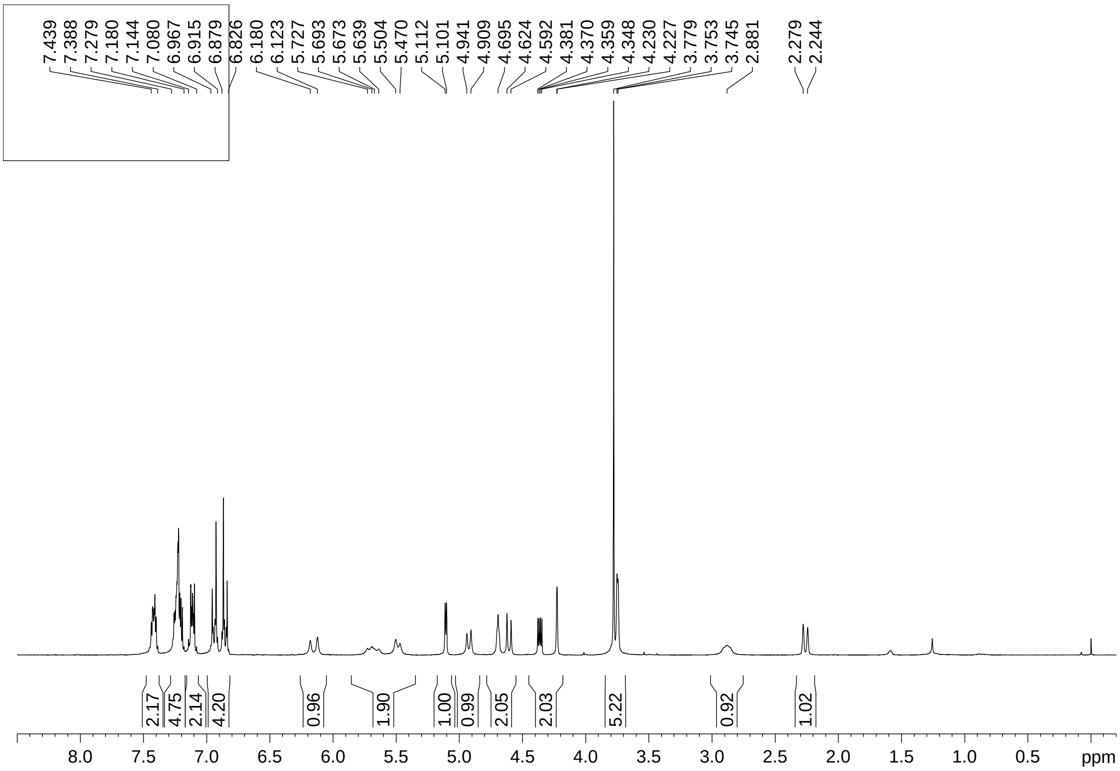

^{13}C NMR (75.5 MHz, $CDCl_3$) Spectra of compound **6b**

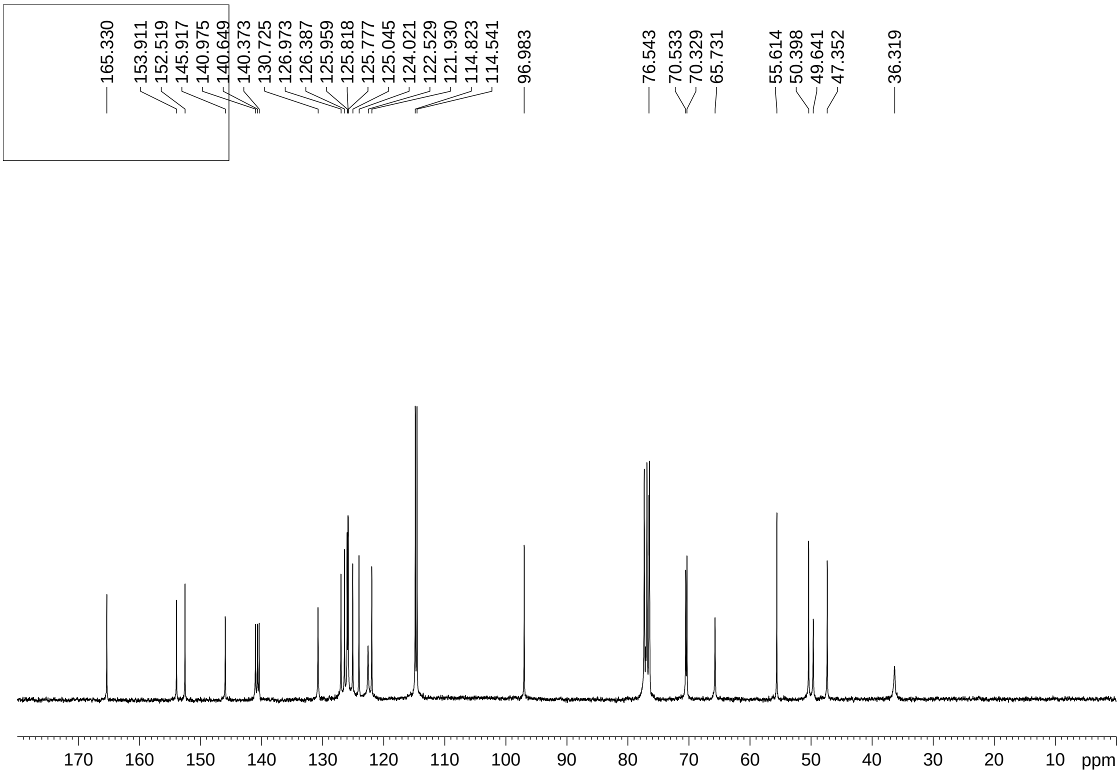

^{1}H NMR (300 MHz, $CDCl_3$) Spectra of compound **11b** and **13b**

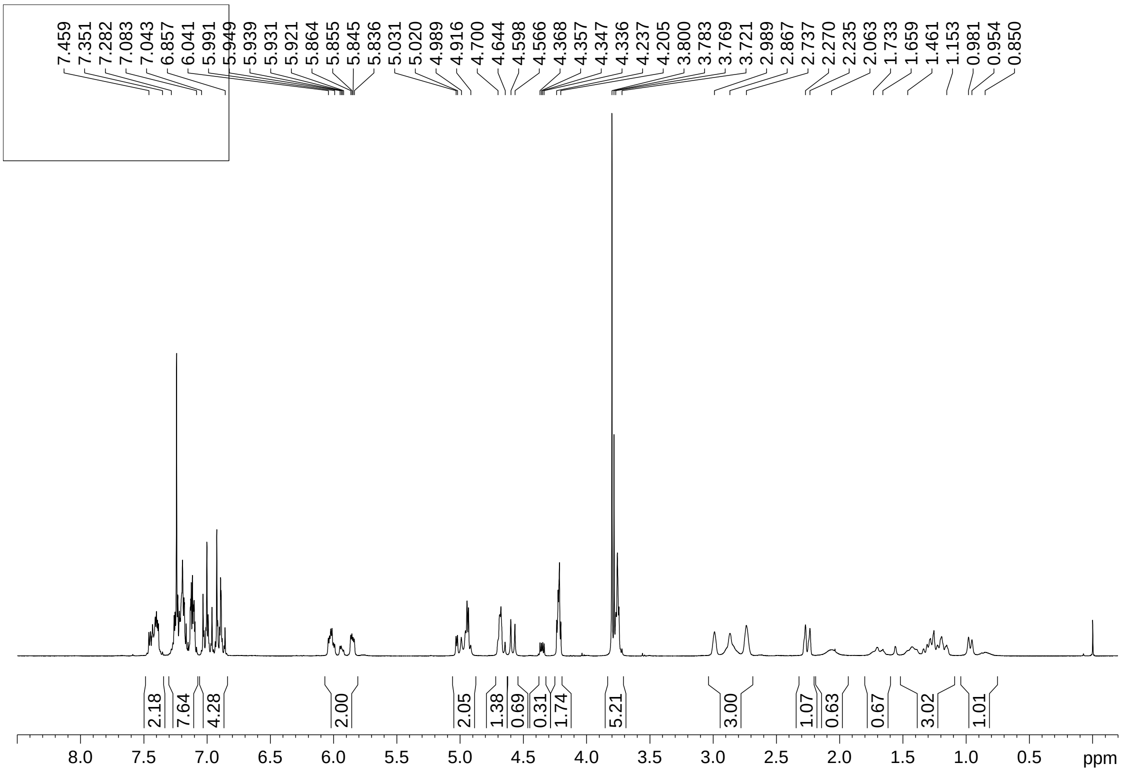

^{13}C NMR (75.5 MHz, $CDCl_3$) Spectra of compound **11b** and **13b**

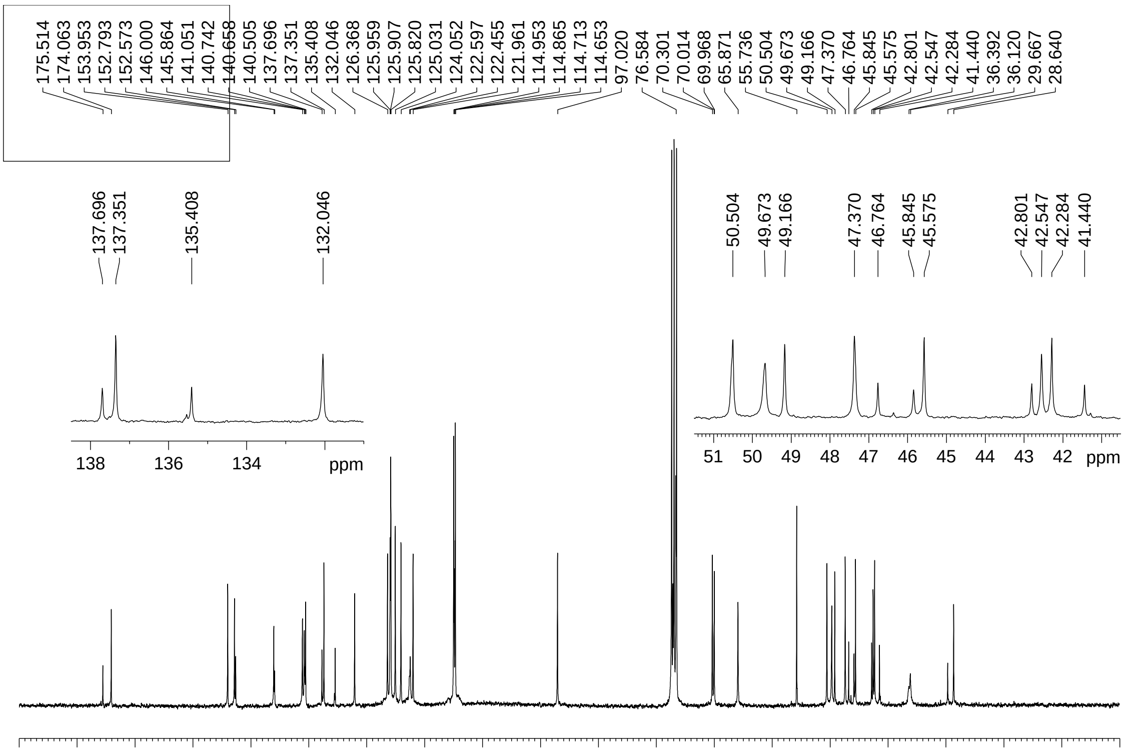

^{1}H NMR (300 MHz, $CDCl_3$) Spectra of compound **12b** and **14b**

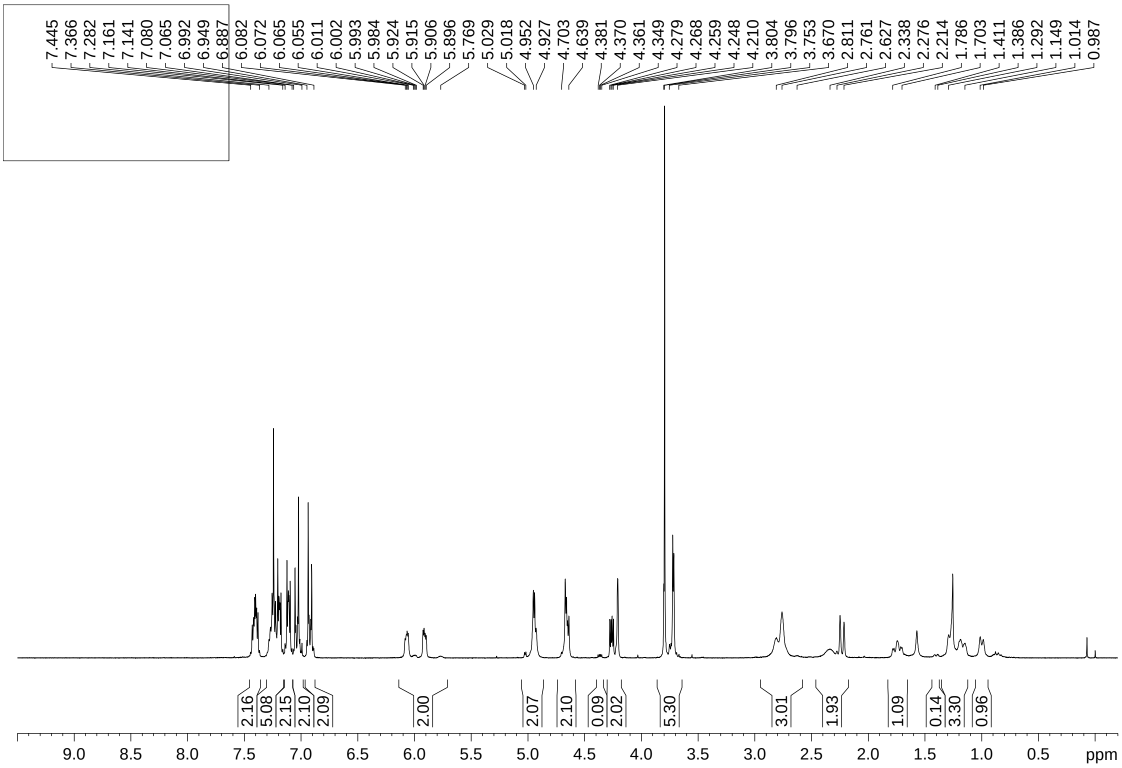

^{13}C NMR (75.5 MHz, $CDCl_3$) Spectra of compound **12b** and **14b**

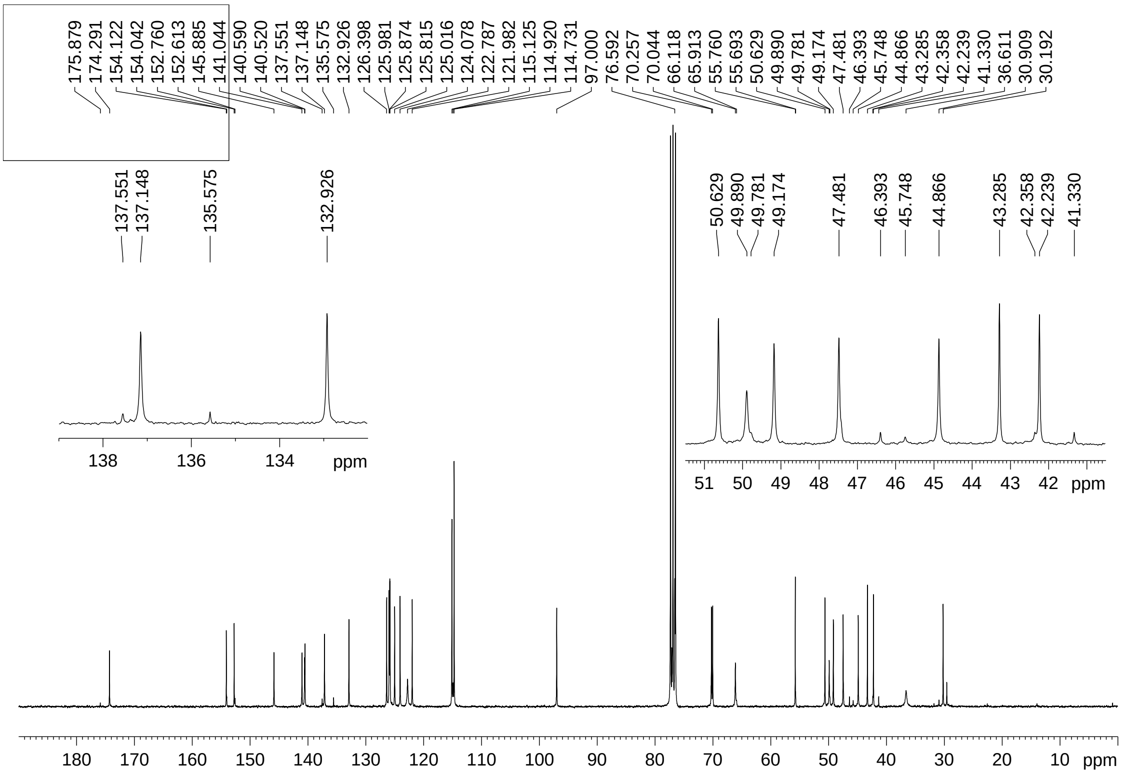

^{1}H NMR (300 MHz, $CDCl_3$) Spectra of compound **12b**

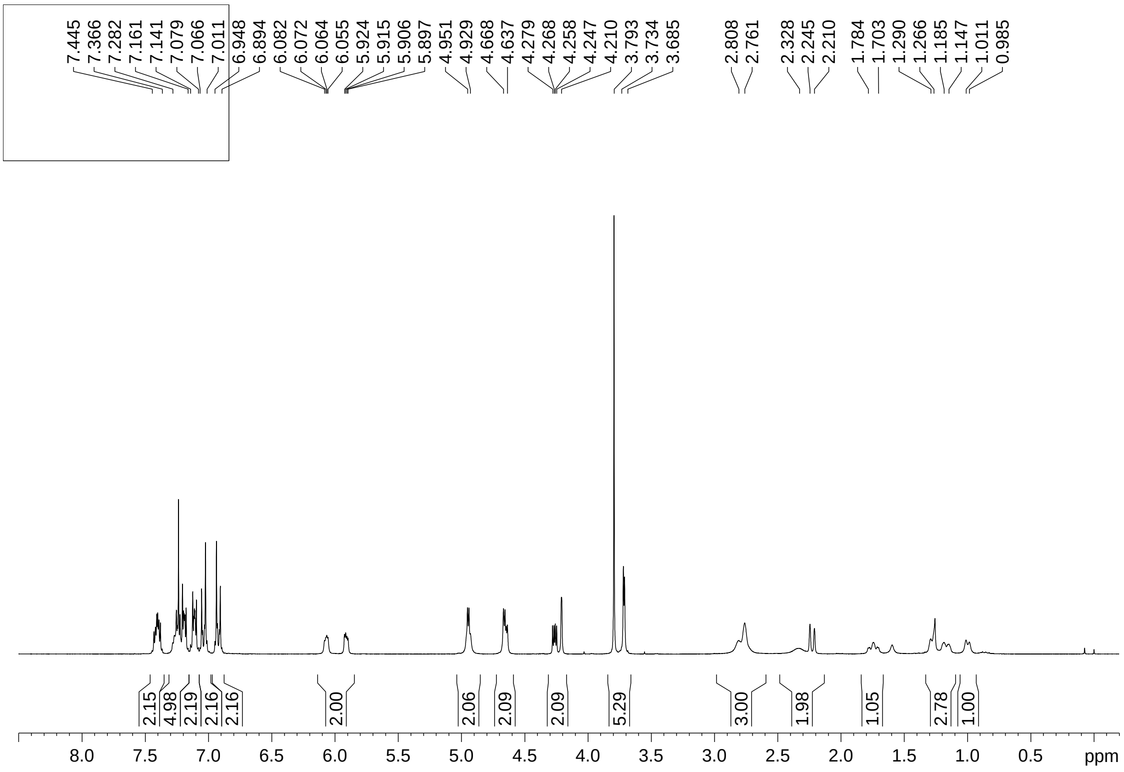

^{13}C NMR (75.5 MHz, $CDCl_3$) Spectra of compound **12b**

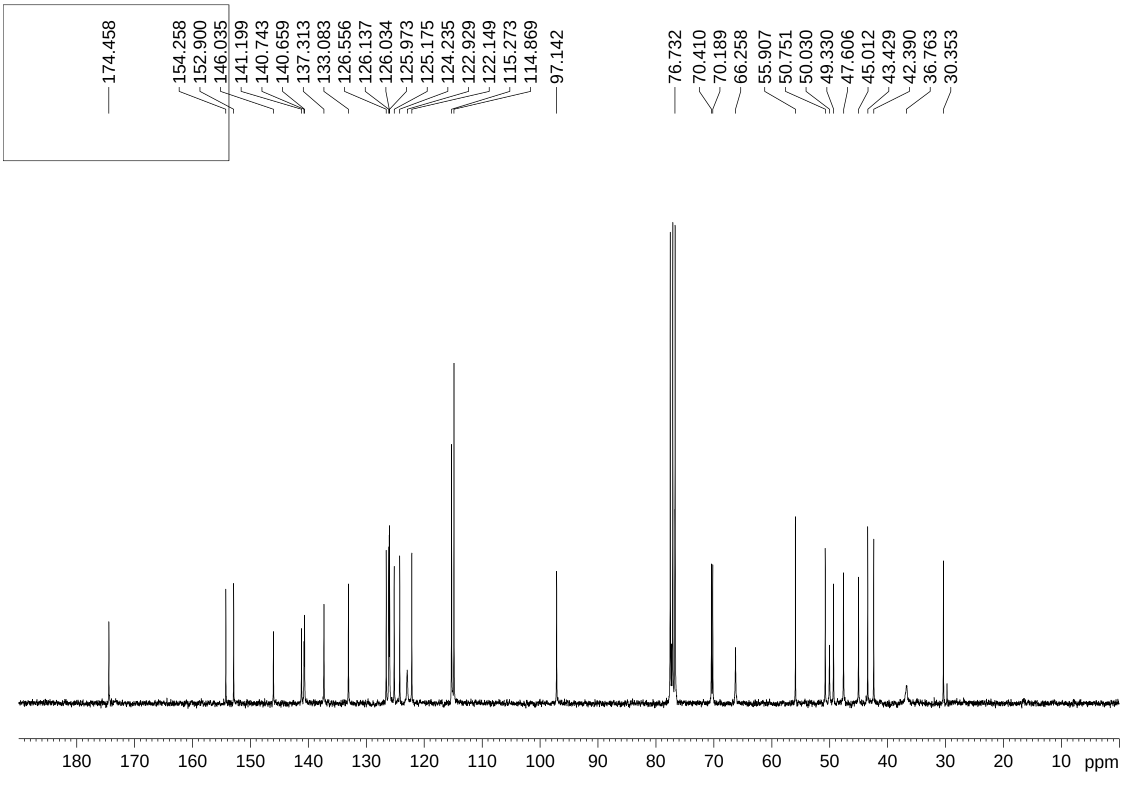

^{1}H NMR (200 MHz and 300 MHz, $CDCl_3$) Spectra of related acrylates[2a,b]

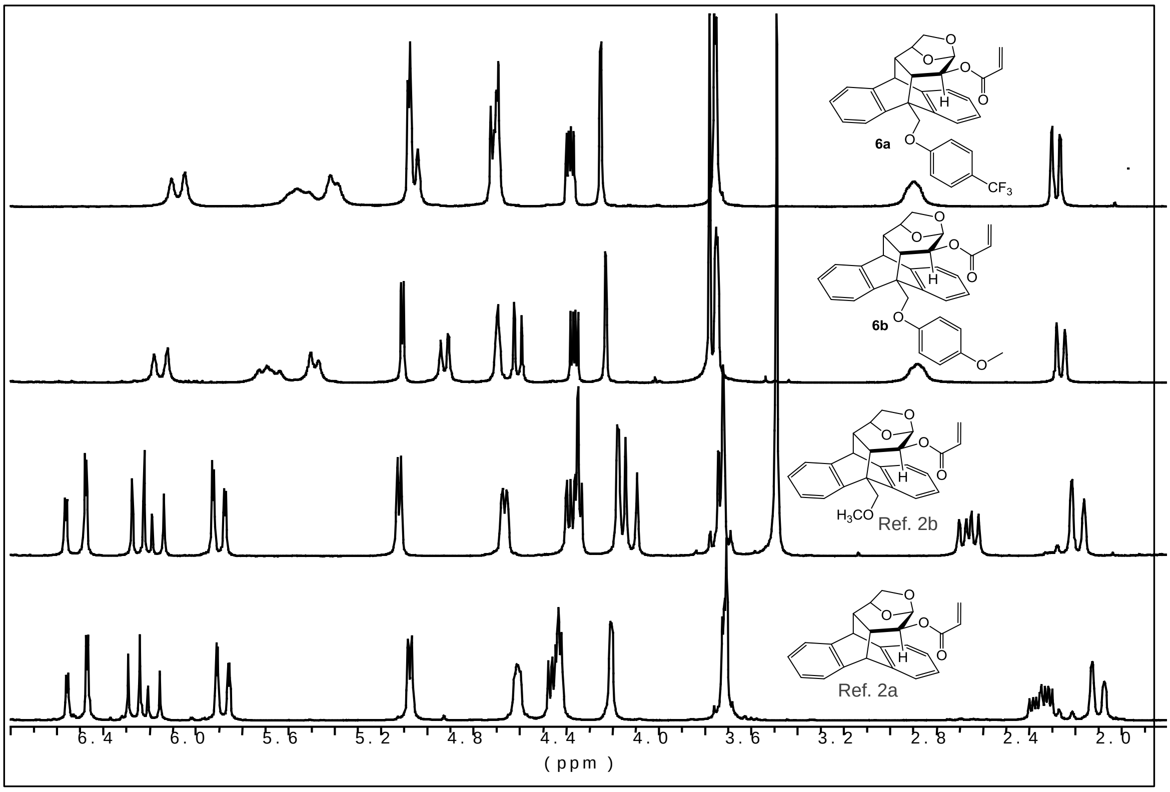

Variable temperature ^{1}H NMR (300 MHz, $CDCl_3$) Spectra of **6a**

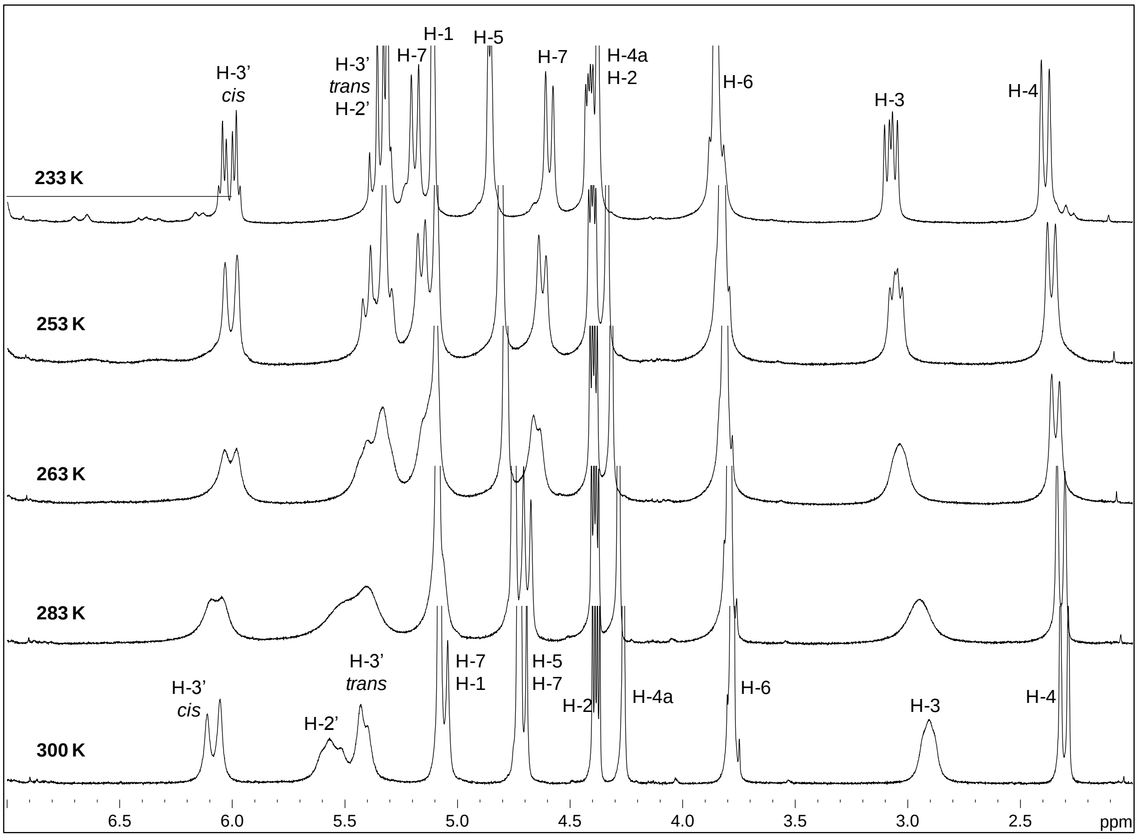

Variable temperature ^{1}H NMR (300 MHz, $CDCl_3$) Spectra of **6b**

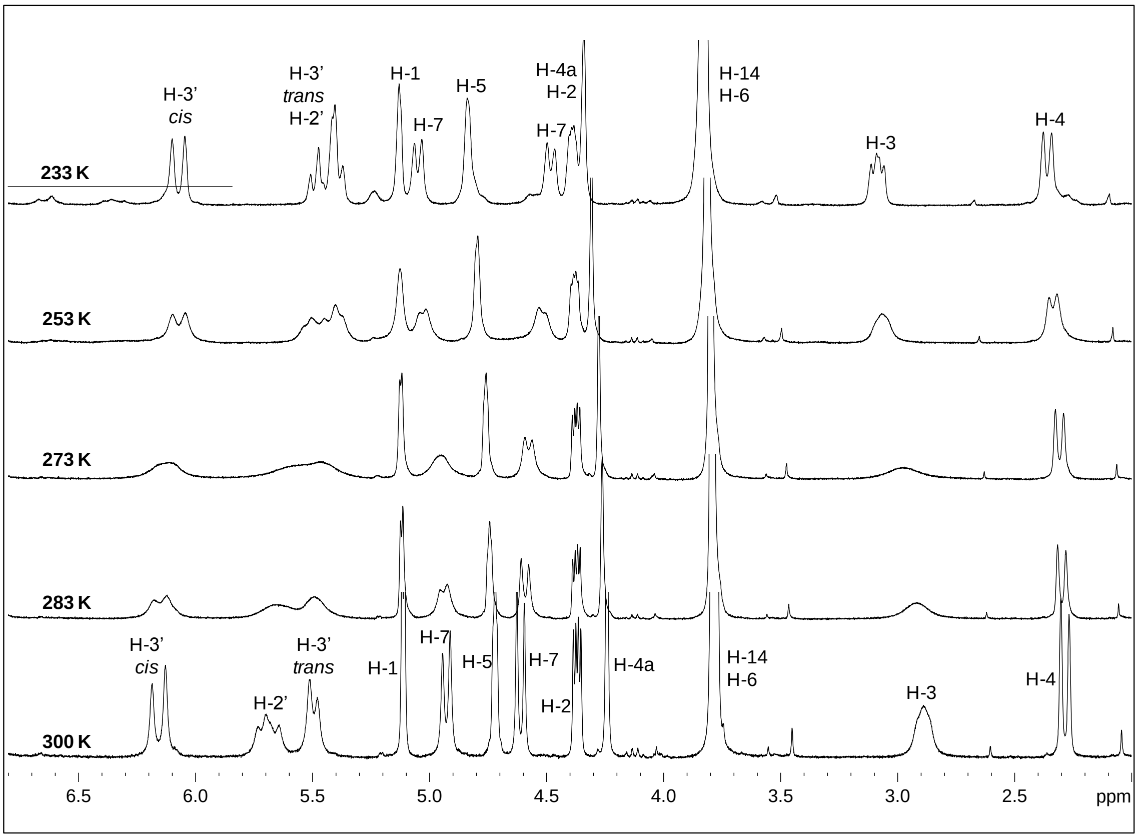

^{1}H NMR (300 MHz, $CDCl_3$) Spectra of **6a** and **6b** at 233 K.

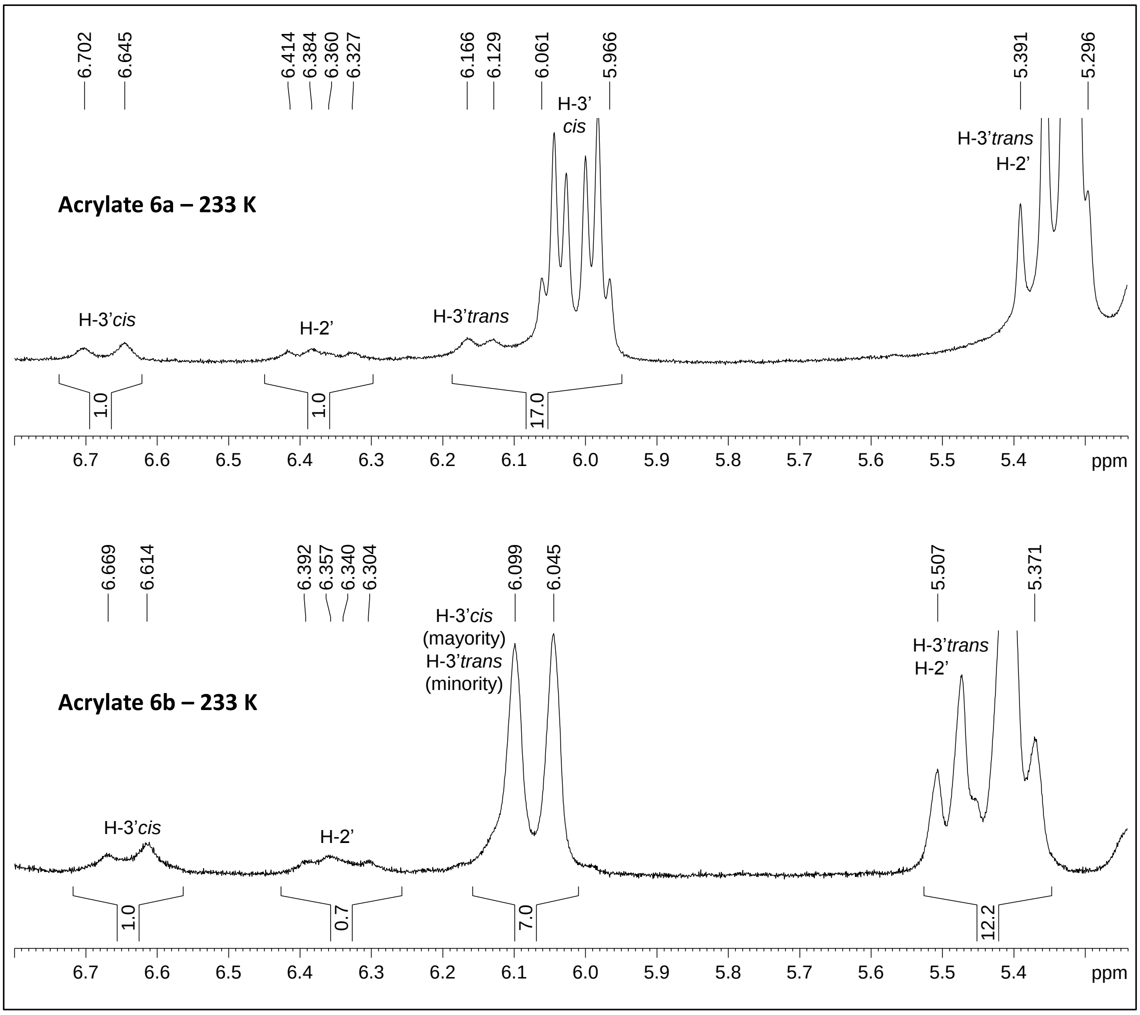

^{1}H NMR (300 MHz, $CDCl_3$) Spectra of **6a** with Et_2AlCl recorded at 300 K

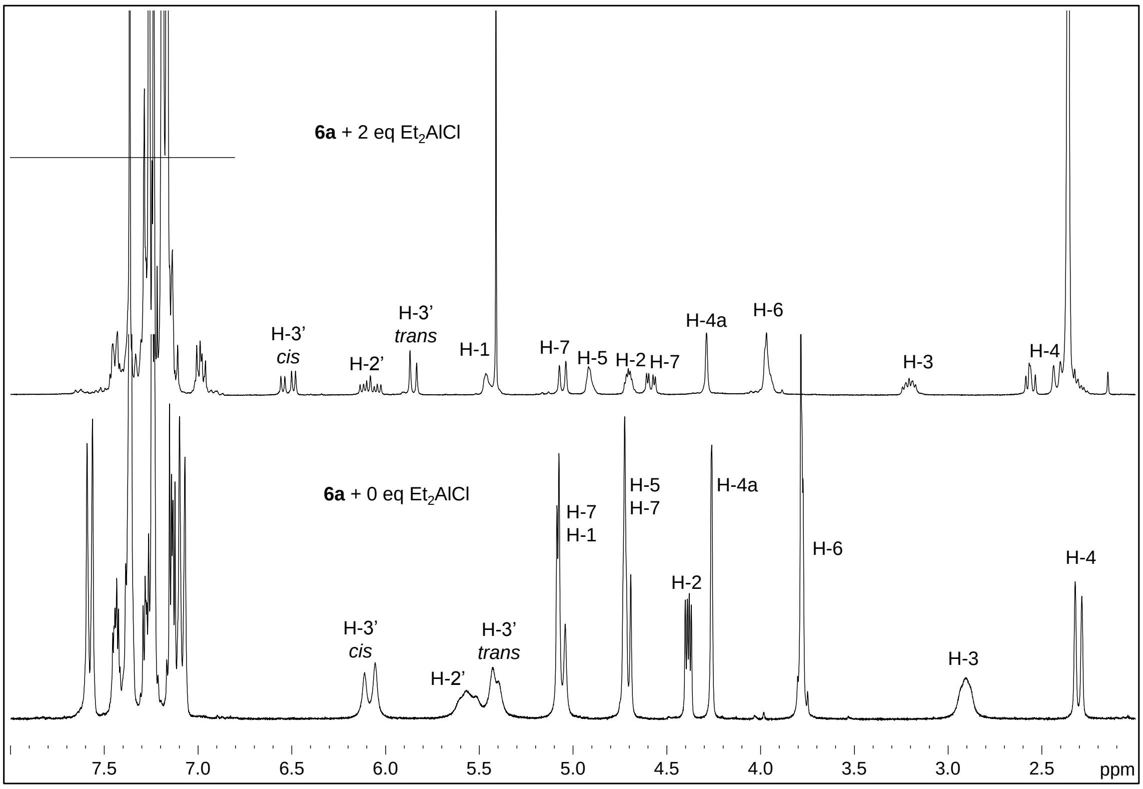

^{1}H NMR (300 MHz, $CDCl_3$) Spectra of **6b** with Et_2AlCl recorded at 300 K

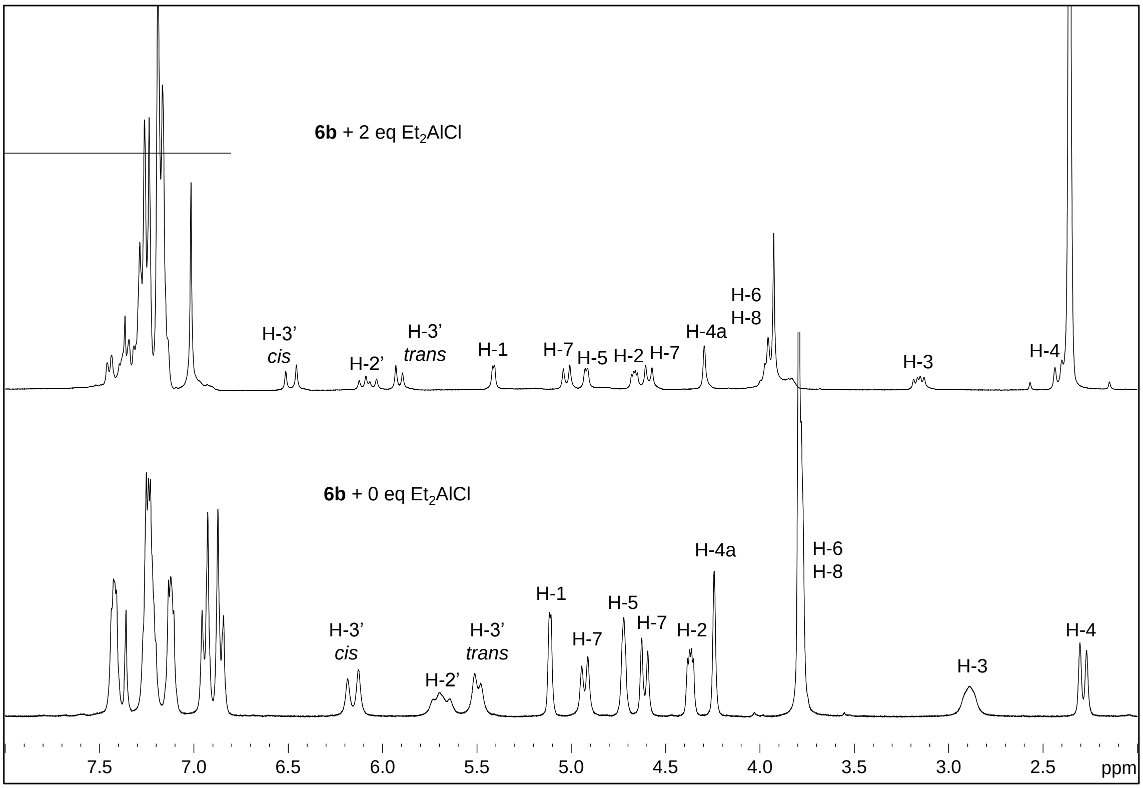

Computational methods

All molecular mechanics calculations were performed using Hyperchem with the MM+ force field [4] and the quantum mechanical calculations were performed using Gaussian 09 [5]. Conformational searches were conducted by systematic variation of all the flexible torsional angles of the studied molecules (ϕ1-ϕ7), using the MM+ force field in gas phase, with the number of steps large enough to find all low-energy conformers at least 10 times. All conformers within 10 kcal/mol of the lowest energy conformer were subjected to further reoptimization at the HF/3-21G level of theory. With the most stable conformers in hand (up to 10 kcal/mol of the lowest energy conformer) were then further optimized at the M06-2X/6-31+G(d) level of theory [6]. Geometries for all structures were fully optimized and normal coordinate analyses were used to confirm the nature of the stationary points. All transition structures were confirmed to have only one imaginary frequency. Intrinsic Reaction Coordinate (IRC) calculations were performed to determine the connections between stationary points. The electronic structures of TSs and ground states were analyzed in terms of the Wiberg bond indices (WBI) and the natural charges obtained from the Natural Bond Orbital (NBO) program as implemented in Gaussian 09 [7]. Reported thermochemical properties include zero-point energies (ZPEs) without scaling and were calculated at 1 atm and 298.15 K.

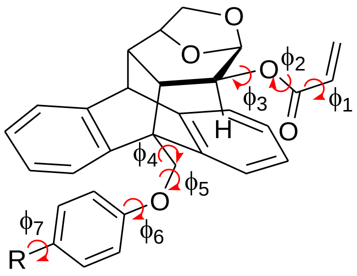

[4] Hyperchem Professional Release 7.52, Hypercube, Inc., 2005.
[5] Frisch, M. J.; Trucks, G. W.; Schlegel, H. B.; Scuseria, G. E.; Robb, M. A.; Cheeseman, J. R.; Scalmani, G.; Barone, V.; Mennucci, B.; Petersson, G. A.; Nakatsuji, H.; Caricato, M.; Li, X.; Hratchian, H. P.; Izmaylov, A. F.; Bloino, J.; Zheng, G.; Sonnenberg, J. L.; Hada, M.; Ehara, M.; Toyota, K.; Fukuda, R.; Hasegawa, J.; Ishida, M.; Nakajima, T.; Honda, Y. Kitao, O.; Nakai, H. Vreven, T.; Montgomery, J. A., Jr.,; Peralta, J. E.; Ogliaro, F.; Bearpark, M.; Heyd, J. J.; Brothers, E.; Kudin, K. N.; Staroverov, V. N.; Kobayashi, R.; Normand, J.; Raghavachari, K.; Rendell, A.; Burant, J. C.; Iyengar, S. S.; Tomasi, J.; Cossi, M.; Rega, N.; Millam, J. M.; Klene, M.; Knox, J. E.; Cross, J. B.; Bakken, V.; Adamo, C.; Jaramillo, J.; Gomperts, R.; Stratmann, R. E.; Yazyev, O.; Austin, A. J.; Cammi, R.; Pomelli, C.; Ochterski, J. W.; Martin, R. L.; Morokuma, K.; Zakrzewski, V. G.; Voth, G. A.; Salvador, P.; Dannenberg, J. J.; Dapprich, S.; Daniels, A. D.; Farkas, O.; Foresman, J. B.; Ortiz, J. V.; Cioslowski, J. and Fox, D. J. Gaussian 09, Gaussian, Inc., Wallingford CT, 2009.
[6] Zhao, Y.; Truhlar, D. G. *Acc. Chem. Res.* **2008**, *41*, 157.

Figure S4: M06-2X/6-31+G(d) optimized structures of all significant confomers found for **6a.**

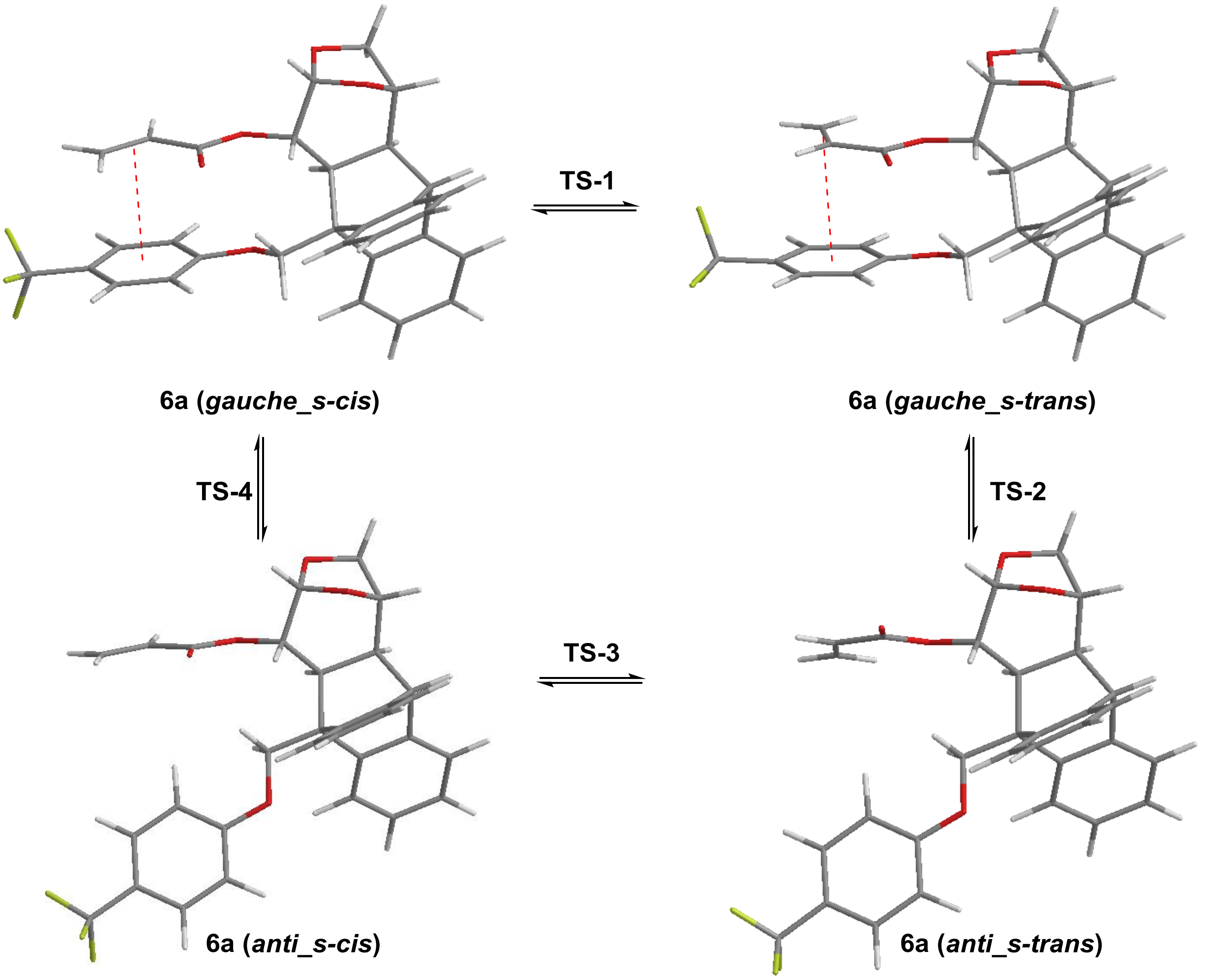

Table S3: M06-2X/6-31+G(d) energies (E), zero-point corrected energies (ZPE), enthalpies (H) and Gibbs free energies (G), in au, computed for all conformers and TSs shown in Figure S4.

	E	ZPE	H	G
**6a (*gauche_s-cis*)**	-1871.341916	-1870.833110	-1870.802151	-1870.894434
**6a (*gauche_s-trans*)**	-1871.341334	-1870.833426	-1870.802281	-1870.895305
**6a (*anti_s-cis*)**	-1871.335998	-1870.828134	-1870.796658	-1870.892300
**6a (*anti_s-trans*)**	-1871.335618	-1870.827189	-1870.795823	-1870.890606
**TS-1**	-1871.329727	-1870.822050	-1870.791409	-1870.883471
**TS-2**	-1871.314336	-1870.806503	-1870.775642	-1870.869952
**TS-3**	-1871.324904	-1870.817155	-1870.786265	-1870.880418
**TS-4**	-1871.315218	-1870.807410	-1870.776499	-1870.870974

[7] NBO Version 3.1, Glendening, E. D.; Reed, A. E.; Carpenter, J. E.; Weinhold, F. For some original literature references, see: (a) Reed, A. E.; Weinstock, R. B.; Weinhold, F. *J. Chem. Phys.* **1985**, *83*, 735-746. (b) Reed, A. E.; Curtiss, L. A.; Weinhold, F. *Chem. Rev.* **1988**, *88*, 899-926.

Figure S5: M06-2X/6-31+G(d) optimized structures of all significant confomers found for **6b.**

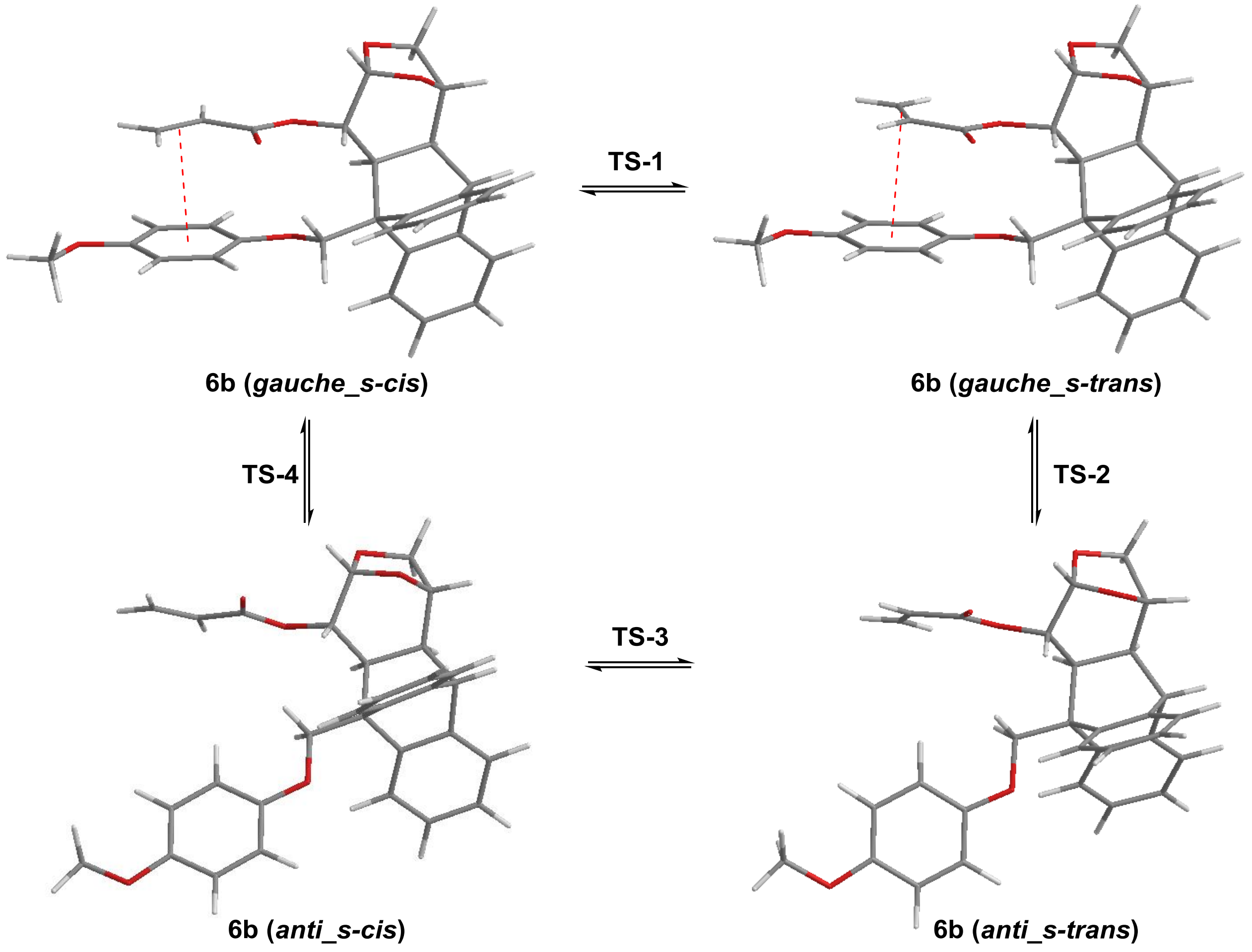

Table S4: M06-2X/6-31+G(d) energies (E), zero-point corrected energies (ZPE), enthalpies (H) and Gibbs free energies (G), in au, computed for all conformers and TSs shown in Figure S5.

	E	ZPE	H	G
**6b (*gauche_s-cis*)**	-1648.862528	-1648.326291	-1648.296249	-1648.385302
**6b (*gauche_s-trans*)**	-1648.861459	-1648.325479	-1648.295320	-1648.385481
**6b (*anti_s-cis*)**	-1648.856708	-1648.320816	-1648.290372	-1648.382948
**6b (*anti_s-trans*)**	-1648.856335	-1648.319915	-1648.289628	-1648.381144
**TS-1**	-1648.851236	-1648.315309	-1648.285806	-1648.374129
**TS-2**	-1648.835204	-1648.298895	-1648.269152	-1648.359656
**TS-3**	-1648.845784	-1648.310230	-1648.280373	-1648.371443
**TS-4**	-1648.835727	-1648.299895	-1648.270039	-1648.361170

Figure S6: M06-2X/6-31+G(d) optimized structures of all significant confomers found for **6c.**

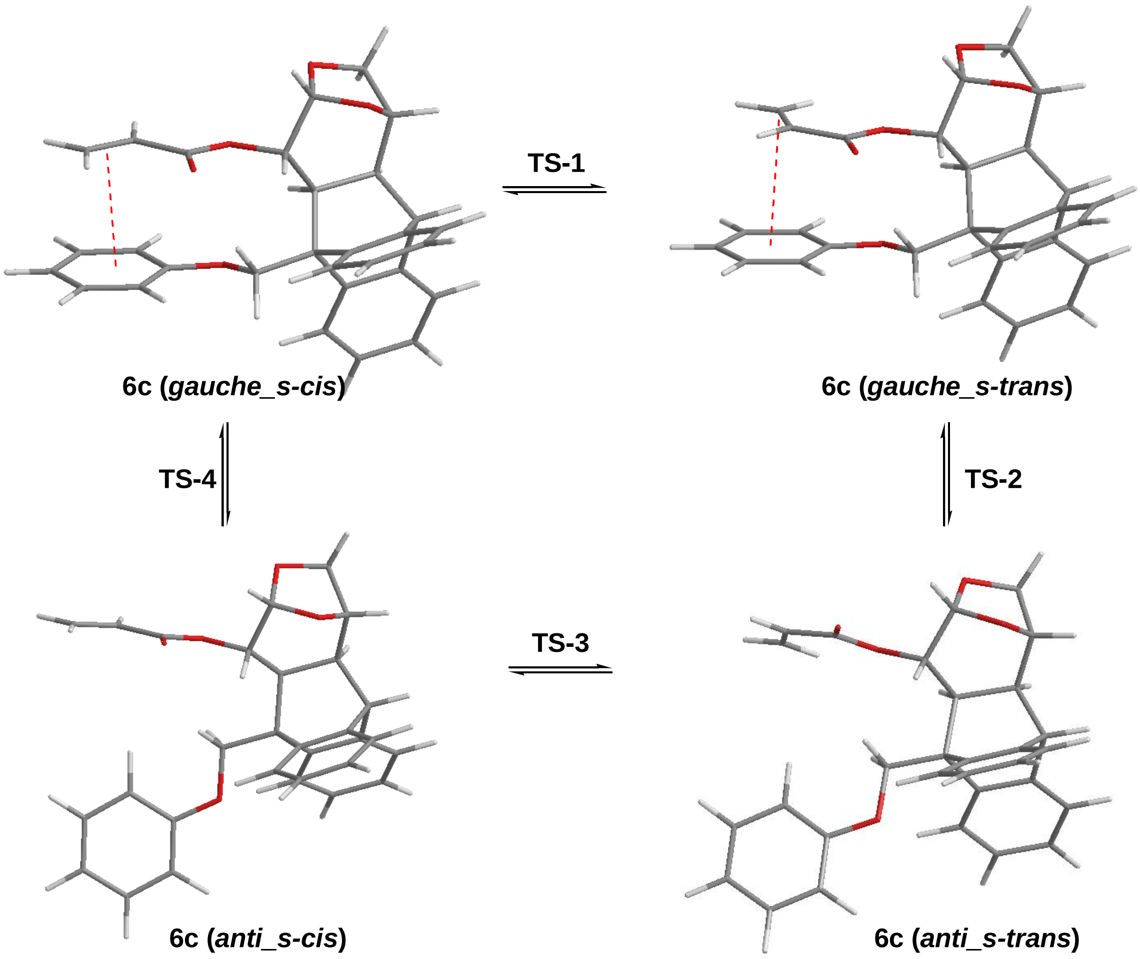

Table S5: M06-2X/6-31+G(d) energies (E), zero-point corrected energies (ZPE), enthalpies (H) and Gibbs free energies (G), in au, computed for all conformers and TSs shown in Figure S6.

	E	ZPE	H	G
**6c (*gauche_s-cis*)**	-1534.383100	-1533.880114	-1533.852627	-1533.935988
**6c (*gauche_s-trans*)**	-1534.382431	-1533.879514	-1533.852020	-1533.935554
**6c (*anti_s-cis*)**	-1534.377968	-1533.875133	-1533.847231	-1533.934146
**6c (*anti_s-trans*)**	-1534.377728	-1533.874306	-1533.846539	-1533.932458
**TS-1**	-1534.372117	-1533.870069	-1533.843033	-1533.926010
**TS-2**	-1534.356294	-1533.853331	-1533.826126	-1533.910686
**TS-3**	-1534.366950	-1533.865006	-1533.837642	-1533.923405
**TS-4**	-1534.356870	-1533.854269	-1533.826973	-1533.912063

Figure S7: M06-2X/6-31+G(d) optimized structures of all significant confomers found for **6d**.

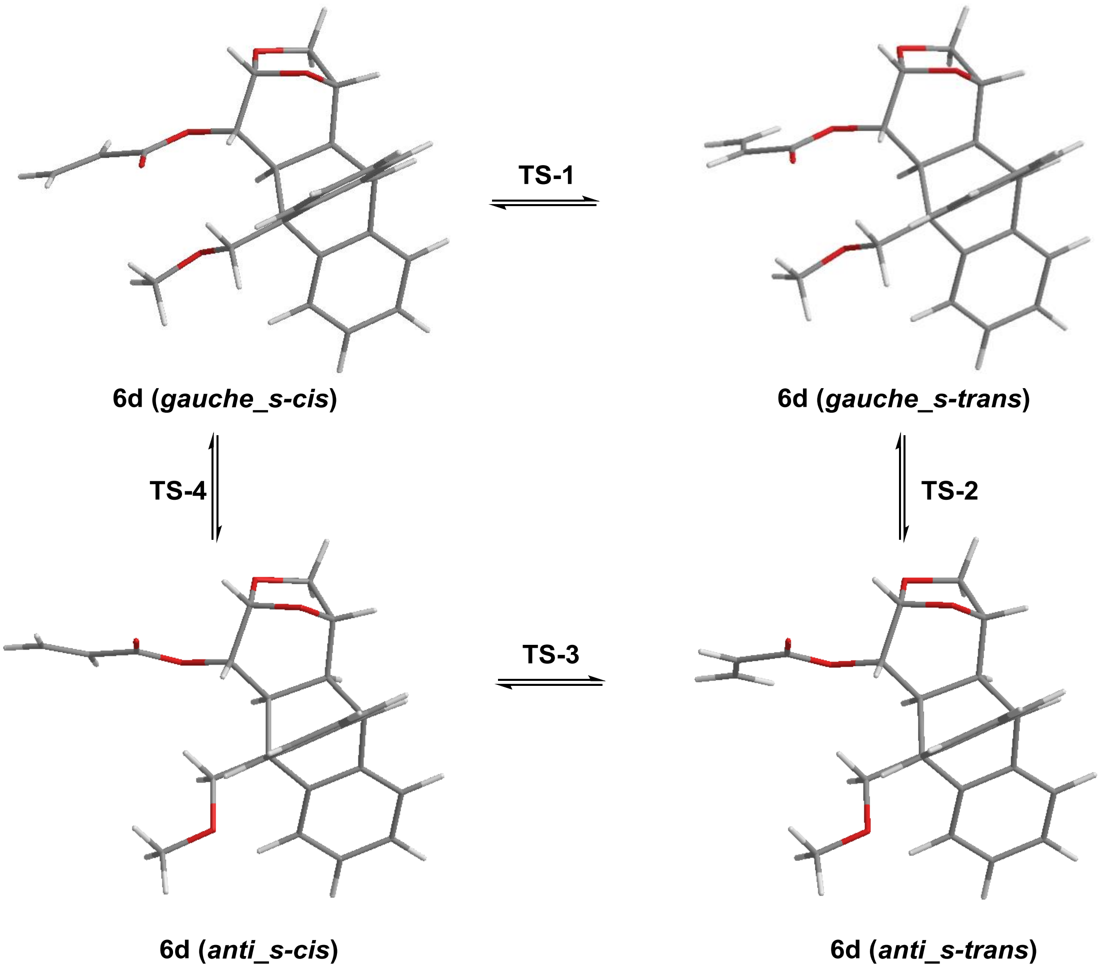

Table S6: M06-2X/6-31+G(d) energies (E), zero-point corrected energies (ZPE), enthalpies (H) and Gibbs free energies (G), in au, computed for all conformers and TSs shown in Figure S7.

	E	ZPE	H	G
**6d (*gauche_s-cis*)**	-1342.704114	-1342.253956	-1342.229463	-1342.306040
**6d (*gauche_s-trans*)**	-1342.703515	-1342.253534	-1342.228972	-1342.305964
**6d (*anti_s-cis*)**	-1342.701588	-1342.252344	-1342.227231	-1342.307250
**6d (*anti_s-trans*)**	-1342.700862	-1342.251337	-1342.226383	-1342.305322
**TS-1**	-1342.692291	-1342.242705	-1342.218793	-1342.294068
**TS-2**	-1342.680826	-1342.231406	-1342.207045	-1342.284997
**TS-3**	-1342.690771	-1342.242428	-1342.217891	-1342.296432
**TS-4**	-1342.681326	-1342.231998	-1342.207659	-1342.285516

Figure S8: M06-2X/6-31+G(d) optimized structures of the complexes between methyl acrylate (**7**) and 4-CF_3-anisol (**8**), 4-OMe-anisol (**9**) and anisol (**10**).

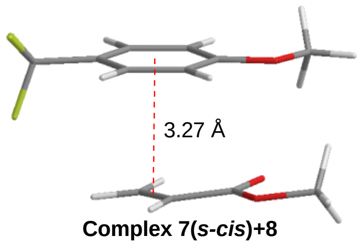

Complex 7(*s-cis*)+8
ΔE = -7.23 kcal/mol (M06-2X/6-31+G*)
ΔE = -12.02 kcal/mol (MP2/6-31+G*)

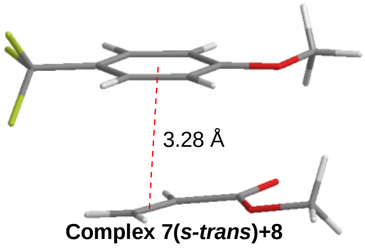

Complex 7(*s-trans*)+8
ΔE = -7.21 kcal/mol (M06-2X/6-31+G*)
ΔE = -11.99 kcal/mol (MP2/6-31+G*)

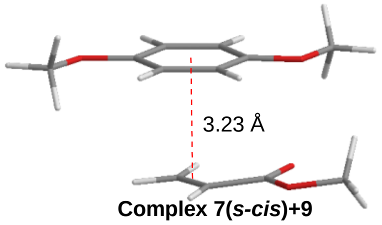

Complex 7(*s-cis*)+9
ΔE = -6.87 kcal/mol (M06-2X/6-31+G*)
ΔE = -11.53 kcal/mol (MP2/6-31+G*)

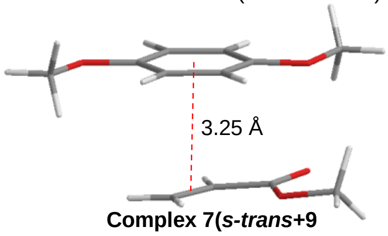

Complex 7(*s-trans*+9
ΔE = -6.63 kcal/mol (M06-2X/6-31+G*)
ΔE = -11.59 kcal/mol (MP2/6-31+G*)

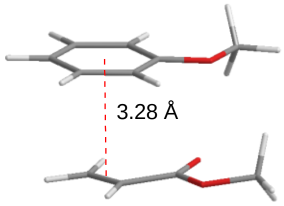

Complex 7(*s-cis*)+10
ΔE = -6.53 kcal/mol (M06-2X/6-31+G*)
ΔE = -10.66 kcal/mol (MP2/6-31+G*)

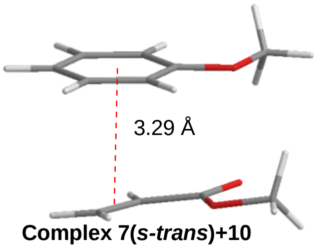

Complex 7(*s-trans*)+10
ΔE = -6.55 kcal/mol (M06-2X/6-31+G*)
ΔE = -10.79 kcal/mol (MP2/6-31+G*)

Table S7: M06-2X/6-31+G(d) energies (E), zero-point corrected energies (ZPE), enthalpies (H) and Gibbs free energies (G), in au, computed for **7**, **8, 9** and **10**, and the complexes shown in Figure S8.

	E	ZPE	H	G
**7 (*s-cis*)**	-306.342572	-306.245937	-306.238367	-306.276441
**7 (*s-trans*)**	-306.341745	-306.245130	-306.237535	-306.275752
**8**	-683.583774	-683.443798	-683.432487	-683.481244
**9**	-461.103674	-460.935669	-460.925481	-460.969892
**10**	-346.625504	-346.490575	-346.482901	-346.521412
**Complex 7(*s-cis*)+8**	-989.939973	-989.701257	-989.682150	-989.748529
**Complex 7(*s-trans*)+8**	-989.939149	-989.700420	-989.681226	-989.748091
**Complex 7(*s-cis*)+9**	-767.459053	-767.192558	-767.174267	-767.238402
**Complex 7(*s-trans*)+9**	-767.458276	-767.191369	-767.173199	-767.236984
**Complex 7(*s-cis*)+10**	-652.980393	-652.746911	-652.731343	-652.788875
**Complex 7(*s-trans*)+10**	-652.979564	-652.746151	-652.730366	-652.788875

6a (*gauche_s-cis*)

M06-2X/6-31+G(d) Geometry

C 0	-3.706113	-1.150348	-1.894796
C 0	-2.230179	-1.089794	-1.366494
C 0	-1.661985	-2.423344	-0.837870
C 0	-4.238616	-2.579076	-2.046418
O 0	-3.803781	-3.411682	-0.972305
C 0	-2.456743	-3.642222	-1.296832
C 0	-3.611829	-3.306872	-3.240139
O 0	-2.392206	-3.805611	-2.699917
C 0	-2.137492	0.018462	-0.243638
C 0	-3.072585	-0.448120	0.868643
C 0	-4.397215	-0.618601	0.443714
C 0	-4.612142	-0.245735	-1.002620
C 0	-4.079082	1.155495	-1.200601
C 0	-2.737095	1.293579	-0.821808
C 0	-2.093282	2.515551	-0.986394
C 0	-2.798694	3.599442	-1.516776
C 0	-4.132971	3.459580	-1.889607
C 0	-4.777542	2.229195	-1.736552
C 0	-5.365385	-1.092155	1.318468
C 0	-5.013713	-1.395145	2.635847
C 0	-3.700084	-1.225871	3.063087
C 0	-2.723135	-0.757100	2.179880
O 0	-0.313937	-2.560385	-1.308083
C 0	0.655588	-2.871580	-0.425577
O 0	0.476358	-2.981707	0.768336
C 0	1.957577	-3.037692	-1.119202
C 0	3.065480	-3.265137	-0.415376
C 0	-0.712826	0.183410	0.257930
O 0	0.135106	0.444282	-0.851718
C 0	1.471247	0.288831	-0.681578
C 0	2.085644	0.100704	0.558444
C 0	3.465566	-0.085101	0.616684
C 0	4.226391	-0.074489	-0.545994
C 0	3.612197	0.140549	-1.783367
C 0	2.241735	0.318791	-1.853161
C 0	5.710096	-0.272778	-0.490209
F 0	6.107439	-0.819229	0.672346
F 0	6.389039	0.883741	-0.629816
F 0	6.143156	-1.083821	-1.477364
H 0	-3.725484	-0.697570	-2.893026
H 0	-1.580835	-0.780753	-2.189923
H 0	-1.661564	-2.448006	0.255439
H 0	-5.332172	-2.598108	-2.058338
H 0	-2.112306	-4.555985	-0.805307
H 0	-3.391208	-2.651032	-4.086755
H 0	-4.235369	-4.144792	-3.571319
H 0	-5.662305	-0.332124	-1.303268
H 0	-1.047066	2.630087	-0.717775
H 0	-2.299440	4.555985	-1.640880
H 0	-4.673954	4.307146	-2.300564
H 0	-5.818988	2.112375	-2.027994
H 0	-6.389039	-1.226066	0.976089
H 0	-5.765632	-1.765595	3.326346
H 0	-3.427110	-1.464595	4.086755
H 0	-1.700539	-0.647618	2.531796
H 0	1.951076	-2.947516	-2.200797
H 0	4.031883	-3.378931	-0.896984
H 0	3.020690	-3.336871	0.668433
H 0	-0.382090	-0.731833	0.762589
H 0	-0.656714	1.013630	0.978158
H 0	1.507695	0.076164	1.474604
H 0	3.943780	-0.251427	1.576871
H 0	4.209002	0.147409	-2.691010
H 0	1.736554	0.467391	-2.802221

Number of imaginary frequencies: 0

TS-1 [6a (*gauche_s-cis*)] ⇋ [6a (*gauche_s-trans*)]

M06-2X/6-31+G(d) Geometry

C 0	-3.624349	-0.878462	-1.935025
C 0	-2.167707	-0.779634	-1.362944
C 0	-1.605190	-2.084148	-0.765821
C 0	-4.128292	-2.319917	-2.061660
O 0	-3.722628	-3.112650	-0.946498
C 0	-2.361095	-3.331546	-1.213059
C 0	-3.444105	-3.073234	-3.208041
O 0	-2.242485	-3.543680	-2.605169
C 0	-2.125658	0.365567	-0.273964
C 0	-3.087012	-0.077431	0.824263
C 0	-4.395227	-0.282084	0.364981
C 0	-4.570345	0.039392	-1.099128
C 0	-4.050886	1.441728	-1.326396
C 0	-2.724206	1.613528	-0.909282
C 0	-2.094958	2.841032	-1.088975
C 0	-2.798899	3.896007	-1.676725
C 0	-4.116731	3.721859	-2.090833
C 0	-4.747168	2.486452	-1.919738
C 0	-5.382832	-0.739771	1.226376
C 0	-5.067193	-0.992097	2.563543
C 0	-3.769599	-0.789479	3.024139
C 0	-2.772997	-0.336313	2.154924
O 0	-0.231187	-2.214254	-1.169374
C 0	0.673869	-2.584683	-0.244521
O 0	0.423582	-2.719715	0.931162
C 0	2.027066	-2.787306	-0.851538
C 0	2.438982	-3.978448	-1.272142
C 0	-0.715580	0.558672	0.256686
O 0	0.143829	0.825942	-0.841574
C 0	1.477870	0.676547	-0.665834
C 0	2.088542	0.462982	0.572434
C 0	3.472530	0.295520	0.635088
C 0	4.239157	0.349628	-0.521862
C 0	3.626044	0.579447	-1.759121
C 0	2.254167	0.741954	-1.832737
C 0	5.718101	0.111601	-0.480998
F 0	6.212645	0.160942	0.766424
F 0	6.393779	1.014985	-1.216249
F 0	6.042870	-1.099146	-0.983093
H 0	-3.618676	-0.456429	-2.946795
H 0	-1.494393	-0.488915	-2.173882
H 0	-1.656582	-2.071435	0.326275
H 0	-5.220078	-2.357755	-2.114985
H 0	-2.021574	-4.221619	-0.676244
H 0	-3.195839	-2.438458	-4.062921
H 0	-4.044274	-3.927310	-3.540769
H 0	-5.609341	-0.072208	-1.428832
H 0	-1.060749	2.981793	-0.788586
H 0	-2.310832	4.856567	-1.813532
H 0	-4.655988	4.546399	-2.548108
H 0	-5.776218	2.343387	-2.241881
H 0	-6.393779	-0.900135	0.858541
H 0	-5.834855	-1.349530	3.243500
H 0	-3.524531	-0.989961	4.062921
H 0	-1.762793	-0.199907	2.532550

H 0 2.665375 -1.907603 -0.903418
H 0 3.435070 -4.110171 -1.682589
H 0 1.800611 -4.856567 -1.221653
H 0 -0.383019 -0.349714 0.774122
H 0 -0.682637 1.394430 0.971683
H 0 1.507806 0.415329 1.486074
H 0 3.947635 0.119267 1.594698
H 0 4.228129 0.619940 -2.662456
H 0 1.752587 0.905624 -2.780864
Number of imaginary frequencies: 1 (-120.3)

6a (*gauche_s-trans*)
M06-2X/6-31+G(d) Geometry
C 0 -3.512606 -1.225082 -1.911056
C 0 -2.086301 -1.092807 -1.273502
C 0 -1.508036 -2.396124 -0.687571
C 0 -3.953092 -2.677849 -2.119387
O 0 -3.563660 -3.503821 -1.023304
C 0 -2.182476 -3.649921 -1.232461
C 0 -3.188703 -3.352716 -3.263094
O 0 -1.987491 -3.780001 -2.628124
C 0 -2.130021 0.023138 -0.154408
C 0 -3.111969 -0.491391 0.893958
C 0 -4.392081 -0.737814 0.377676
C 0 -4.527418 -0.377865 -1.081784
C 0 -4.061324 1.051537 -1.244882
C 0 -2.758930 1.264755 -0.773987
C 0 -2.178818 2.523953 -0.889125
C 0 -2.907120 3.567514 -1.467500
C 0 -4.200483 3.351719 -1.935656
C 0 -4.781398 2.085213 -1.829025
C 0 -5.389625 -1.264480 1.186415
C 0 -5.113449 -1.543045 2.527170
C 0 -3.844373 -1.298253 3.043911
C 0 -2.836350 -0.777430 2.227271
O 0 -0.108848 -2.457003 -1.000792
C 0 0.746683 -2.832075 -0.027475
O 0 0.419360 -2.996411 1.128762
C 0 2.137193 -3.005246 -0.511795
C 0 2.498906 -2.871793 -1.787473
C 0 -0.745157 0.258578 0.425715
O 0 0.125610 0.608462 -0.640702
C 0 1.457949 0.415877 -0.490402
C 0 2.076840 0.102588 0.721165
C 0 3.453583 -0.122398 0.747734
C 0 4.204892 -0.018346 -0.414897
C 0 3.586072 0.326559 -1.621002
C 0 2.220703 0.545568 -1.659473
C 0 5.666616 -0.342184 -0.413854
F 0 6.197064 -0.329726 0.819928
F 0 6.377963 0.519002 -1.165263
F 0 5.905406 -1.571836 -0.924998
H 0 -3.481739 -0.761558 -2.904116
H 0 -1.388349 -0.757108 -2.045585
H 0 -1.630388 -2.429344 0.398302
H 0 -5.039105 -2.755119 -2.223017
H 0 -1.828367 -4.551929 -0.726053
H 0 -2.941607 -2.675573 -4.085257
H 0 -3.730585 -4.223878 -3.648147
H 0 -5.548141 -0.522093 -1.453154
H 0 -1.163154 2.697922 -0.546139
H 0 -2.456628 4.551929 -1.555110
H 0 -4.759002 4.167728 -2.385131
H 0 -5.791233 1.909709 -2.193271
H 0 -6.377963 -1.457338 0.775133
H 0 -5.889648 -1.953062 3.166583
H 0 -3.629778 -1.519332 4.085257
H 0 -1.846313 -0.612246 2.644682
H 0 2.847669 -3.248231 0.272658
H 0 3.534749 -3.000237 -2.087794
H 0 1.770364 -2.628007 -2.555206
H 0 -0.388499 -0.653295 0.918904
H 0 -0.768100 1.067755 1.170818
H 0 1.507455 0.019699 1.639956
H 0 3.935980 -0.379377 1.685734
H 0 4.177477 0.413652 -2.528453
H 0 1.711984 0.799904 -2.583880
Number of imaginary frequencies: 0

TS-2 [6a (*gauche_s-trans*)] ⇋ [6a (*anti_s-trans*)]
M06-2X/6-31+G(d) Geometry
C 0 -4.320733 -0.443061 -2.003354
C 0 -2.836548 -0.759830 -1.626353
C 0 -2.644090 -2.124814 -0.909320
C 0 -5.262289 -1.643369 -1.904287
O 0 -5.003625 -2.393880 -0.720165
C 0 -3.829936 -3.078533 -1.059943
C 0 -4.995390 -2.696347 -2.986005
O 0 -3.973229 -3.498130 -2.403128
C 0 -2.235329 0.425403 -0.733458
C 0 -3.089415 0.397541 0.551574
C 0 -4.464983 0.525033 0.300768
C 0 -4.801527 0.759714 -1.147583
C 0 -3.969425 1.921464 -1.618579
C 0 -2.583994 1.757328 -1.436673
C 0 -1.746568 2.789969 -1.868238
C 0 -2.283912 3.934307 -2.465264
C 0 -3.654485 4.074484 -2.646615
C 0 -4.502587 3.054136 -2.218857
C 0 -5.393516 0.469090 1.330830
C 0 -4.959063 0.287651 2.643805
C 0 -3.598609 0.167451 2.906005
C 0 -2.665113 0.215796 1.866423
O 0 -1.513453 -2.815034 -1.468986
C 0 -0.555871 -3.269523 -0.637770
O 0 -0.521333 -3.031110 0.550430
C 0 0.481801 -4.062261 -1.341213
C 0 0.407753 -4.384652 -2.632482
C 0 -0.748719 0.079470 -0.462673
O 0 0.184419 1.006512 -0.992069
C 0 1.496254 0.674434 -0.886280
C 0 1.970180 -0.461877 -0.219030
C 0 3.341793 -0.699352 -0.176486
C 0 4.234251 0.175622 -0.786909
C 0 3.758158 1.308027 -1.449249
C 0 2.395559 1.556322 -1.498206
C 0 5.699169 -0.134658 -0.730029
F 0 6.453663 0.847367 -1.251004
F 0 6.001396 -1.263089 -1.405985
F 0 6.123055 -0.332980 0.533998
H 0 -4.344865 -0.105083 -3.046168
H 0 -2.243603 -0.801287 -2.546621
H 0 -2.476208 -1.982030 0.161910
H 0 -6.306391 -1.320121 -1.871648
H 0 -3.715472 -3.955102 -0.417091

H 0 -4.633620 -2.268006 -3.925442
H 0 -5.878739 -3.315283 -3.176740
H 0 -5.871665 0.940974 -1.297967
H 0 -0.678194 2.726506 -1.741946
H 0 -1.610702 4.724196 -2.786283
H 0 -4.061816 4.969322 -3.108084
H 0 -5.579962 3.140665 -2.341820
H 0 -6.453663 0.567825 1.108450
H 0 -5.678879 0.242792 3.455743
H 0 -3.250087 0.030888 3.925442
H 0 -1.616325 0.108775 2.121254
H 0 1.308942 -4.368175 -0.707962
H 0 1.189596 -4.969322 -3.107225
H 0 -0.438255 -4.076416 -3.239217
H 0 -0.523449 -0.883459 -0.920303
H 0 -0.560664 -0.022562 0.608089
H 0 1.297754 -1.157687 0.270693
H 0 3.715035 -1.577758 0.343451
H 0 4.453408 1.993028 -1.923424
H 0 2.002797 2.430135 -2.008457
Number of imaginary frequencies: 1 (-143.7)

6a (*anti_s-trans*)
M06-2X/6-31+G(d) Geometry
O 0 1.350149 3.810156 0.385747
O 0 1.830763 2.625891 -1.470703
C 0 1.163996 3.574578 -0.786226
C 0 0.152375 4.281218 -1.613346
C 0 -0.102948 3.979085 -2.886368
C 0 2.731985 1.795803 -0.721103
C 0 4.072550 2.489407 -0.502567
O 0 4.680657 2.839309 -1.731694
C 0 5.691190 1.869195 -1.993502
C 0 5.367547 0.765391 -0.980291
C 0 4.273912 -0.177304 -1.495540
C 0 2.849701 0.474610 -1.501371
O 0 4.912275 1.550298 0.119977
C 0 1.797631 -0.574699 -0.957714
C 0 4.203070 -1.548296 -0.752933
C 0 0.399583 0.007487 -1.081575
C 0 2.008658 -1.849522 -1.766356
C 0 3.294943 -2.389000 -1.623106
C 0 3.660254 -3.539723 -2.309477
C 0 2.733229 -4.163160 -3.148378
C 0 1.458630 -3.623650 -3.302090
C 0 1.091216 -2.464995 -2.612134
C 0 2.218884 -0.865365 0.478614
C 0 3.527953 -1.353852 0.582070
C 0 4.092674 -1.604831 1.825786
C 0 3.343958 -1.369113 2.980175
C 0 2.041910 -0.884415 2.880795
C 0 1.474644 -0.628383 1.629789
O 0 -0.541582 -0.957564 -0.644020
C 0 -1.854321 -0.617452 -0.667392
C 0 -2.743402 -1.613488 -0.238262
C 0 -4.104375 -1.366048 -0.227388
C 0 -4.592523 -0.122110 -0.641702
C 0 -3.712246 0.864430 -1.064726
C 0 -2.337351 0.626691 -1.080476
C 0 -6.072406 0.112193 -0.664609
F 0 -6.381576 1.418493 -0.731023
F 0 -6.678152 -0.388728 0.428616
F 0 -6.661262 -0.478542 -1.726252
H 0 -0.375951 5.067784 -1.083440
H 0 0.443289 3.192666 -3.398858
H 0 -0.859519 4.518766 -3.447781
H 0 2.312400 1.633796 0.277440
H 0 3.975055 3.372808 0.131403
H 0 6.678152 2.307257 -1.807710
H 0 5.618809 1.545832 -3.035369
H 0 6.249694 0.209947 -0.649846
H 0 4.535544 -0.418381 -2.532246
H 0 2.586103 0.703765 -2.539968
H 0 5.201496 -1.988810 -0.655455
H 0 0.303459 0.910968 -0.464377
H 0 0.196823 0.286891 -2.126734
H 0 4.660906 -3.949853 -2.192818
H 0 3.008362 -5.067784 -3.682797
H 0 0.739831 -4.107948 -3.956658
H 0 0.088221 -2.065101 -2.726350
H 0 5.111395 -1.979373 1.896310
H 0 3.778714 -1.561590 3.956658
H 0 1.461356 -0.700857 3.780118
H 0 0.455894 -0.255907 1.565137
H 0 -2.336400 -2.566619 0.083847
H 0 -4.794534 -2.134309 0.109298
H 0 -4.092153 1.830618 -1.381404
H 0 -1.667260 1.413228 -1.408935
Number of imaginary frequencies: 0

TS-3 [6a (*anti_s-trans*)] ⇋ [6a (*anti_s-cis*)]
M06-2X/6-31+G(d) Geometry
C 0 -2.797852 -2.058909 -0.172113
C 0 -1.886305 -1.099794 -0.639386
C 0 -2.343134 0.133328 -1.108794
C 0 -3.713283 0.401407 -1.109902
C 0 -4.614082 -0.547544 -0.648775
C 0 -4.152408 -1.782468 -0.177886
O 0 -0.582065 -1.465795 -0.597480
C 0 0.389091 -0.532317 -1.039932
C 0 1.763754 -1.166087 -0.915689
C 0 2.861387 -0.147667 -1.429286
C 0 4.252821 -0.868053 -1.443446
C 0 4.122723 -2.246922 -0.724751
C 0 3.453188 -2.048532 0.612448
C 0 2.166007 -1.503785 0.516054
C 0 2.806297 1.156912 -0.610845
C 0 4.182549 1.764539 -0.337654
O 0 4.973961 0.754850 0.232496
C 0 5.388972 0.006560 -0.907230
C 0 3.182975 -3.033165 -1.610243
C 0 1.923755 -2.433570 -1.749321
O 0 4.815971 2.157637 -1.540162
C 0 5.754153 1.138817 -1.875336
C 0 4.002657 -2.348584 1.851938
C 0 3.261159 -2.104903 3.009193
C 0 1.981191 -1.563073 2.917174
C 0 1.429273 -1.257916 1.670387
C 0 0.983857 -2.993704 -2.609186
C 0 1.302561 -4.156589 -3.316305
C 0 2.550315 -4.756197 -3.165222
C 0 3.500092 -4.188529 -2.312364
O 0 1.986249 2.069076 -1.366149
C 0 1.488792 3.131098 -0.707159
C 0 0.647969 3.986714 -1.602935

C 0 1.145936 5.037991 -2.245051
O 0 1.686209 3.346392 0.463989
C 0 -6.088065 -0.277809 -0.638454
F 0 -6.770730 -1.187329 -1.362659
F 0 -6.391702 0.931708 -1.138163
F 0 -6.600589 -0.331366 0.607431
H 0 -2.411849 -3.006956 0.188201
H 0 -1.653479 0.884192 -1.476116
H 0 -4.073597 1.357609 -1.474881
H 0 -4.860316 -2.522665 0.184706
H 0 0.198802 -0.253348 -2.086867
H 0 0.323141 0.377843 -0.427932
H 0 2.608398 0.124003 -2.460095
H 0 4.500509 -1.099606 -2.485793
H 0 5.101887 -2.729911 -0.633158
H 0 2.344208 0.996587 0.369243
H 0 4.123812 2.612442 0.346054
H 0 6.250261 -0.598815 -0.611519
H 0 5.635518 0.868981 -2.928465
H 0 6.770730 1.506847 -1.698319
H 0 5.004694 -2.766498 1.917640
H 0 3.684182 -2.336237 3.982415
H 0 1.406229 -1.373491 3.818825
H 0 0.427214 -0.842042 1.609805
H 0 -0.000099 -2.548396 -2.722696
H 0 0.566431 -4.597003 -3.982415
H 0 2.787255 -5.663949 -3.712401
H 0 4.481021 -4.644208 -2.197294
H 0 -0.402388 3.713085 -1.677989
H 0 0.517026 5.663949 -2.869991
H 0 2.196199 5.305786 -2.166341
Number of imaginary frequencies: 1 (-137.5)

6a (*anti_s-cis*)
M06-2X/6-31+G(d) Geometry
O 0 0.652182 3.247992 0.030767
O 0 1.839426 2.549236 -1.760529
C 0 0.858783 3.252379 -1.161126
C 0 0.055989 3.993883 -2.169345
C 0 -1.016983 4.685106 -1.787334
C 0 2.702080 1.750101 -0.935757
C 0 4.004848 2.502003 -0.685506
O 0 4.644062 2.849693 -1.898385
C 0 5.692226 1.906954 -2.106799
C 0 5.386154 0.824972 -1.064539
C 0 4.351888 -0.185423 -1.572004
C 0 2.900611 0.400201 -1.652028
O 0 4.864238 1.624631 -0.004905
C 0 1.879912 -0.670386 -1.087084
C 0 4.318871 -1.521020 -0.766158
C 0 0.464145 -0.153107 -1.275585
C 0 2.171493 -1.972190 -1.823070
C 0 3.475809 -2.443220 -1.618018
C 0 3.914150 -3.607653 -2.234719
C 0 3.041364 -4.315682 -3.064377
C 0 1.748232 -3.845775 -3.278584
C 0 1.307845 -2.672396 -2.659780
C 0 2.271232 -0.867225 0.373731
C 0 3.596752 -1.290679 0.538392
C 0 4.136269 -1.445781 1.808640
C 0 3.345617 -1.178875 2.927768
C 0 2.027872 -0.756848 2.767368
C 0 1.485628 -0.596485 1.489490
O 0 -0.455398 -1.133549 -0.823601
C 0 -1.774142 -0.816757 -0.847461
C 0 -2.644178 -1.818120 -0.390380
C 0 -4.008454 -1.593502 -0.377995
C 0 -4.521179 -0.368430 -0.820428
C 0 -3.660816 0.622509 -1.270865
C 0 -2.281797 0.407362 -1.288403
C 0 -6.003827 -0.155492 -0.790566
F 0 -6.659108 -1.067239 -1.537188
F 0 -6.358543 1.056754 -1.249548
F 0 -6.502778 -0.266676 0.457473
H 0 0.386698 3.935202 -3.201689
H 0 -1.303897 4.715504 -0.739304
H 0 -1.622784 5.232881 -2.502029
H 0 2.233794 1.613282 0.042912
H 0 3.842779 3.399627 -0.082966
H 0 6.659108 2.385681 -1.916463
H 0 5.653289 1.546122 -3.138201
H 0 6.282221 0.322443 -0.689656
H 0 4.658917 -0.460996 -2.587574
H 0 2.659942 0.570205 -2.707308
H 0 5.332295 -1.910778 -0.619038
H 0 0.317566 0.766935 -0.695228
H 0 0.280078 0.073031 -2.337157
H 0 4.928931 -3.963319 -2.070478
H 0 3.372868 -5.232881 -3.542667
H 0 1.071548 -4.397854 -3.924371
H 0 0.291061 -2.327116 -2.820168
H 0 5.167858 -1.769999 1.926636
H 0 3.760392 -1.298373 3.924371
H 0 1.415350 -0.546427 3.639148
H 0 0.456759 -0.265339 1.377598
H 0 -2.219554 -2.757189 -0.050237
H 0 -4.684111 -2.366872 -0.023087
H 0 -4.057687 1.572803 -1.612921
H 0 -1.629009 1.197534 -1.641133
Number of imaginary frequencies: 0

TS-4 [6a (*anti_s-cis*)] ⇋ [6a (*gauche_s-cis*)]
M06-2X/6-31+G(d) Geometry
C 0 -4.254330 -0.509664 -1.948310
C 0 -2.766046 -0.831647 -1.592774
C 0 -2.550843 -2.247988 -0.990652
C 0 -5.178502 -1.727697 -1.935298
O 0 -4.905136 -2.558619 -0.808998
C 0 -3.726180 -3.204016 -1.203454
C 0 -4.902898 -2.696658 -3.091791
O 0 -3.875477 -3.532318 -2.569480
C 0 -2.188422 0.284380 -0.599526
C 0 -3.051572 0.136904 0.670972
C 0 -4.426761 0.269347 0.420611
C 0 -4.755723 0.617651 -1.006417
C 0 -3.936161 1.823843 -1.375703
C 0 -2.550323 1.664296 -1.194184
C 0 -1.723778 2.739991 -1.531500
C 0 -2.272217 3.921620 -2.039049
C 0 -3.642971 4.057314 -2.222676
C 0 -4.480336 2.994415 -1.887441
C 0 -5.362004 0.113316 1.434209
C 0 -4.935316 -0.178975 2.729583
C 0 -3.575748 -0.308503 2.991372
C 0 -2.635113 -0.157071 1.968104

O 0 -1.413313 -2.873915 -1.611957
C 0 -0.439373 -3.368941 -0.824094
O 0 -0.416561 -3.253917 0.381987
C 0 0.613031 -4.024284 -1.641727
C 0 1.706845 -4.505517 -1.053212
C 0 -0.698942 -0.060743 -0.345288
O 0 0.226338 0.912831 -0.799816
C 0 1.539768 0.578695 -0.728358
C 0 2.023218 -0.611643 -0.178586
C 0 3.399306 -0.848877 -0.169837
C 0 4.280134 0.084184 -0.697084
C 0 3.792106 1.278169 -1.240861
C 0 2.432176 1.524464 -1.257303
C 0 5.758798 -0.155969 -0.696160
F 0 6.276921 -0.108479 -1.940579
F 0 6.084437 -1.352972 -0.179505
F 0 6.421636 0.775165 0.019244
H 0 -4.283921 -0.096744 -2.963659
H 0 -2.169860 -0.787072 -2.510573
H 0 -2.373588 -2.190546 0.086927
H 0 -6.226724 -1.422118 -1.875548
H 0 -3.596380 -4.121523 -0.623614
H 0 -4.544919 -2.197368 -3.997306
H 0 -5.781377 -3.306429 -3.328180
H 0 -5.827121 0.796155 -1.151020
H 0 -0.655825 2.681370 -1.399951
H 0 -1.607904 4.744378 -2.287886
H 0 -4.058169 4.980875 -2.615386
H 0 -5.557692 3.076064 -2.013612
H 0 -6.421636 0.218891 1.212545
H 0 -5.661137 -0.303068 3.527687
H 0 -3.233766 -0.533326 3.997306
H 0 -1.587311 -0.276689 2.221357
H 0 0.444323 -4.072974 -2.712662
H 0 2.497584 -4.980875 -1.624373
H 0 1.825535 -4.432362 0.025047
H 0 -0.459642 -0.984261 -0.870417
H 0 -0.511790 -0.238780 0.715847
H 0 1.357834 -1.356436 0.244428
H 0 3.778332 -1.773272 0.254052
H 0 4.483155 2.008527 -1.652476
H 0 2.030992 2.441014 -1.677975
Number of imaginary frequencies: 1 (-161.0)

6b (*gauche_s-cis*)
M06-2X/6-31+G(d) Geometry
C 0 -4.231495 -1.197087 -1.893736
C 0 -2.745742 -1.089986 -1.405823
C 0 -2.125565 -2.402041 -0.882751
C 0 -4.722307 -2.642260 -2.027827
O 0 -4.239723 -3.456844 -0.960317
C 0 -2.893234 -3.648241 -1.313373
C 0 -4.098231 -3.354512 -3.233094
O 0 -2.857274 -3.823664 -2.716238
C 0 -2.653597 0.028660 -0.293684
C 0 -3.543120 -0.455759 0.847478
C 0 -4.873496 -0.669744 0.460951
C 0 -5.140073 -0.315465 -0.981148
C 0 -4.655325 1.100073 -1.202988
C 0 -3.307764 1.280139 -0.863843
C 0 -2.704660 2.518906 -1.056004
C 0 -3.456992 3.577475 -1.572909
C 0 -4.796979 3.396446 -1.905456
C 0 -5.400546 2.148708 -1.725808
C 0 -5.802537 -1.164172 1.365898
C 0 -5.406189 -1.444479 2.675445
C 0 -4.086946 -1.231965 3.064894
C 0 -3.149030 -0.742386 2.150952
O 0 -0.786102 -2.497683 -1.384618
C 0 0.211332 -2.787834 -0.526827
O 0 0.058904 -2.936725 0.666686
C 0 1.505920 -2.878782 -1.246885
C 0 2.638360 -3.045706 -0.566025
C 0 -1.217875 0.238772 0.158740
O 0 -0.420411 0.520995 -0.976009
C 0 0.934555 0.404396 -0.838586
C 0 1.593981 0.259247 0.375810
C 0 2.988389 0.123633 0.407393
C 0 3.719044 0.140956 -0.776480
C 0 3.051402 0.310951 -1.997178
C 0 1.675529 0.437019 -2.028678
O 0 5.073750 -0.004257 -0.857091
C 0 5.780710 -0.190440 0.348754
H 0 -4.292481 -0.749475 -2.892691
H 0 -2.125810 -0.767029 -2.246258
H 0 -2.097621 -2.418848 0.210382
H 0 -5.814786 -2.695545 -2.016841
H 0 -2.509750 -4.546963 -0.823054
H 0 -3.909960 -2.692026 -4.082558
H 0 -4.706194 -4.207571 -3.554489
H 0 -6.195263 -0.435782 -1.251492
H 0 -1.654187 2.663590 -0.821141
H 0 -2.989293 4.546963 -1.718265
H 0 -5.374445 4.224895 -2.305779
H 0 -6.446033 1.998702 -1.986211
H 0 -6.830708 -1.332595 1.053311
H 0 -6.127692 -1.831211 3.389142
H 0 -3.778981 -1.453476 4.082558
H 0 -2.120230 -0.598945 2.471429
H 0 1.475001 -2.773946 -2.326606
H 0 3.600056 -3.094084 -1.067831
H 0 2.616799 -3.128362 0.517663
H 0 -0.843807 -0.663784 0.658239
H 0 -1.165938 1.070826 0.878831
H 0 1.047032 0.232882 1.311674
H 0 3.474900 -0.001624 1.368211
H 0 3.637707 0.326714 -2.910848
H 0 1.144115 0.554450 -2.968411
H 0 5.452207 -1.102442 0.864603
H 0 5.657842 0.669811 1.018861
H 0 6.830708 -0.287861 0.072376
Number of imaginary frequencies: 0

TS-1 [6b (*gauche_s-cis*)] ⇋ [6b (*gauche_s-trans*)]
M06-2X/6-31+G(d) Geometry
C 0 -4.171951 -0.994240 -1.939994
C 0 -2.700657 -0.847179 -1.421292
C 0 -2.083434 -2.125048 -0.821465
C 0 -4.637779 -2.451029 -2.029001
O 0 -4.177594 -3.214354 -0.914537
C 0 -2.818927 -3.401158 -1.219429
C 0 -3.966662 -3.202708 -3.184891
O 0 -2.739499 -3.640523 -2.610152
C 0 -2.650329 0.318249 -0.354828
C 0 -3.559382 -0.130249 0.784798

C 0	-4.876961	-0.380305	0.377153
C 0	-5.113849	-0.090207	-1.084631
C 0	-4.643101	1.321953	-1.353795
C 0	-3.306889	1.536798	-0.989957
C 0	-2.717833	2.776510	-1.215939
C 0	-3.472288	3.801165	-1.794599
C 0	-4.800000	3.585137	-2.154128
C 0	-5.389819	2.336633	-1.937948
C 0	-5.819636	-0.850198	1.281123
C 0	-5.449644	-1.069763	2.609887
C 0	-4.142938	-0.821607	3.019912
C 0	-3.191523	-0.355945	2.107657
O 0	-0.721349	-2.218746	-1.270693
C 0	0.221943	-2.570134	-0.378340
O 0	0.009116	-2.744771	0.799647
C 0	1.564348	-2.705021	-1.026795
C 0	2.003597	-3.871139	-1.488554
C 0	-1.226541	0.557329	0.117849
O 0	-0.416668	0.829354	-1.009095
C 0	0.936337	0.728995	-0.855840
C 0	1.583549	0.555676	0.362437
C 0	2.980928	0.442971	0.408888
C 0	3.727601	0.512463	-0.762861
C 0	3.069668	0.697327	-1.988588
C 0	1.692041	0.802451	-2.034905
O 0	5.085110	0.409313	-0.828913
C 0	5.782674	0.206352	0.380973
H 0	-4.214583	-0.587871	-2.957393
H 0	-2.061880	-0.554425	-2.258767
H 0	-2.096758	-2.094058	0.271450
H 0	-5.729192	-2.520398	-2.049505
H 0	-2.438318	-4.270763	-0.676625
H 0	-3.755775	-2.571405	-4.052518
H 0	-4.555858	-4.073391	-3.493313
H 0	-6.160486	-0.236624	-1.374594
H 0	-1.676389	2.947432	-0.959869
H 0	-3.015363	4.771415	-1.966718
H 0	-5.378580	4.386924	-2.603979
H 0	-6.426120	2.159901	-2.217663
H 0	-6.837917	-1.047011	0.953196
H 0	-6.181836	-1.437803	3.322584
H 0	-3.855330	-0.996278	4.052518
H 0	-2.172541	-0.183168	2.445003
H 0	2.165187	-1.798837	-1.076653
H 0	2.989286	-3.960607	-1.934249
H 0	1.396561	-4.771415	-1.440326
H 0	-0.853848	-0.334347	0.638985
H 0	-1.192783	1.401529	0.824595
H 0	1.028382	0.495040	1.291765
H 0	3.455923	0.296302	1.372084
H 0	3.666026	0.744672	-2.894508
H 0	1.171433	0.930159	-2.978889
H 0	5.473895	-0.729473	0.863698
H 0	5.626944	1.043136	1.073425
H 0	6.837917	0.147635	0.114511

Number of imaginary frequencies: 1 (-112.6)

6b (*gauche_s-trans*)

M06-2X/6-31+G(d) Geometry

C 0	-4.095694	-1.230892	-1.914081
C 0	-2.642833	-1.093229	-1.343297
C 0	-2.043821	-2.388579	-0.761277
C 0	-4.539298	-2.685764	-2.095108
O 0	-4.110808	-3.502578	-1.006814
C 0	-2.737446	-3.650043	-1.263472
C 0	-3.812584	-3.366748	-3.260603
O 0	-2.594810	-3.801209	-2.663449
C 0	-2.633460	0.040889	-0.242517
C 0	-3.563884	-0.454984	0.859329
C 0	-4.866612	-0.712400	0.410245
C 0	-5.072932	-0.376533	-1.046983
C 0	-4.617591	1.051403	-1.253757
C 0	-3.293201	1.271305	-0.851732
C 0	-2.718837	2.527587	-1.016717
C 0	-3.475701	3.562365	-1.573840
C 0	-4.791227	3.340695	-1.972442
C 0	-5.366269	2.076223	-1.817216
C 0	-5.823936	-1.224549	1.275324
C 0	-5.483659	-1.478129	2.606203
C 0	-4.191757	-1.222615	3.056645
C 0	-3.224936	-0.715911	2.183330
O 0	-0.657511	-2.449490	-1.124662
C 0	0.232110	-2.818418	-0.181392
O 0	-0.059088	-3.014406	0.979842
C 0	1.612643	-2.948780	-0.705480
C 0	1.938220	-2.778470	-1.986190
C 0	-1.222060	0.284208	0.266329
O 0	-0.401421	0.613462	-0.837750
C 0	0.949686	0.462596	-0.704143
C 0	1.602919	0.189897	0.491421
C 0	2.995230	0.018464	0.509670
C 0	3.728403	0.128943	-0.665935
C 0	3.066104	0.426617	-1.865696
C 0	1.693984	0.592036	-1.884379
O 0	5.080451	-0.033736	-0.759621
C 0	5.780823	-0.331991	0.427734
H 0	-4.110945	-0.774176	-2.910791
H 0	-1.977603	-0.773645	-2.150185
H 0	-2.125831	-2.406967	0.328769
H 0	-5.628002	-2.766591	-2.161990
H 0	-2.364011	-4.544866	-0.758353
H 0	-3.588086	-2.690926	-4.090485
H 0	-4.370399	-4.235319	-3.628394
H 0	-6.110164	-0.527641	-1.366826
H 0	-1.686164	2.703558	-0.730401
H 0	-3.029919	4.544866	-1.699430
H 0	-5.371929	4.150306	-2.405280
H 0	-6.393185	1.895349	-2.127369
H 0	-6.830267	-1.426676	0.915324
H 0	-6.227499	-1.877560	3.289300
H 0	-3.927565	-1.424779	4.090485
H 0	-2.216563	-0.541427	2.550028
H 0	2.347785	-3.187701	0.056899
H 0	2.967831	-2.874451	-2.317850
H 0	1.185901	-2.532547	-2.729644
H 0	-0.846383	-0.620627	0.760384
H 0	-1.212689	1.102085	1.003972
H 0	1.056616	0.096452	1.423773
H 0	3.476901	-0.202523	1.455564
H 0	3.654240	0.514891	-2.774366
H 0	1.167623	0.809796	-2.808908
H 0	5.437752	-1.279490	0.862902
H 0	5.667509	0.469949	1.168049
H 0	6.830267	-0.420427	0.146339

Number of imaginary frequencies: 0

TS-2 [6b (*gauche_s-trans*)] ⇋ [6b (*anti_s-trans*)]

M06-2X/6-31+G(d) Geometry

C 0	-4.675567	-0.642497	-2.016458
C 0	-3.169184	-0.856077	-1.653709
C 0	-2.869097	-2.207407	-0.950202
C 0	-5.543432	-1.893600	-1.860502
O 0	-5.198222	-2.609226	-0.676233
C 0	-4.000509	-3.230787	-1.050289
C 0	-5.260724	-2.949535	-2.934037
O 0	-4.166922	-3.676098	-2.383147
C 0	-2.639694	0.361451	-0.759307
C 0	-3.498552	0.293620	0.520954
C 0	-4.878084	0.350212	0.264855
C 0	-5.219367	0.551269	-1.186601
C 0	-4.448471	1.749535	-1.670178
C 0	-3.057901	1.664891	-1.476085
C 0	-2.274514	2.736479	-1.914284
C 0	-2.869364	3.841966	-2.529652
C 0	-4.244217	3.904021	-2.723276
C 0	-5.038525	2.844098	-2.288609
C 0	-5.807661	0.251782	1.290624
C 0	-5.371192	0.098411	2.606398
C 0	-4.007517	0.048900	2.874737
C 0	-3.072591	0.140469	1.839042
O 0	-1.721047	-2.821822	-1.560603
C 0	-0.732490	-3.272356	-0.765167
O 0	-0.697099	-3.103278	0.434547
C 0	0.333277	-3.974898	-1.521305
C 0	0.251877	-4.245660	-2.823657
C 0	-1.135353	0.100722	-0.482307
O 0	-0.258211	1.075911	-1.006069
C 0	1.077551	0.793021	-0.910300
C 0	1.610660	-0.316338	-0.262192
C 0	3.000264	-0.508711	-0.230166
C 0	3.850083	0.404781	-0.842337
C 0	3.306458	1.523168	-1.490636
C 0	1.938693	1.713989	-1.524336
O 0	5.210774	0.306331	-0.868946
C 0	5.793071	-0.800297	-0.217671
H 0	-4.737432	-0.338510	-3.068161
H 0	-2.582630	-0.848403	-2.578499
H 0	-2.664202	-2.054868	0.113523
H 0	-6.602217	-1.628771	-1.791111
H 0	-3.812984	-4.090831	-0.402134
H 0	-4.966739	-2.520483	-3.896466
H 0	-6.112354	-3.624429	-3.073051
H 0	-6.297051	0.673839	-1.343511
H 0	-1.205447	2.730651	-1.778105
H 0	-2.238156	4.664343	-2.854481
H 0	-4.696517	4.768906	-3.199872
H 0	-6.118023	2.869795	-2.420668
H 0	-6.870329	0.295084	1.062301
H 0	-6.091821	0.020680	3.415161
H 0	-3.657132	-0.064286	3.896466
H 0	-2.020709	0.092494	2.098851
H 0	1.186206	-4.264089	-0.915147
H 0	1.053179	-4.768906	-3.336107
H 0	-0.619309	-3.955404	-3.402772
H 0	-0.855746	-0.850949	-0.935498
H 0	-0.947357	0.006463	0.590290
H 0	0.978867	-1.047075	0.231615
H 0	3.387863	-1.381652	0.283183
H 0	3.983470	2.228800	-1.962067
H 0	1.508876	2.575633	-2.026502
H 0	5.460133	-1.745051	-0.666230
H 0	5.553042	-0.801888	0.853118
H 0	6.870329	-0.696133	-0.348409

Number of imaginary frequencies: 1 (-155.3)

6b (*anti_s-trans*)

M06-2X/6-31+G(d) Geometry

O 0	1.767978	3.800596	0.411308
O 0	2.250662	2.619382	-1.446804
C 0	1.572521	3.555126	-0.757365
C 0	0.538277	4.240039	-1.574909
C 0	0.275508	3.931237	-2.844591
C 0	3.188125	1.819506	-0.708399
C 0	4.501492	2.566237	-0.498271
O 0	5.080277	2.949454	-1.731998
C 0	6.124624	2.022391	-2.013576
C 0	5.862069	0.901770	-1.000756
C 0	4.802774	-0.084254	-1.505413
C 0	3.351871	0.507767	-1.497333
O 0	5.388982	1.661960	0.109147
C 0	2.348579	-0.586881	-0.952356
C 0	4.796350	-1.457083	-0.763045
C 0	0.923013	-0.070885	-1.064561
C 0	2.611301	-1.849959	-1.764255
C 0	3.920167	-2.333990	-1.630217
C 0	4.331337	-3.465736	-2.322430
C 0	3.426719	-4.126720	-3.156905
C 0	2.128463	-3.643395	-3.299543
C 0	1.715067	-2.503666	-2.604144
C 0	2.790912	-0.862901	0.480194
C 0	4.120753	-1.293116	0.576062
C 0	4.702584	-1.522066	1.816321
C 0	3.949881	-1.322630	2.974934
C 0	2.626764	-0.897019	2.882755
C 0	2.041908	-0.663573	1.635469
O 0	0.037565	-1.077152	-0.621720
C 0	-1.297975	-0.783486	-0.620220
C 0	-2.143690	-1.811059	-0.178396
C 0	-3.511273	-1.619570	-0.138602
C 0	-4.068128	-0.395769	-0.538266
C 0	-3.232167	0.624935	-0.976001
C 0	-1.843116	0.431736	-1.017629
O 0	-5.427211	-0.308463	-0.460000
C 0	-6.022234	0.910027	-0.845643
H 0	0.001060	5.017480	-1.040453
H 0	0.829671	3.153007	-3.360949
H 0	-0.496839	4.455923	-3.398701
H 0	2.781565	1.635084	0.291830
H 0	4.373344	3.441150	0.141897
H 0	7.095984	2.499136	-1.840878
H 0	6.049449	1.699860	-3.055600
H 0	6.771540	0.383020	-0.684779
H 0	5.064414	-0.313917	-2.544807
H 0	3.071773	0.732214	-2.532657
H 0	5.813442	-1.854528	-0.672183
H 0	0.792156	0.829970	-0.447972
H 0	0.701469	0.202880	-2.108541
H 0	5.349722	-3.832167	-2.213362
H 0	3.736984	-5.017480	-3.695635
H 0	1.426581	-4.158810	-3.948827
H 0	0.694005	-2.148800	-2.705363

H 0	5.737227	-1.851719	1.880161
H 0	4.397377	-1.498594	3.948827
H 0	2.042538	-0.744271	3.785578
H 0	1.006096	-0.340807	1.575448
H 0	-1.698304	-2.751930	0.130623
H 0	-4.178031	-2.405428	0.202495
H 0	-3.631189	1.582739	-1.290214
H 0	-1.215948	1.247327	-1.361381
H 0	-5.810635	1.139277	-1.897808
H 0	-5.674631	1.738212	-0.215014
H 0	-7.095984	0.777971	-0.711867

Number of imaginary frequencies: 0

TS-3 [6b (*anti_s-trans*)] ⇋ [6b (*anti_s-cis*)]

M06-2X/6-31+G(d) Geometry

C 0	-2.245283	-2.224165	-0.087474
C 0	-1.379396	-1.234889	-0.575995
C 0	-1.901402	-0.028510	-1.027070
C 0	-3.286078	0.195978	-0.993256
C 0	-4.141325	-0.786930	-0.508812
C 0	-3.608277	-2.002698	-0.054906
O 0	-0.051237	-1.556160	-0.568256
C 0	0.862667	-0.575659	-1.012506
C 0	2.269224	-1.141360	-0.905679
C 0	3.315206	-0.075097	-1.427059
C 0	4.739717	-0.728270	-1.447790
C 0	4.677138	-2.116147	-0.736109
C 0	4.008777	-1.953526	0.606592
C 0	2.698897	-1.464260	0.521070
C 0	3.205261	1.226186	-0.609781
C 0	4.551136	1.901416	-0.352123
O 0	5.390619	0.934419	0.223468
C 0	5.836301	0.197049	-0.912552
C 0	3.767383	-2.939080	-1.620088
C 0	2.480460	-2.397862	-1.743920
O 0	5.162682	2.313871	-1.559783
C 0	6.150273	1.339841	-1.886150
C 0	4.579828	-2.234501	1.841023
C 0	3.836866	-2.026569	3.004352
C 0	2.534191	-1.539932	2.922818
C 0	1.960081	-1.255054	1.681296
C 0	1.558217	-2.997169	-2.596353
C 0	1.923780	-4.139638	-3.313751
C 0	3.200314	-4.680287	-3.180222
C 0	4.131195	-4.074096	-2.333147
O 0	2.330874	2.096157	-1.353164
C 0	1.761044	3.108941	-0.677510
C 0	0.846336	3.906146	-1.554645
C 0	1.255567	4.991223	-2.203125
O 0	1.952147	3.327468	0.494273
O 0	-5.497876	-0.670218	-0.433170
C 0	-6.071748	0.541211	-0.870038
H 0	-1.818972	-3.159266	0.262789
H 0	-1.255667	0.752366	-1.413141
H 0	-3.667457	1.145416	-1.351503
H 0	-4.290746	-2.758036	0.322007
H 0	0.651320	-0.295523	-2.056227
H 0	0.760420	0.328494	-0.394578
H 0	3.043947	0.183189	-2.456617
H 0	4.994979	-0.943381	-2.491879
H 0	5.677805	-2.555028	-0.653995
H 0	2.765079	1.043068	0.376437
H 0	4.455326	2.751660	0.325051
H 0	6.723103	-0.368850	-0.613952
H 0	6.048184	1.059241	-2.938125
H 0	7.147393	1.757395	-1.707912
H 0	5.599174	-2.609924	1.897421
H 0	4.276261	-2.242854	3.973838
H 0	1.957807	-1.379663	3.829355
H 0	0.939415	-0.886161	1.628071
H 0	0.553069	-2.598064	-2.692769
H 0	1.201448	-4.611100	-3.973838
H 0	3.473542	-5.572652	-3.735897
H 0	5.133077	-4.485065	-2.230280
H 0	-0.182947	3.557796	-1.609965
H 0	0.572370	5.572652	-2.814036
H 0	2.285396	5.333051	-2.143756
H 0	-5.858356	0.721589	-1.931335
H 0	-5.708360	1.389271	-0.275683
H 0	-7.147393	0.434101	-0.729600

Number of imaginary frequencies: 1 (-139.0)

6b (*anti_s-cis*)

M06-2X/6-31+G(d) Geometry

O 0	1.577402	3.541218	0.045106
O 0	2.234713	2.367742	-1.765783
C 0	1.490578	3.296778	-1.136137
C 0	0.549707	3.950316	-2.084150
C 0	-0.316588	4.859861	-1.641395
C 0	3.164942	1.622210	-0.963185
C 0	4.446034	2.424555	-0.749441
O 0	5.031021	2.798246	-1.982242
C 0	6.092639	1.884122	-2.238808
C 0	5.872533	0.797610	-1.178238
C 0	4.861016	-0.255909	-1.639193
C 0	3.389457	0.279559	-1.683586
O 0	5.361483	1.579326	-0.101033
C 0	2.419481	-0.823668	-1.093755
C 0	4.896028	-1.580590	-0.815899
C 0	0.979286	-0.367570	-1.260342
C 0	2.746373	-2.120147	-1.825264
C 0	4.070469	-2.542087	-1.641679
C 0	4.539341	-3.695176	-2.257326
C 0	3.676964	-4.441627	-3.064144
C 0	2.363507	-4.020939	-3.256152
C 0	1.892157	-2.859169	-2.638149
C 0	2.846298	-0.998891	0.359389
C 0	4.191249	-1.365173	0.500754
C 0	4.760289	-1.492515	1.761256
C 0	3.979776	-1.256165	2.894131
C 0	2.641555	-0.894580	2.756850
C 0	2.069437	-0.762326	1.488943
O 0	0.117052	-1.374543	-0.774154
C 0	-1.224670	-1.109866	-0.787445
C 0	-2.049288	-2.124554	-0.280801
C 0	-3.420475	-1.957637	-0.248946
C 0	-4.002444	-0.772015	-0.721332
C 0	-3.187822	0.235686	-1.224087
C 0	-1.795584	0.066882	-1.258229
O 0	-5.362846	-0.706186	-0.643642
C 0	-5.982791	0.473689	-1.102862
H 0	0.610629	3.648629	-3.125345
H 0	-0.334756	5.130216	-0.588873
H 0	-1.015567	5.350580	-2.311097
H 0	2.730837	1.471631	0.030410

H 0 4.268702 3.312348 -0.138956
H 0 7.053548 2.391786 -2.100192
H 0 6.013050 1.514129 -3.264838
H 0 6.803100 0.333824 -0.838982
H 0 5.147963 -0.535294 -2.659705
H 0 3.116325 0.440144 -2.732520
H 0 5.926216 -1.930673 -0.685792
H 0 0.806886 0.562584 -0.699940
H 0 0.769672 -0.163176 -2.322192
H 0 5.569534 -4.011954 -2.109923
H 0 4.032367 -5.350580 -3.541116
H 0 1.694945 -4.603620 -3.883277
H 0 0.859765 -2.553162 -2.777246
H 0 5.806859 -1.771907 1.860668
H 0 4.417359 -1.354977 3.883277
H 0 2.035738 -0.711836 3.639625
H 0 1.022881 -0.486246 1.393632
H 0 -1.585629 -3.035896 0.084440
H 0 -4.071331 -2.733721 0.141570
H 0 -3.605504 1.164554 -1.595559
H 0 -1.186135 0.871356 -1.655038
H 0 -5.776827 0.641918 -2.167642
H 0 -5.651668 1.346319 -0.525520
H 0 -7.053548 0.328574 -0.958961
Number of imaginary frequencies: 0

TS-4 [6b (*anti_s-cis*)] ⇋ [6b (*gauche_s-cis*)]
M06-2X/6-31+G(d) Geometry
C 0 -4.695377 -0.791254 -1.959023
C 0 -3.180003 -0.994936 -1.631660
C 0 -2.837899 -2.387384 -1.036629
C 0 -5.530263 -2.072271 -1.885525
O 0 -5.152426 -2.863194 -0.760211
C 0 -3.946817 -3.429151 -1.193256
C 0 -5.235389 -3.040567 -3.036987
O 0 -4.124458 -3.785608 -2.549374
C 0 -2.666975 0.164580 -0.654221
C 0 -3.507263 -0.017780 0.626170
C 0 -4.890787 0.026302 0.390981
C 0 -5.255622 0.325340 -1.037736
C 0 -4.517810 1.573385 -1.441240
C 0 -3.123336 1.507000 -1.269256
C 0 -2.368729 2.624465 -1.638402
C 0 -2.995400 3.756894 -2.166757
C 0 -4.373693 3.801323 -2.340108
C 0 -5.139465 2.695479 -1.973396
C 0 -5.804129 -0.168645 1.417635
C 0 -5.347015 -0.410046 2.713121
C 0 -3.979246 -0.450225 2.960794
C 0 -3.060666 -0.260305 1.923937
O 0 -1.682858 -2.919579 -1.708941
C 0 -0.665720 -3.390561 -0.962824
O 0 -0.640345 -3.364027 0.247889
C 0 0.426488 -3.898657 -1.832343
C 0 1.565217 -4.324717 -1.289109
C 0 -1.153849 -0.078137 -0.411603
O 0 -0.303735 0.947603 -0.880621
C 0 1.038769 0.689997 -0.812919
C 0 1.602589 -0.458511 -0.266963
C 0 2.996651 -0.618935 -0.258512
C 0 3.820544 0.366012 -0.789311
C 0 3.246134 1.523622 -1.334362
C 0 1.874084 1.682421 -1.346533
O 0 5.183068 0.304238 -0.829586
C 0 5.797076 -0.845680 -0.293046
H 0 -4.778949 -0.412958 -2.984780
H 0 -2.607422 -0.903416 -2.560777
H 0 -2.618633 -2.311312 0.032447
H 0 -6.594244 -1.840447 -1.784626
H 0 -3.729104 -4.326659 -0.608609
H 0 -4.958040 -2.535218 -3.966853
H 0 -6.075056 -3.719755 -3.220340
H 0 -6.337709 0.433334 -1.173588
H 0 -1.297978 2.632486 -1.515756
H 0 -2.386813 4.614085 -2.440641
H 0 -4.849943 4.687303 -2.750023
H 0 -6.220775 2.705877 -2.091939
H 0 -6.870462 -0.131647 1.205990
H 0 -6.055477 -0.562655 3.521914
H 0 -3.612797 -0.632334 3.966853
H 0 -2.004791 -0.305020 2.167347
H 0 0.248607 -3.884546 -2.902832
H 0 2.384668 -4.687303 -1.901211
H 0 1.693509 -4.316154 -0.209655
H 0 -0.859489 -0.991191 -0.929175
H 0 -0.949072 -0.236257 0.650368
H 0 0.992604 -1.246830 0.161823
H 0 3.408046 -1.525065 0.172016
H 0 3.903082 2.283898 -1.745218
H 0 1.421303 2.573787 -1.770443
H 0 5.484668 -1.749819 -0.831440
H 0 5.565293 -0.959070 0.773636
H 0 6.870462 -0.701925 -0.418004
Number of imaginary frequencies: 1 (-164.7)

6c (*gauche_s-cis*)
M06-2X/6-31+G(d) Geometry
C 0 -3.092479 -1.267394 -1.892297
C 0 -1.635699 -1.085254 -1.342312
C 0 -0.964488 -2.370087 -0.815105
C 0 -3.496436 -2.735153 -2.067907
O 0 -3.007011 -3.542435 -0.998077
C 0 -1.639754 -3.648163 -1.303021
C 0 -2.790825 -3.389706 -3.260296
O 0 -1.540546 -3.787516 -2.707027
C 0 -1.652831 0.018127 -0.210982
C 0 -2.558334 -0.535410 0.885260
C 0 -3.858030 -0.816510 0.441948
C 0 -4.085723 -0.454283 -1.005189
C 0 -3.674349 0.989347 -1.186907
C 0 -2.354406 1.240437 -0.789106
C 0 -1.816938 2.515049 -0.936656
C 0 -2.607357 3.537706 -1.468998
C 0 -3.919494 3.285562 -1.860982
C 0 -4.456532 2.002404 -1.725504
C 0 -4.793582 -1.375320 1.301767
C 0 -4.434813 -1.652973 2.622893
C 0 -3.145992 -1.374380 3.068183
C 0 -2.200900 -0.820642 2.199604
O 0 0.398514 -2.379504 -1.258244
C 0 1.375942 -2.601209 -0.358007
O 0 1.183420 -2.756197 0.828753
C 0 2.702712 -2.609266 -1.023537
C 0 3.813794 -2.718806 -0.297622
C 0 -0.253249 0.302078 0.307803

O 0	0.577113	0.643775	-0.788465
C 0	1.925809	0.636002	-0.593248
C 0	2.536023	0.508077	0.655726
C 0	3.929839	0.497577	0.735709
C 0	4.714335	0.621065	-0.406338
C 0	4.091909	0.759881	-1.649945
C 0	2.707426	0.765214	-1.747526
H 0	-3.138499	-0.807621	-2.886543
H 0	-1.001597	-0.713836	-2.151682
H 0	-0.985335	-2.409044	0.277670
H 0	-4.583917	-2.848574	-2.097699
H 0	-1.222894	-4.534381	-0.817071
H 0	-2.616000	-2.704869	-4.094661
H 0	-3.334193	-4.273976	-3.611949
H 0	-5.120480	-0.629404	-1.320866
H 0	-0.787097	2.715836	-0.655757
H 0	-2.190362	4.534381	-1.580617
H 0	-4.527164	4.085904	-2.273446
H 0	-5.480222	1.797607	-2.031413
H 0	-5.797463	-1.595104	0.945300
H 0	-5.161948	-2.087692	3.302450
H 0	-2.867118	-1.593738	4.094661
H 0	-1.195468	-0.626517	2.564220
H 0	2.709394	-2.499793	-2.103238
H 0	4.795697	-2.709877	-0.759663
H 0	3.755545	-2.810596	0.783819
H 0	0.148507	-0.584322	0.813185
H 0	-0.280572	1.127215	1.036511
H 0	1.948061	0.405342	1.560850
H 0	4.399021	0.390392	1.709873
H 0	5.797463	0.613867	-0.331759
H 0	4.690344	0.858036	-2.551512
H 0	2.203806	0.862695	-2.704671

Number of imaginary frequencies: 0

TS-1 [6c (*gauche_s-cis*)] ⇋ [6c (*gauche_s-trans*)]

M06-2X/6-31+G(d) Geometry

C 0	2.653340	1.071209	-1.696286
C 0	1.866744	0.899099	-0.551190
C 0	2.471329	0.772415	0.701679
C 0	3.865157	0.807744	0.794567
C 0	4.654093	0.973484	-0.338618
C 0	4.036578	1.108922	-1.586325
O 0	0.522158	0.862157	-0.759203
C 0	-0.309849	0.477137	0.320586
C 0	-1.672535	0.096668	-0.232038
C 0	-1.543298	-1.034315	-1.326956
C 0	-2.960912	-1.316567	-1.930976
C 0	-4.038263	-0.558001	-1.095202
C 0	-3.838051	-0.869157	0.367613
C 0	-2.578883	-0.485470	0.849062
C 0	-0.831315	-2.262869	-0.726923
C 0	-1.397689	-3.591551	-1.220490
O 0	-2.784045	-3.568493	-0.994428
C 0	-3.260248	-2.808615	-2.103905
C 0	-3.719574	0.906262	-1.297626
C 0	-2.432197	1.255458	-0.866057
O 0	-1.211623	-3.753088	-2.612582
C 0	-2.446971	-3.432023	-3.244214
C 0	-4.764214	-1.469646	1.209227
C 0	-4.436977	-1.686466	2.549561
C 0	-3.188378	-1.305870	3.032827
C 0	-2.252296	-0.709276	2.183052
C 0	-1.978626	2.560258	-1.027627
C 0	-2.819401	3.514561	-1.608204
C 0	-4.098796	3.164915	-2.032609
C 0	-4.551280	1.851350	-1.882799
O 0	0.558704	-2.200641	-1.087957
C 0	1.478614	-2.450259	-0.138256
C 0	2.863298	-2.460679	-0.706784
C 0	3.431997	-3.583606	-1.133350
O 0	1.216281	-2.633867	1.028251
H 0	2.155795	1.159833	-2.657040
H 0	1.880878	0.637798	1.600994
H 0	4.328632	0.697696	1.770651
H 0	5.736035	0.997866	-0.255471
H 0	4.638731	1.236440	-2.481503
H 0	0.131989	-0.379617	0.844398
H 0	-0.405570	1.304269	1.040895
H 0	-0.893756	-0.652944	-2.119600
H 0	-2.996041	-0.872112	-2.932690
H 0	-5.047231	-0.810076	-1.440652
H 0	-0.915785	-2.277722	0.363102
H 0	-0.954001	-4.439228	-0.691079
H 0	-4.334941	-2.991017	-2.192964
H 0	-2.261571	-2.744738	-4.074411
H 0	-2.915355	-4.349194	-3.618432
H 0	-5.736035	-1.769783	0.823846
H 0	-5.157189	-2.153765	3.214775
H 0	-2.933829	-1.478271	4.074411
H 0	-1.277027	-0.434338	2.576819
H 0	-0.975128	2.837789	-0.718765
H 0	-2.467636	4.534910	-1.730113
H 0	-4.746648	3.912675	-2.480982
H 0	-5.548446	1.569839	-2.213929
H 0	3.383545	-1.504732	-0.725210
H 0	4.447366	-3.585256	-1.516907
H 0	2.906558	-4.534910	-1.113332

Number of imaginary frequencies: 1 (-138.2)

6c (*gauche_s-trans*)

M06-2X/6-31+G(d) Geometry

C 0	-2.951863	-1.281528	-1.914961
C 0	-1.536177	-1.088947	-1.271268
C 0	-0.905969	-2.365076	-0.678989
C 0	-3.330272	-2.752017	-2.121919
O 0	-2.911787	-3.559675	-1.022944
C 0	-1.523874	-3.647381	-1.224860
C 0	-2.532917	-3.396226	-3.260801
O 0	-1.317618	-3.770889	-2.619920
C 0	-1.629758	0.027431	-0.156919
C 0	-2.594731	-0.523190	0.888853
C 0	-3.860889	-0.825515	0.368483
C 0	-4.005197	-0.475986	-1.092649
C 0	-3.598679	0.971386	-1.257969
C 0	-2.307391	1.239252	-0.784048
C 0	-1.778062	2.520330	-0.902218
C 0	-2.547144	3.531704	-1.484876
C 0	-3.829976	3.262348	-1.954572
C 0	-4.359033	1.973336	-1.846368
C 0	-4.839357	-1.390044	1.175119
C 0	-4.558217	-1.650561	2.518431
C 0	-3.303237	-1.349493	3.039710
C 0	-2.314115	-0.790788	2.225019
O 0	0.494914	-2.357564	-0.985973

C 0 1.365394 -2.684875 -0.009945
O 0 1.038333 -2.915608 1.135082
C 0 2.772409 -2.716508 -0.476663
C 0 3.137095 -2.489359 -1.737839
C 0 -0.259228 0.322339 0.429074
O 0 0.604088 0.697204 -0.628923
C 0 1.948994 0.644418 -0.421201
C 0 2.544937 0.377652 0.812081
C 0 3.938709 0.318649 0.897652
C 0 4.735069 0.531805 -0.221320
C 0 4.125849 0.814855 -1.447475
C 0 2.743007 0.871660 -1.550714
H 0 -2.935892 -0.819571 -2.909192
H 0 -0.847752 -0.728453 -2.040437
H 0 -1.031044 -2.402711 0.406579
H 0 -4.411706 -2.874695 -2.230059
H 0 -1.134606 -4.533182 -0.715785
H 0 -2.311199 -2.710496 -4.083182
H 0 -3.035731 -4.290183 -3.646926
H 0 -5.017634 -0.663585 -1.467735
H 0 -0.769969 2.734248 -0.559457
H 0 -2.136376 4.533182 -1.574635
H 0 -4.420724 4.053950 -2.406630
H 0 -5.360240 1.755792 -2.212003
H 0 -5.816812 -1.626547 0.760430
H 0 -5.319567 -2.090086 3.156229
H 0 -3.084580 -1.556340 4.083182
H 0 -1.333803 -0.581702 2.645716
H 0 3.489803 -2.927943 0.310151
H 0 4.183134 -2.510676 -2.028071
H 0 2.400653 -2.271281 -2.505337
H 0 0.127858 -0.569889 0.935518
H 0 -0.322944 1.133291 1.171153
H 0 1.949035 0.205748 1.701691
H 0 4.396448 0.104674 1.859544
H 0 5.816812 0.485202 -0.142807
H 0 4.734020 0.990181 -2.330845
H 0 2.249807 1.078090 -2.495944
Number of imaginary frequencies: 0

TS-2 [6c (*gauche_s-trans*)] ⇋ [6c (*anti_s-trans*)]

M06-2X/6-31+G(d) Geometry
C 0 -3.625538 -0.606525 -2.033287
C 0 -2.133824 -0.822296 -1.615425
C 0 -1.863153 -2.169752 -0.892956
C 0 -4.503407 -1.853181 -1.897973
O 0 -4.201752 -2.561101 -0.697432
C 0 -2.994232 -3.189755 -1.025639
C 0 -4.189157 -2.918050 -2.953169
O 0 -3.115977 -3.643085 -2.360772
C 0 -1.632912 0.399965 -0.710661
C 0 -2.538468 0.345156 0.538007
C 0 -3.907495 0.404184 0.231262
C 0 -4.194509 0.595346 -1.233186
C 0 -3.402082 1.787419 -1.696835
C 0 -2.019757 1.699649 -1.451956
C 0 -1.217394 2.765486 -1.869579
C 0 -1.785689 3.868481 -2.513756
C 0 -3.152501 3.933917 -2.756587
C 0 -3.965540 2.879505 -2.343606
C 0 -4.874245 0.318059 1.223224
C 0 -4.486546 0.175836 2.555323
C 0 -3.133809 0.125513 2.873844
C 0 -2.161350 0.204159 1.872191
O 0 -0.697434 -2.792032 -1.459239
C 0 0.259694 -3.245120 -0.626749
O 0 0.257068 -3.062793 0.571391
C 0 1.340941 -3.967515 -1.340832
C 0 1.291661 -4.259231 -2.640334
C 0 -0.141274 0.135583 -0.375153
O 0 0.758932 1.103459 -0.877780
C 0 2.088016 0.835489 -0.722995
C 0 2.595802 -0.274513 -0.043174
C 0 3.981154 -0.441804 0.044784
C 0 4.854221 0.474143 -0.529430
C 0 4.332873 1.581979 -1.203875
C 0 2.961286 1.764305 -1.302135
H 0 -3.648649 -0.310867 -3.088871
H 0 -1.515189 -0.822730 -2.519121
H 0 -1.695116 -2.010753 0.176254
H 0 -5.562769 -1.583497 -1.866854
H 0 -2.832892 -4.046088 -0.365617
H 0 -3.862427 -2.497942 -3.908852
H 0 -5.037833 -3.591680 -3.114585
H 0 -5.265237 0.720480 -1.430117
H 0 -0.153981 2.757637 -1.695222
H 0 -1.140353 4.686271 -2.821938
H 0 -3.584576 4.797127 -3.254553
H 0 -5.039452 2.907745 -2.514739
H 0 -5.927739 0.362510 0.955946
H 0 -5.236274 0.107077 3.338059
H 0 -2.821626 0.021532 3.908852
H 0 -1.120040 0.155884 2.171541
H 0 2.174668 -4.253479 -0.707098
H 0 2.101456 -4.797127 -3.123393
H 0 0.438036 -3.971372 -3.246271
H 0 0.151031 -0.821744 -0.806785
H 0 0.007267 0.054491 0.704248
H 0 1.943110 -1.005902 0.421394
H 0 4.370115 -1.306188 0.576035
H 0 5.927739 0.332488 -0.453597
H 0 5.001764 2.308394 -1.656628
H 0 2.539950 2.618022 -1.824661
Number of imaginary frequencies: 1 (-157.3)

6c (*anti_s-trans*)

M06-2X/6-31+G(d) Geometry
O 0 0.689988 3.538467 0.032849
O 0 1.305955 2.333169 -1.771372
C 0 0.597927 3.294673 -1.148117
C 0 -0.310692 3.982824 -2.102709
C 0 -1.142343 4.927518 -1.667065
C 0 2.201160 1.552606 -0.963022
C 0 3.515064 2.298357 -0.745607
O 0 4.124345 2.640556 -1.976002
C 0 5.152615 1.684566 -2.217149
C 0 4.872464 0.609213 -1.159968
C 0 3.818487 -0.397157 -1.632855
C 0 2.370895 0.199799 -1.678593
O 0 4.386311 1.414327 -0.088332
C 0 1.354515 -0.859790 -1.086813
C 0 3.795111 -1.727322 -0.818072
C 0 -0.064228 -0.338993 -1.248683
C 0 1.620439 -2.167679 -1.822414
C 0 2.925787 -2.647613 -1.645890

C 0 3.340688 -3.817772 -2.268078
C 0 2.443004 -4.522726 -3.073587
C 0 1.147943 -4.044975 -3.257489
C 0 0.731027 -2.865988 -2.633282
C 0 1.776942 -1.056770 0.364915
C 0 3.103844 -1.486257 0.501466
C 0 3.670206 -1.642960 1.759864
C 0 2.905104 -1.372553 2.895788
C 0 1.585107 -0.947688 2.763375
C 0 1.015809 -0.786089 1.497518
O 0 -0.969140 -1.308655 -0.758519
C 0 -2.297566 -1.001845 -0.779612
C 0 -3.153122 -1.988671 -0.275885
C 0 -4.523382 -1.770803 -0.256748
C 0 -5.058789 -0.571851 -0.736738
C 0 -4.202126 0.402878 -1.234320
C 0 -2.819479 0.200618 -1.261411
H 0 -0.256496 3.675893 -3.142727
H 0 -1.155673 5.201259 -0.615373
H 0 -1.817684 5.443979 -2.341638
H 0 1.758964 1.423822 0.030092
H 0 3.374596 3.195526 -0.139041
H 0 6.130936 2.153259 -2.063995
H 0 5.073389 1.318536 -3.244543
H 0 5.777048 0.103095 -0.810942
H 0 4.096793 -0.681818 -2.654240
H 0 2.105500 0.368920 -2.728094
H 0 4.808848 -2.123636 -0.691821
H 0 -0.193849 0.596593 -0.686980
H 0 -0.269173 -0.126528 -2.309292
H 0 4.356491 -4.180406 -2.126889
H 0 2.756456 -5.443979 -3.556357
H 0 0.451439 -4.595369 -3.883374
H 0 -0.287457 -2.514487 -2.767050
H 0 4.702708 -1.971673 1.855434
H 0 3.340732 -1.493081 3.883374
H 0 0.991529 -0.737447 3.648271
H 0 -0.016496 -0.459624 1.406484
H 0 -2.716638 -2.911503 0.093776
H 0 -5.180698 -2.541115 0.136319
H 0 -6.130936 -0.403744 -0.720102
H 0 -4.602721 1.340399 -1.609567
H 0 -2.173832 0.978787 -1.653135
Number of imaginary frequencies: 0

TS-3 [6c (*anti_s-trans*)] ⇋ [6c (*anti_s-cis*)]
M06-2X/6-31+G(d) Geometry
C 0 -3.655164 -1.425262 -0.088265
C 0 -2.615235 -0.588273 -0.509554
C 0 -2.895375 0.694915 -0.982461
C 0 -4.222933 1.129532 -1.032186
C 0 -5.261309 0.304630 -0.616742
C 0 -4.967302 -0.977196 -0.142813
O 0 -1.362639 -1.118160 -0.423454
C 0 -0.283320 -0.352326 -0.922473
C 0 0.976149 -1.202037 -0.884956
C 0 2.189403 -0.372697 -1.469744
C 0 3.440482 -1.307533 -1.577442
C 0 3.134564 -2.657175 -0.855905
C 0 2.596602 -2.367783 0.524190
C 0 1.411786 -1.618964 0.515008
C 0 2.401382 0.915117 -0.648994
C 0 3.871879 1.282052 -0.448225
O 0 4.522462 0.146247 0.058364
C 0 4.735356 -0.635549 -1.113815
C 0 2.021958 -3.266266 -1.679359
C 0 0.871059 -2.467419 -1.728708
O 0 4.493009 1.597657 -1.679654
C 0 5.213996 0.441522 -2.096278
C 0 3.170945 -2.765176 1.724378
C 0 2.556744 -2.416464 2.928518
C 0 1.377382 -1.675024 2.922289
C 0 0.801118 -1.270881 1.715328
C 0 -0.203741 -2.853183 -2.524248
C 0 -0.127387 -4.040376 -3.257438
C 0 1.013624 -4.836732 -3.197527
C 0 2.098700 -4.445410 -2.409162
O 0 1.711616 1.961285 -1.359264
C 0 1.411618 3.073689 -0.666610
C 0 0.679604 4.067015 -1.514519
C 0 1.309851 5.032472 -2.174900
O 0 1.684223 3.233714 0.498465
H 0 -3.404110 -2.418132 0.272335
H 0 -2.101576 1.356836 -1.310137
H 0 -4.435996 2.128673 -1.401900
H 0 -6.288752 0.651763 -0.659858
H 0 -5.768759 -1.633345 0.184475
H 0 -0.483159 -0.037756 -1.957897
H 0 -0.163866 0.554160 -0.312087
H 0 1.916904 -0.050783 -2.480916
H 0 3.582114 -1.561788 -2.634480
H 0 4.025263 -3.294896 -0.831997
H 0 1.968466 0.827272 0.353297
H 0 3.990220 2.112163 0.249933
H 0 5.506006 -1.376845 -0.883677
H 0 4.972702 0.217759 -3.139198
H 0 6.288752 0.628253 -1.995319
H 0 4.093163 -3.341776 1.722504
H 0 2.999098 -2.725139 3.871291
H 0 0.899672 -1.407632 3.860327
H 0 -0.123067 -0.699807 1.719407
H 0 -1.107629 -2.252763 -2.565414
H 0 -0.969944 -4.345163 -3.871291
H 0 1.061300 -5.761710 -3.764952
H 0 2.998035 -5.055821 -2.366569
H 0 -0.403730 3.968950 -1.536497
H 0 0.760896 5.761710 -2.762186
H 0 2.392251 5.125213 -2.148556
Number of imaginary frequencies: 1 (-129.3)

6c (*anti_s-cis*)
M06-2X/6-31+G(d) Geometry
O 0 0.905083 3.809840 0.407553
O 0 1.347322 2.614188 -1.451195
C 0 0.703966 3.574899 -0.762258
C 0 -0.301516 4.298674 -1.582091
C 0 -0.573602 3.997701 -2.851767
C 0 2.234332 1.763142 -0.708338
C 0 3.588549 2.430743 -0.491039
O 0 4.197243 2.780286 -1.720373
C 0 5.185327 1.791826 -1.997842
C 0 4.848994 0.687121 -0.989619
C 0 3.735854 -0.233102 -1.502676
C 0 2.323214 0.443246 -1.494906
O 0 4.415283 1.472270 0.119175

C 0 1.256387 -0.589719 -0.948371
C 0 3.646779 -1.605844 -0.765776
C 0 -0.133409 0.015703 -1.058582
C 0 1.440037 -1.865042 -1.763030
C 0 2.717850 -2.427173 -1.632210
C 0 3.057756 -3.582209 -2.324361
C 0 2.113039 -4.187241 -3.157033
C 0 0.846429 -3.625591 -3.297779
C 0 0.504377 -2.462776 -2.601608
C 0 1.683629 -0.894999 0.483207
C 0 2.984924 -1.405943 0.575107
C 0 3.553858 -1.673710 1.813379
C 0 2.816779 -1.432354 2.974106
C 0 1.522190 -0.925721 2.886138
C 0 0.950643 -0.652804 1.640654
O 0 -1.084274 -0.931790 -0.615522
C 0 -2.393129 -0.549754 -0.591399
C 0 -3.294955 -1.512910 -0.124088
C 0 -4.649377 -1.217913 -0.061788
C 0 -5.122565 0.035085 -0.462582
C 0 -4.219983 0.985316 -0.925065
C 0 -2.852282 0.705878 -0.994761
H 0 -0.811707 5.095148 -1.049302
H 0 -0.046817 3.199289 -3.365881
H 0 -1.325863 4.548459 -3.408174
H 0 1.814307 1.604892 0.290599
H 0 3.510327 3.310555 0.150273
H 0 6.182502 2.209018 -1.818139
H 0 5.097408 1.476931 -3.041189
H 0 5.723755 0.114418 -0.669131
H 0 3.986460 -0.473493 -2.542317
H 0 2.055217 0.681552 -2.530317
H 0 4.638354 -2.063718 -0.677580
H 0 -0.206271 0.920826 -0.439784
H 0 -0.337805 0.303920 -2.101666
H 0 4.052133 -4.009814 -2.216512
H 0 2.368088 -5.095148 -3.695905
H 0 0.113512 -4.096415 -3.946583
H 0 -0.493140 -2.046016 -2.702530
H 0 4.566683 -2.065601 1.875001
H 0 3.254623 -1.638036 3.946583
H 0 0.950466 -0.738779 3.790447
H 0 -0.063020 -0.265567 1.583314
H 0 -2.905729 -2.477981 0.185672
H 0 -5.342603 -1.969755 0.304439
H 0 -6.182502 0.263300 -0.412230
H 0 -4.572517 1.963679 -1.239316
H 0 -2.169779 1.466282 -1.358772
Number of imaginary frequencies: 0

TS-4 [6c (*anti_s-cis*)] ⇋ [6c (*gauche_s-cis*)]

M06-2X/6-31+G(d) Geometry
C 0 -3.642286 -0.846025 -1.963283
C 0 -2.132174 -1.025550 -1.600426
C 0 -1.783983 -2.412724 -0.996671
C 0 -4.456523 -2.141700 -1.919609
O 0 -4.095546 -2.934346 -0.790032
C 0 -2.867713 -3.473885 -1.193181
C 0 -4.113105 -3.095824 -3.068882
O 0 -3.000864 -3.822475 -2.556752
C 0 -1.662180 0.143283 -0.611790
C 0 -2.526326 -0.055758 0.650316
C 0 -3.905031 -0.038672 0.384697
C 0 -4.244198 0.255992 -1.051093
C 0 -3.522032 1.519029 -1.435552
C 0 -2.130354 1.478334 -1.235140
C 0 -1.389779 2.610900 -1.586648
C 0 -2.026804 3.732886 -2.124802
C 0 -3.401957 3.752079 -2.325434
C 0 -4.153785 2.630780 -1.977331
C 0 -4.836770 -0.253216 1.390694
C 0 -4.403567 -0.485430 2.695988
C 0 -3.041042 -0.497522 2.974011
C 0 -2.103569 -0.289379 1.957614
O 0 -0.598128 -2.924177 -1.629882
C 0 0.405650 -3.369809 -0.849744
O 0 0.396046 -3.327725 0.360693
C 0 1.529595 -3.869883 -1.682037
C 0 2.658085 -4.274117 -1.101734
C 0 -0.150618 -0.072215 -0.336431
O 0 0.689309 0.973644 -0.784289
C 0 2.033110 0.758362 -0.685271
C 0 2.611492 -0.384080 -0.126116
C 0 4.005054 -0.490521 -0.088036
C 0 4.817991 0.517662 -0.590907
C 0 4.226387 1.657069 -1.144237
C 0 2.845579 1.779301 -1.193293
H 0 -3.706879 -0.463198 -2.988736
H 0 -1.539469 -0.926043 -2.516123
H 0 -1.603937 -2.335175 0.079540
H 0 -5.526715 -1.928999 -1.846347
H 0 -2.649339 -4.371933 -0.609618
H 0 -3.821419 -2.579087 -3.987995
H 0 -4.934385 -3.789838 -3.278092
H 0 -5.324912 0.343652 -1.210173
H 0 -0.322035 2.639442 -1.442900
H 0 -1.428880 4.601829 -2.384743
H 0 -3.886622 4.630054 -2.742641
H 0 -5.232450 2.621058 -2.117903
H 0 -5.898653 -0.237733 1.155458
H 0 -5.126333 -0.652335 3.489133
H 0 -2.693474 -0.672475 3.987995
H 0 -1.052667 -0.314115 2.224399
H 0 1.382314 -3.869233 -2.757242
H 0 3.500447 -4.630054 -1.685943
H 0 2.755602 -4.252408 -0.019264
H 0 0.172763 -0.976489 -0.851235
H 0 0.033843 -0.228946 0.728981
H 0 2.008066 -1.188261 0.281753
H 0 4.448757 -1.381645 0.347604
H 0 5.898653 0.422772 -0.553944
H 0 4.847055 2.455190 -1.541526
H 0 2.371106 2.656120 -1.623772
Number of imaginary frequencies: 1 (-165.7)

6d (*gauche_s-cis*)

M06-2X/6-31+G(d) Geometry
C 0 -2.063220 -1.353940 -1.943741
C 0 -0.868027 -0.578639 -1.280765
C 0 0.266898 -1.461990 -0.712869
C 0 -1.857836 -2.873235 -2.001298
O 0 -1.159236 -3.345116 -0.849628
C 0 0.156875 -2.932138 -1.108621
C 0 -0.889356 -3.295872 -3.107957
O 0 0.382020 -3.114425 -2.493506
C 0 -1.439279 0.389521 -0.168687

C 0 -2.172644 -0.502497 0.829721
C 0 -3.210533 -1.248683 0.253485
C 0 -3.391837 -0.978569 -1.218114
C 0 -3.550178 0.510001 -1.406669
C 0 -2.490158 1.248031 -0.866161
C 0 -2.467403 2.631197 -1.016073
C 0 -3.516072 3.270350 -1.683699
C 0 -4.573597 2.532641 -2.210285
C 0 -4.588844 1.142228 -2.078242
C 0 -3.950714 -2.143431 1.013231
C 0 -3.660956 -2.297534 2.370644
C 0 -2.634055 -1.558350 2.949876
C 0 -1.884089 -0.661738 2.182062
O 0 1.517081 -0.974507 -1.215286
C 0 2.405927 -0.438615 -0.367404
O 0 2.293321 -0.431289 0.839455
C 0 3.554983 0.137833 -1.115657
C 0 4.560659 0.711969 -0.458322
C 0 -0.350420 1.215957 0.499398
O 0 0.462698 1.819052 -0.484266
C 0 1.398440 2.708194 0.084700
H 0 -2.168497 -1.000220 -2.975832
H 0 -0.404494 0.056200 -2.039912
H 0 0.282041 -1.429890 0.380816
H 0 -2.814934 -3.401335 -2.042732
H 0 0.853445 -3.554515 -0.541045
H 0 -0.948218 -2.675178 -4.005838
H 0 -1.022191 -4.350682 -3.374823
H 0 -4.232867 -1.538005 -1.643449
H 0 -1.634211 3.213437 -0.633179
H 0 -3.501110 4.350682 -1.795891
H 0 -5.384899 3.037332 -2.726882
H 0 -5.406878 0.557149 -2.493075
H 0 -4.750179 -2.718281 0.550607
H 0 -4.237712 -2.993006 2.973426
H 0 -2.409040 -1.676919 4.005838
H 0 -1.084467 -0.099760 2.656616
H 0 3.517257 0.068262 -2.198226
H 0 5.406878 1.146430 -0.980888
H 0 4.551494 0.753435 0.627960
H 0 0.278804 0.594476 1.149373
H 0 -0.823158 1.987331 1.132201
H 0 2.028284 3.077944 -0.726510
H 0 2.024216 2.194511 0.828626
H 0 0.888549 3.555591 0.567673
Number of imaginary frequencies: 0

TS-1 [6d (*gauche_s-cis*)] ⇋ [6d (*gauche_s-trans*)]
M06-2X/6-31+G(d) Geometry
C 0 1.353324 2.743208 0.564740
O 0 0.541873 1.834900 -0.148059
C 0 -0.319763 1.130859 0.721914
C 0 -1.331944 0.327321 -0.079300
C 0 -0.659396 -0.604007 -1.168656
C 0 -1.791424 -1.360401 -1.956694
C 0 -3.181402 -0.995632 -1.350509
C 0 -3.136818 -1.316962 0.122054
C 0 -2.152113 -0.597967 0.814302
C 0 0.424706 -1.508067 -0.535135
C 0 0.317985 -2.981482 -0.931540
O 0 -1.022619 -3.370987 -0.802354
C 0 -1.596199 -2.881095 -2.014064
C 0 -3.320153 0.498791 -1.501668
C 0 -2.317334 1.213833 -0.835664
O 0 0.676618 -3.180579 -2.284525
C 0 -0.530946 -3.309244 -3.026470
C 0 -3.937132 -2.238961 0.781203
C 0 -3.761942 -2.447690 2.151110
C 0 -2.789376 -1.733851 2.845001
C 0 -1.979056 -0.809458 2.178924
C 0 -2.294727 2.603074 -0.915496
C 0 -3.281969 3.268815 -1.646976
C 0 -4.278754 2.552674 -2.305736
C 0 -4.296257 1.157852 -2.238268
O 0 1.719004 -1.065004 -0.970458
C 0 2.572505 -0.523204 -0.094335
C 0 3.860065 -0.164009 -0.770066
C 0 4.124662 1.066361 -1.194724
O 0 2.357901 -0.379044 1.087056
H 0 1.926452 3.315237 -0.167893
H 0 2.036222 2.211750 1.242219
H 0 0.735225 3.440384 1.150543
H 0 0.280790 0.485847 1.375041
H 0 -0.867490 1.841069 1.365914
H 0 -0.134878 0.056663 -1.863850
H 0 -1.797312 -0.990484 -2.988389
H 0 -3.980138 -1.536851 -1.870230
H 0 0.379215 -1.466257 0.557281
H 0 0.944142 -3.612151 -0.295543
H 0 -2.551387 -3.395029 -2.155419
H 0 -0.484711 -2.667482 -3.910545
H 0 -0.663920 -4.353457 -3.331525
H 0 -4.693837 -2.794701 0.231551
H 0 -4.385037 -3.167200 2.674345
H 0 -2.654027 -1.895208 3.910545
H 0 -1.222285 -0.266522 2.738558
H 0 -1.507930 3.169574 -0.426828
H 0 -3.267054 4.353457 -1.704239
H 0 -5.042913 3.078047 -2.871326
H 0 -5.070237 0.590479 -2.750492
H 0 4.570468 -0.978655 -0.891784
H 0 5.070237 1.305747 -1.670849
H 0 3.398626 1.866807 -1.090768
Number of imaginary frequencies: 1 (-147.9)

6d (*gauche_s-trans*)
M06-2X/6-31+G(d) Geometry
C 0 -1.468406 -1.386189 -1.944003
C 0 -0.395384 -0.570943 -1.136547
C 0 0.731159 -1.409164 -0.488962
C 0 -1.137604 -2.877658 -2.073172
O 0 -0.529384 -3.377790 -0.883435
C 0 0.770501 -2.857420 -0.970362
C 0 -0.027185 -3.146881 -3.092466
O 0 1.156382 -2.937137 -2.330264
C 0 -1.133620 0.309932 -0.049656
C 0 -1.884330 -0.673852 0.843933
C 0 -2.806884 -1.468451 0.148802
C 0 -2.878542 -1.147848 -1.322382
C 0 -3.132762 0.333589 -1.465655
C 0 -2.180187 1.120745 -0.806667
C 0 -2.247907 2.507191 -0.900329
C 0 -3.281109 3.099710 -1.631817
C 0 -4.232584 2.312894 -2.276369
C 0 -4.155273 0.920238 -2.200235

C 0 -3.542208 -2.443943 0.807256
C 0 -3.363094 -2.631911 2.179479
C 0 -2.450893 -1.844715 2.876010
C 0 -1.705834 -0.866268 2.210853
O 0 1.991880 -0.819173 -0.826646
C 0 2.748762 -0.278672 0.137713
O 0 2.514292 -0.360470 1.324971
C 0 3.933461 0.437703 -0.401003
C 0 4.158481 0.608429 -1.703195
C 0 -0.168670 1.178600 0.743129
O 0 0.687963 1.868692 -0.141554
C 0 1.512148 2.788810 0.539329
H 0 -1.512955 -0.976531 -2.959863
H 0 0.099761 0.122608 -1.820838
H 0 0.626257 -1.436456 0.599735
H 0 -2.039123 -3.469723 -2.256345
H 0 1.448327 -3.463942 -0.364657
H 0 -0.037779 -2.460125 -3.943299
H 0 -0.054345 -4.182358 -3.450517
H 0 -3.635039 -1.746385 -1.842627
H 0 -1.496367 3.129047 -0.422531
H 0 -3.338201 4.182358 -1.699800
H 0 -5.033691 2.781878 -2.840506
H 0 -4.890306 0.298186 -2.706447
H 0 -4.251350 -3.056345 0.254477
H 0 -3.934734 -3.393302 2.702113
H 0 -2.311522 -1.990365 3.943299
H 0 -0.995870 -0.266815 2.774092
H 0 4.600638 0.824587 0.363679
H 0 5.033691 1.147079 -2.054055
H 0 3.472346 0.210079 -2.444114
H 0 0.439016 0.571999 1.427072
H 0 -0.745444 1.893591 1.355515
H 0 2.188149 3.225724 -0.198010
H 0 2.097654 2.285930 1.322212
H 0 0.909882 3.586212 1.000807
Number of imaginary frequencies: 0

TS-2 [6d (*gauche_s-trans*)] ⇋ [6d (*anti_s-trans*)]
M06-2X/6-31+G(d) Geometry
C 0 -0.741854 -1.956357 -2.024997
C 0 0.106227 -0.818882 -1.364850
C 0 1.406125 -1.305130 -0.676352
C 0 -0.040468 -3.316563 -2.068995
O 0 0.674215 -3.559725 -0.859578
C 0 1.802728 -2.744112 -1.015259
C 0 1.095874 -3.367884 -3.095848
O 0 2.204577 -2.851016 -2.367964
C 0 -0.789950 0.014897 -0.335021
C 0 -1.223973 -1.019213 0.724972
C 0 -1.890941 -2.127788 0.179634
C 0 -2.113669 -2.033146 -1.304283
C 0 -2.777051 -0.711996 -1.583354
C 0 -2.077167 0.406725 -1.097160
C 0 -2.615778 1.671670 -1.351566
C 0 -3.809955 1.799475 -2.067202
C 0 -4.486249 0.683113 -2.544943
C 0 -3.959379 -0.584151 -2.300890
C 0 -2.314242 -3.181904 0.977222
C 0 -2.080041 -3.146382 2.351567
C 0 -1.428634 -2.050467 2.906714
C 0 -0.998479 -0.992613 2.100165
O 0 2.469328 -0.428485 -1.090134
C 0 3.533192 -0.333982 -0.272082
O 0 3.643344 -0.957290 0.760000
C 0 4.551679 0.633138 -0.754304
C 0 4.429752 1.335232 -1.880310
C 0 0.099165 1.144651 0.255186
O 0 -0.344502 2.455809 -0.011878
C 0 0.556881 3.409909 0.494916
H 0 -0.955769 -1.671605 -3.061969
H 0 0.414267 -0.118515 -2.148316
H 0 1.316953 -1.254936 0.414732
H 0 -0.765418 -4.125834 -2.194436
H 0 2.605241 -3.097503 -0.365202
H 0 0.912739 -2.745517 -3.976794
H 0 1.312624 -4.395944 -3.406351
H 0 -2.706011 -2.872303 -1.686669
H 0 -2.121971 2.561546 -0.995681
H 0 -4.211394 2.793376 -2.244651
H 0 -5.415442 0.794091 -3.096595
H 0 -4.468662 -1.475595 -2.660803
H 0 -2.826189 -4.028441 0.524987
H 0 -2.406742 -3.967214 2.983126
H 0 -1.247240 -2.009889 3.976794
H 0 -0.496147 -0.160797 2.581461
H 0 5.415442 0.719514 -0.102230
H 0 5.202603 2.031526 -2.191380
H 0 3.559495 1.224975 -2.520158
H 0 1.112198 1.052924 -0.153384
H 0 0.191131 1.032406 1.341493
H 0 0.155341 4.395944 0.254297
H 0 1.550177 3.297272 0.035645
H 0 0.660062 3.316268 1.585986
Number of imaginary frequencies: 1 (-156.0)

6d (*anti_s-trans*)
M06-2X/6-31+G(d) Geometry
O 0 3.725478 -0.010816 0.392527
O 0 2.506428 0.061720 -1.501953
C 0 3.547591 0.415138 -0.726690
C 0 4.449321 1.398137 -1.379402
C 0 4.253268 1.880745 -2.605911
C 0 1.547783 -0.843697 -0.932066
C 0 2.043911 -2.282989 -1.029335
O 0 2.321013 -2.646006 -2.369915
C 0 1.223064 -3.428366 -2.830343
C 0 0.164753 -3.190888 -1.748185
C 0 -0.613548 -1.891488 -1.983301
C 0 0.219421 -0.600565 -1.670913
O 0 0.993129 -3.105713 -0.590219
C 0 -0.679977 0.414624 -0.854333
C 0 -1.967996 -1.821854 -1.212319
C 0 0.072479 1.720813 -0.648165
C 0 -1.972577 0.572501 -1.646990
C 0 -2.676201 -0.628403 -1.810251
C 0 -3.864151 -0.663633 -2.529104
C 0 -4.361862 0.514331 -3.091371
C 0 -3.662413 1.708693 -2.937004
C 0 -2.466254 1.742894 -2.214834
C 0 -1.025366 -0.309671 0.443097
C 0 -1.686652 -1.527925 0.239874
C 0 -2.010919 -2.346278 1.314247
C 0 -1.674120 -1.945165 2.608200
C 0 -1.016907 -0.734398 2.814388

C 0	-0.688978	0.087704	1.733146
O 0	-0.740675	2.636251	0.046251
C 0	-0.062392	3.835943	0.332517
H 0	5.294228	1.693047	-0.764891
H 0	3.404985	1.564846	-3.205472
H 0	4.942389	2.598778	-3.039796
H 0	1.452164	-0.629454	0.137458
H 0	2.926118	-2.451345	-0.408243
H 0	1.517445	-4.482777	-2.878364
H 0	0.922814	-3.080513	-3.822379
H 0	-0.510665	-4.040014	-1.610468
H 0	-0.876854	-1.872746	-3.047281
H 0	0.477311	-0.123703	-2.623101
H 0	-2.537810	-2.748056	-1.348191
H 0	0.996770	1.542648	-0.074386
H 0	0.374200	2.137652	-1.625506
H 0	-4.399576	-1.602784	-2.650549
H 0	-5.294228	0.497697	-3.648365
H 0	-4.052542	2.624781	-3.371162
H 0	-1.943094	2.683651	-2.075843
H 0	-2.519884	-3.292560	1.144508
H 0	-1.924935	-2.577922	3.454584
H 0	-0.757734	-0.423753	3.822379
H 0	-0.189460	1.037031	1.903267
H 0	-0.758473	4.482777	0.869035
H 0	0.263682	4.337855	-0.590945
H 0	0.821146	3.649959	0.961531

Number of imaginary frequencies: 0

TS-3 [6d (*anti_s-trans*)] ⇋ [6c (*anti_s-cis*)]

M06-2X/6-31+G(d) Geometry

C 0	-0.676537	3.882034	0.045325
O 0	-1.290968	2.635935	-0.176847
C 0	-0.425638	1.722991	-0.809204
C 0	-1.106077	0.368344	-0.936995
C 0	-0.143802	-0.647377	-1.678127
C 0	-0.907196	-1.994787	-1.920326
C 0	-2.272590	-1.950214	-1.166074
C 0	-2.023532	-1.552100	0.267460
C 0	-1.429702	-0.289621	0.399681
C 0	1.185702	-0.780830	-0.910996
C 0	1.746637	-2.201126	-0.893588
O 0	0.723423	-3.043518	-0.429106
C 0	-0.069665	-3.235864	-1.597912
C 0	-3.033698	-0.834871	-1.844817
C 0	-2.394281	0.408624	-1.750577
O 0	2.083034	-2.640128	-2.196529
C 0	1.024090	-3.482565	-2.643395
C 0	-2.314935	-2.318236	1.388591
C 0	-2.013542	-1.820633	2.657463
C 0	-1.424248	-0.565497	2.792772
C 0	-1.128888	0.204184	1.664794
C 0	-2.938472	1.514574	-2.396086
C 0	-4.119968	1.373213	-3.128850
C 0	-4.754764	0.136691	-3.216095
C 0	-4.206666	-0.976717	-2.574884
O 0	2.112894	0.119392	-1.545324
C 0	3.164353	0.526854	-0.815580
C 0	4.015929	1.495640	-1.575448
C 0	5.055137	1.106109	-2.305978
O 0	3.374809	0.171441	0.319266
H 0	-1.412216	4.525782	0.530128
H 0	-0.358565	4.342913	-0.902175
H 0	0.203075	3.778497	0.698963
H 0	-0.138915	2.092814	-1.809388
H 0	0.501630	1.630581	-0.219755
H 0	0.105040	-0.216583	-2.654153
H 0	-1.157461	-2.053851	-2.985990
H 0	-2.791301	-2.912019	-1.248778
H 0	1.071083	-0.487575	0.138108
H 0	2.615574	-2.283885	-0.237903
H 0	-0.705081	-4.109322	-1.425966
H 0	0.734638	-3.193357	-3.657373
H 0	1.358823	-4.525782	-2.635029
H 0	-2.771095	-3.299114	1.274074
H 0	-2.240388	-2.412472	3.539460
H 0	-1.194022	-0.178826	3.781368
H 0	-0.681589	1.187187	1.780008
H 0	-2.465109	2.487548	-2.310463
H 0	-4.550073	2.238467	-3.625110
H 0	-5.677083	0.037461	-3.781368
H 0	-4.691646	-1.947969	-2.644880
H 0	3.746528	2.544815	-1.472293
H 0	5.677083	1.826913	-2.827236
H 0	5.317192	0.056235	-2.405968

Number of imaginary frequencies: 1 (-143.3)

6d (*anti_s-cis*)

M06-2X/6-31+G(d) Geometry

O 0	3.422828	-0.006170	-0.020821
O 0	2.081339	-0.104047	-1.831464
C 0	3.190904	0.279527	-1.173581
C 0	4.065951	1.103732	-2.048467
C 0	5.206570	1.599525	-1.572535
C 0	1.127529	-0.892380	-1.101356
C 0	1.565062	-2.355529	-1.068559
O 0	1.773763	-2.859920	-2.374284
C 0	0.612234	-3.604197	-2.729106
C 0	-0.377858	-3.245778	-1.613938
C 0	-1.133268	-1.946409	-1.907139
C 0	-0.239120	-0.667070	-1.776038
O 0	0.509778	-3.094682	-0.508904
C 0	-1.046790	0.454664	-1.002840
C 0	-2.419336	-1.760343	-1.043850
C 0	-0.245915	1.748051	-0.988465
C 0	-2.391928	0.581222	-1.707500
C 0	-3.140368	-0.604035	-1.699035
C 0	-4.381558	-0.664165	-2.319222
C 0	-4.888764	0.473138	-2.952269
C 0	-4.146336	1.651529	-2.966969
C 0	-2.896175	1.710332	-2.345095
C 0	-1.310519	-0.127872	0.381089
C 0	-2.015375	-1.338612	0.347906
C 0	-2.271272	-2.040460	1.518720
C 0	-1.822581	-1.529442	2.737876
C 0	-1.124069	-0.324656	2.774730
C 0	-0.864194	0.380145	1.596799
O 0	-0.970167	2.750560	-0.316170
C 0	-0.260337	3.963815	-0.250241
H 0	3.728559	1.268201	-3.067063
H 0	5.501939	1.401109	-0.545545
H 0	5.862923	2.205025	-2.189125
H 0	1.116902	-0.555501	-0.059910
H 0	2.468180	-2.493151	-0.470465
H 0	0.850232	-4.673591	-2.727217

H 0 0.276262 -3.300632 -3.724602
H 0 -1.068069 -4.060224 -1.375930
H 0 -1.479105 -2.012142 -2.945446
H 0 -0.029533 -0.292236 -2.784020
H 0 -3.023331 -2.674650 -1.044170
H 0 0.723077 1.595249 -0.485563
H 0 -0.027200 2.060167 -2.025009
H 0 -4.952262 -1.590182 -2.309106
H 0 -5.862923 0.437046 -3.431624
H 0 -4.544075 2.536062 -3.456194
H 0 -2.335995 2.639989 -2.338751
H 0 -2.814865 -2.981968 1.481740
H 0 -2.019292 -2.071939 3.658067
H 0 -0.777325 0.071883 3.724602
H 0 -0.328057 1.324022 1.634279
H 0 -0.881535 4.673591 0.298495
H 0 -0.057342 4.359937 -1.256825
H 0 0.697436 3.835141 0.276047
Number of imaginary frequencies: 0

TS-4 [6d (*anti_s-cis*)] ⇋ [6d (*gauche_s-cis*)]

M06-2X/6-31+G(d) Geometry
C 0 -1.206818 -2.029848 -2.022473
C 0 -0.328641 -0.840189 -1.519359
C 0 1.016620 -1.263600 -0.876159
C 0 -0.481550 -3.375264 -2.026957
O 0 0.289467 -3.535920 -0.839946
C 0 1.405519 -2.730425 -1.102727
C 0 0.604548 -3.466679 -3.108530
O 0 1.760379 -2.957294 -2.451911
C 0 -1.163874 0.074556 -0.507281
C 0 -1.499332 -0.862935 0.670570
C 0 -2.179190 -2.027715 0.278799
C 0 -2.514754 -2.070596 -1.186870
C 0 -3.226951 -0.788947 -1.525437
C 0 -2.511916 0.378825 -1.202408
C 0 -3.091755 1.607708 -1.530877
C 0 -4.342572 1.654015 -2.154036
C 0 -5.035758 0.490007 -2.465030
C 0 -4.466393 -0.742714 -2.150131
C 0 -2.516493 -3.011502 1.197641
C 0 -2.182797 -2.846396 2.541648
C 0 -1.519525 -1.692926 2.945769
C 0 -1.175313 -0.706399 2.017172
O 0 2.045240 -0.431300 -1.441977
C 0 3.171461 -0.276944 -0.722017
O 0 3.355031 -0.789668 0.358735
C 0 4.129715 0.615217 -1.426310
C 0 5.291860 0.927629 -0.855961
C 0 -0.255625 1.263330 -0.087954
O 0 -0.742939 2.539575 -0.436204
C 0 0.163391 3.548399 -0.062325
H 0 -1.509778 -1.824662 -3.056187
H 0 -0.076351 -0.208135 -2.377230
H 0 1.001040 -1.108255 0.208470
H 0 -1.195572 -4.202611 -2.073110
H 0 2.233592 -3.024111 -0.455814
H 0 0.381773 -2.859354 -3.991343
H 0 0.791811 -4.502683 -3.410189
H 0 -3.114882 -2.949557 -1.447860
H 0 -2.585719 2.532144 -1.303433
H 0 -4.774678 2.622200 -2.391569
H 0 -6.009034 0.539022 -2.945044
H 0 -4.986168 -1.669508 -2.383149
H 0 -3.039575 -3.905068 0.864171
H 0 -2.442260 -3.611806 3.267239
H 0 -1.262072 -1.550805 3.991343
H 0 -0.659330 0.174993 2.382148
H 0 3.831808 0.982209 -2.403539
H 0 6.009034 1.577347 -1.347148
H 0 5.544441 0.529361 0.123216
H 0 0.727540 1.146266 -0.558961
H 0 -0.084611 1.250470 0.994421
H 0 -0.267922 4.502683 -0.369879
H 0 1.135408 3.410635 -0.558714
H 0 0.321217 3.554510 1.026200
Number of imaginary frequencies: 1 (-155.7)

7 (*s-cis*)
M06-2X/6-31+G(d) Geometry

C 0	-2.493808	0.129280	-0.000170
O 0	-1.217414	-0.510698	-0.000370
C 0	-0.160009	0.319133	-0.000842
O 0	-0.260881	1.524278	-0.001009
C 0	1.117833	-0.441067	-0.001081
C 0	2.279997	0.209018	0.000110
H 0	-3.226823	-0.675942	-0.000307
H 0	-2.602925	0.753591	-0.890104
H 0	-2.602840	0.753131	0.890104
H 0	1.046989	-1.524278	-0.002109
H 0	3.226823	-0.321240	0.000038
H 0	2.298281	1.295686	0.001113

Number of imaginary frequencies: 0

7 (*s-trans*)
M06-2X/6-31+G(d) Geometry

C 0	-2.453426	-0.579379	0.000000
O 0	-1.026133	-0.622899	-0.001306
C 0	-0.417854	0.575526	-0.000577
O 0	-1.020668	1.624586	0.000434
C 0	1.064493	0.480460	-0.001014
C 0	1.731343	-0.673038	0.000437
H 0	-2.778871	-1.618520	0.000892
H 0	-2.816837	-0.060614	-0.890131
H 0	-2.815195	-0.059499	0.890131
H 0	1.565442	1.443663	-0.002168
H 0	2.816837	-0.691462	0.000435
H 0	1.208500	-1.624586	0.001717

Number of imaginary frequencies: 0

8
M06-2X/6-31+G(d) Geometry

C 0	3.018902	-0.743474	-0.105111
O 0	2.146402	0.369520	-0.110404
C 0	0.813039	0.133860	-0.117312
C 0	0.237375	-1.138098	-0.123743
C 0	-1.152865	-1.262839	-0.131155
C 0	-1.959318	-0.133575	-0.133773
C 0	-1.379444	1.140275	-0.127084
C 0	-0.003747	1.274910	-0.119291
C 0	-3.452834	-0.247828	-0.114849
F 0	-4.027728	0.519618	-1.061660
F 0	-3.975360	0.156381	1.061660
F 0	-3.873694	-1.508445	-0.315233
H 0	4.027728	-0.331943	-0.096467
H 0	2.865738	-1.359000	0.789315
H 0	2.880157	-1.356527	-1.003591
H 0	0.848863	-2.032511	-0.124476
H 0	-1.602061	-2.250750	-0.138199
H 0	-2.012421	2.023140	-0.130696
H 0	0.470807	2.250750	-0.116190

Number of imaginary frequencies: 0

9
M06-2X/6-31+G(d) Geometry

C 0	3.656972	0.332355	-0.000262
O 0	2.679035	-0.682588	0.000154
C 0	1.369498	-0.295475	0.000163
C 0	0.937915	1.034130	0.000132
C 0	-0.424597	1.320028	-0.000015
C 0	-1.369610	0.295514	-0.000104
C 0	-0.937931	-1.034169	-0.000221
C 0	0.424514	-1.320072	-0.000119
O 0	-2.679101	0.682559	-0.000414
C 0	-3.657047	-0.332652	0.000169
H 0	4.621165	-0.176533	-0.000585
H 0	3.577586	0.961962	-0.895768
H 0	3.578261	0.962043	0.895278
H 0	1.646201	1.855039	0.000016
H 0	-0.774501	2.347736	-0.000076
H 0	-1.646284	-1.855036	-0.000618
H 0	0.774537	-2.347736	-0.000347
H 0	-3.577368	-0.962024	0.895768
H 0	-3.578185	-0.962302	-0.895305
H 0	-4.621165	0.176323	0.000583

Number of imaginary frequencies: 0

10
M06-2X/6-31+G(d) Geometry

C 0	2.563698	0.297550	-0.000074
O 0	1.570167	-0.704293	-0.000749
C 0	0.268683	-0.305871	-0.000580
C 0	-0.144120	1.027762	-0.000603
C 0	-1.510941	1.320975	-0.000455
C 0	-2.460627	0.306638	-0.000286
C 0	-2.035063	-1.025152	-0.000293
C 0	-0.682274	-1.333384	-0.000394
H 0	3.519582	-0.226357	0.000367
H 0	2.493155	0.927194	-0.895690
H 0	2.492164	0.926933	0.895690
H 0	0.575823	1.838307	-0.000772
H 0	-1.825600	2.360875	-0.000441
H 0	-3.519582	0.544752	-0.000187
H 0	-2.764675	-1.830140	-0.000088
H 0	-0.331787	-2.360875	-0.000496

Number of imaginary frequencies: 0

Complex 7(*s-cis*)+8
M06-2X/6-31+G(d) Geometry

C 0	3.634671	-1.231417	1.045673
O 0	2.353552	-1.849160	0.910191
C 0	1.773018	-1.725878	-0.293067
O 0	2.286915	-1.137955	-1.221282
C 0	0.447262	-2.393687	-0.322534
C 0	-0.291853	-2.363844	-1.429830
H 0	3.975944	-1.483705	2.048752
H 0	3.535386	-0.149656	0.932078
H 0	4.323909	-1.621830	0.293368
H 0	0.118549	-2.874688	0.593568
H 0	-1.271804	-2.828746	-1.472091
H 0	0.079269	-1.860206	-2.318901
C 0	2.342533	1.952042	-0.699294
O 0	1.695613	1.382797	0.428371
C 0	0.409784	0.985031	0.285710
C 0	-0.329614	1.120403	-0.891228
C 0	-1.644254	0.657383	-0.930726
C 0	-2.219287	0.071925	0.190596
C 0	-1.479296	-0.056762	1.369080
C 0	-0.172581	0.396915	1.418280
C 0	-3.606422	-0.487763	0.133538
F 0	-4.295927	-0.251359	1.265253
F 0	-3.602068	-1.831417	-0.028461
F 0	-4.323909	0.015932	-0.884930
H 0	3.355642	2.187305	-0.371950

H 0 2.379859 1.232581 -1.523336
H 0 1.837847 2.874688 -1.009322
H 0 0.106700 1.560828 -1.779576
H 0 -2.219688 0.756578 -1.846215
H 0 -1.929681 -0.513095 2.246105
H 0 0.426290 0.304008 2.318901
Number of imaginary frequencies: 0

Complex 7(*s-trans*)+8
M06-2X/6-31+G(d) Geometry
C 0 3.471652 -1.336117 0.976392
O 0 2.230899 -1.857309 0.495039
C 0 1.918710 -1.533091 -0.767592
O 0 2.641523 -0.866171 -1.479361
C 0 0.605507 -2.063357 -1.207913
C 0 -0.239928 -2.705943 -0.402829
H 0 3.593643 -1.751934 1.975716
H 0 3.422971 -0.245293 1.013107
H 0 4.291248 -1.649950 0.325980
H 0 0.372961 -1.850913 -2.247111
H 0 -1.204037 -3.052105 -0.762051
H 0 0.007688 -2.890941 0.638175
C 0 2.463998 2.128687 -0.622305
O 0 1.794351 1.446712 0.428403
C 0 0.515842 1.055647 0.220698
C 0 -0.163450 1.200306 -0.991143
C 0 -1.479498 0.748398 -1.098142
C 0 -2.109934 0.158564 -0.011756
C 0 -1.426771 0.014223 1.201268
C 0 -0.123479 0.460361 1.318872
C 0 -3.507319 -0.368177 -0.116398
F 0 -4.291248 0.073065 0.886278
F 0 -3.542810 -1.718990 -0.047571
F 0 -4.104274 -0.024835 -1.269634
H 0 3.451682 2.373376 -0.231156
H 0 2.568244 1.482515 -1.498750
H 0 1.931847 3.052105 -0.879686
H 0 0.319855 1.645352 -1.852839
H 0 -2.008465 0.857516 -2.039679
H 0 -1.922967 -0.447819 2.050635
H 0 0.429636 0.357112 2.247111
Number of imaginary frequencies: 0

Complex 7(*s-cis*)+9
M06-2X/6-31+G(d) Geometry
C 0 -4.069016 -1.225787 -1.167277
O 0 -2.809699 -1.864235 -0.953131
C 0 -2.279494 -1.704245 0.269509
O 0 -2.825649 -1.081950 1.155300
C 0 -0.965817 -2.387188 0.377372
C 0 -0.259835 -2.307012 1.503444
H 0 -4.367669 -1.499708 -2.178341
H 0 -3.951140 -0.143797 -1.079126
H 0 -4.801241 -1.581123 -0.438317
H 0 -0.614541 -2.913682 -0.504655
H 0 0.714854 -2.775164 1.600604
H 0 -0.648608 -1.751270 2.352721
C 0 -2.737163 1.984373 0.419191
O 0 -2.050815 1.314090 -0.621094
C 0 -0.770865 0.912210 -0.369638
C 0 -0.109717 1.094573 0.848193
C 0 1.189943 0.623962 1.005914
C 0 1.847398 -0.027182 -0.038263
C 0 1.191371 -0.199573 -1.258965
C 0 -0.112116 0.266301 -1.415167
O 0 3.114177 -0.464940 0.228954
C 0 3.819639 -1.089696 -0.819585
H 0 -3.723697 2.225351 0.020202
H 0 -2.846291 1.335315 1.294707
H 0 -2.220920 2.913682 0.691553
H 0 -0.599764 1.580921 1.683902
H 0 1.713400 0.751906 1.948749
H 0 1.673066 -0.700043 -2.091656
H 0 -0.641638 0.124908 -2.352721
H 0 3.938179 -0.414351 -1.676498
H 0 3.315614 -2.008097 -1.147891
H 0 4.801241 -1.340367 -0.416759
Number of imaginary frequencies: 0

Complex 7(*s-trans*)+9
M06-2X/6-31+G(d) Geometry
C 0 3.788549 -1.485751 1.214461
O 0 2.596930 -1.970176 0.592642
C 0 2.405874 -1.573176 -0.673537
O 0 3.204672 -0.894284 -1.284506
C 0 1.125929 -2.047515 -1.254115
C 0 0.199995 -2.707190 -0.559614
H 0 3.806531 -1.941785 2.203858
H 0 3.745933 -0.396779 1.289009
H 0 4.665802 -1.785608 0.636546
H 0 0.986402 -1.772468 -2.295131
H 0 -0.736805 -3.007101 -1.020754
H 0 0.352608 -2.949169 0.487814
C 0 2.926612 2.072270 -0.328434
O 0 2.198364 1.338889 0.639754
C 0 0.923668 0.967923 0.325853
C 0 0.322242 1.174292 -0.920667
C 0 -0.980044 0.737044 -1.140865
C 0 -1.694527 0.085521 -0.137074
C 0 -1.096434 -0.114884 1.110900
C 0 0.203989 0.328415 1.333683
O 0 -2.955482 -0.326596 -0.465313
C 0 -3.704661 -0.985324 0.530458
H 0 3.887269 2.301989 0.134343
H 0 3.093780 1.473733 -1.229983
H 0 2.408218 3.007101 -0.576743
H 0 0.860351 1.660620 -1.726776
H 0 -1.457276 0.885294 -2.104824
H 0 -1.625013 -0.614868 1.915111
H 0 0.684092 0.172005 2.295131
H 0 -3.865909 -0.337904 1.401857
H 0 -3.211045 -1.911745 0.853102
H 0 -4.665802 -1.226530 0.075821
Number of imaginary frequencies: 0

Complex 7(*s-cis*)+10
M06-2X/6-31+G(d) Geometry
C 0 3.176517 -0.071642 0.695544
O 0 2.115770 -1.027484 0.711550
C 0 1.448746 -1.176714 -0.443265
O 0 1.724402 -0.567700 -1.454901
C 0 0.357591 -2.174114 -0.306647
C 0 -0.435599 -2.444986 -1.341187
H 0 3.661866 -0.157355 1.666988
H 0 2.763719 0.929982 0.557935
H 0 3.879095 -0.298989 -0.109570

H 0	0.233763	-2.634259	0.668582
H 0	-1.250850	-3.156578	-1.259216
H 0	-0.282343	-1.945494	-2.294123
C 0	0.924949	2.422766	-1.035404
O 0	0.527387	1.753519	0.148242
C 0	-0.629722	1.038743	0.111228
C 0	-1.435776	0.909398	-1.020673
C 0	-2.601134	0.142791	-0.945385
C 0	-2.969392	-0.489389	0.237905
C 0	-2.152458	-0.356158	1.363036
C 0	-0.988477	0.398829	1.304054
H 0	1.850890	2.943396	-0.786911
H 0	1.113866	1.705618	-1.840848
H 0	0.168431	3.156578	-1.339696
H 0	-1.163026	1.379895	-1.958200
H 0	-3.224029	0.045672	-1.830709
H 0	-3.879095	-1.080043	0.284961
H 0	-2.422421	-0.846997	2.294123
H 0	-0.333989	0.505302	2.164396

Number of imaginary frequencies: 0

Complex 7(*s-trans*)+10

M06-2X/6-31+G(d) Geometry

C 0	2.895925	-0.389013	1.125026
O 0	1.879781	-1.226734	0.571744
C 0	1.567179	-0.986913	-0.709546
O 0	2.126644	-0.147354	-1.383963
C 0	0.476576	-1.852018	-1.222981
C 0	-0.189402	-2.725823	-0.469436
H 0	3.062757	-0.763044	2.134623
H 0	2.546624	0.645897	1.146942
H 0	3.810509	-0.460705	0.531732
H 0	0.244552	-1.689579	-2.271161
H 0	-0.996075	-3.322218	-0.884707
H 0	0.046985	-2.854631	0.582234
C 0	0.892461	2.573746	-0.582201
O 0	0.485553	1.709949	0.465027
C 0	-0.641990	0.972917	0.279197
C 0	-1.384791	0.955977	-0.903793
C 0	-2.523838	0.150278	-0.983906
C 0	-2.922934	-0.634687	0.091223
C 0	-2.169325	-0.611097	1.269666
C 0	-1.039204	0.186759	1.367526
H 0	1.793764	3.071236	-0.222284
H 0	1.132321	2.005452	-1.486204
H 0	0.117379	3.322218	-0.787937
H 0	-1.087115	1.553282	-1.758310
H 0	-3.098721	0.142818	-1.905878
H 0	-3.810509	-1.255904	0.020018
H 0	-2.468432	-1.218371	2.119738
H 0	-0.437533	0.213603	2.271161

Number of imaginary frequencies: 0
